# Natural Products in Cancer Prevention and Therapy: Current Challenges and Future Directions

**DOI:** 10.1002/mco2.70585

**Published:** 2026-01-28

**Authors:** Ruimiao Qian, Jun Ge, Ni Fan, Zheng Sun, Chengcheng Zhao, Yujiao Sun, Yingpeng Li, Yunfei Li, Hui Fu

**Affiliations:** ^1^ College of Chinese Materia Medica Tianjin University of Traditional Chinese Medicine Tianjin China; ^2^ Experimental Teaching and Practical Training Center Heilongjiang University of Chinese Medicine Harbin China; ^3^ College of Integrated Chinese and Western Medicine Tianjin University of Traditional Chinese Medicine Tianjin China

**Keywords:** cancer metabolic reprogramming, immune modulation, natural products, precision medicine, tumor microenvironment

## Abstract

Natural products, originating from diverse biological sources, serve as a critical reservoir of bioactive compounds for cancer intervention across prevention, treatment, and supportive care. Their mechanisms extend beyond direct cytotoxicity to include modulation of tumor metabolism—such as glucose, lipid, and glutamine pathways—and the tumor microenvironment (TME), highlighting their multifaceted role in oncology. However, a systematic synthesis of how natural products concurrently target metabolic reprogramming and immune–stromal components across different clinical phases remains lacking. This review delineates the therapeutic applications of natural products—such as flavonoids, alkaloids, and terpenoids—across the clinical continuum, including perioperative support, concurrent chemoradiotherapy, maintenance therapy, and metastasis suppression. We detail their actions in disrupting core metabolic pathways and elucidate their influence on TME components like cancer‐associated fibroblasts, extracellular matrix, and immune cells including tumor‐associated macrophages and T lymphocytes. Furthermore, we discuss innovative delivery strategies—including nanocarriers and codelivery systems—that enhance bioavailability and enable synergistic combination with chemotherapy or immunotherapy. By integrating mechanistic insights with clinical translation strategies, this work provides a comprehensive framework for employing natural products in biomarker‐driven, precision oncology regimens, supporting their evolving role in multimodal cancer care.

## Graphical abstract

Natural products play a pivotal role in cancer therapy. They induce cancer cell death by reprogramming four metabolic pathways while precisely targeting the tumor microenvironment and immune cells. These compounds not only leverage novel delivery systems for innovative applications but also demonstrate unique therapeutic efficacy across clinical stages: primary prevention, concurrent treatment phase, postoperative recovery, maintenance therapy, and end‐of‐life care.

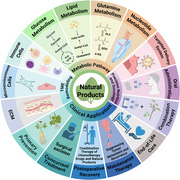

## 1 Introduction

In addition to conventional cancer treatments such as surgery, radiotherapy, and chemotherapy, clinical management is increasingly exploring multitarget, low‐toxicity adjunctive strategies. Natural products—bioactive compounds derived from plants, microorganisms, and marine organisms—offer significant therapeutic potential in this regard, owing to their chemical diversity and multifaceted biological activities [1].

**FIGURE 1:**
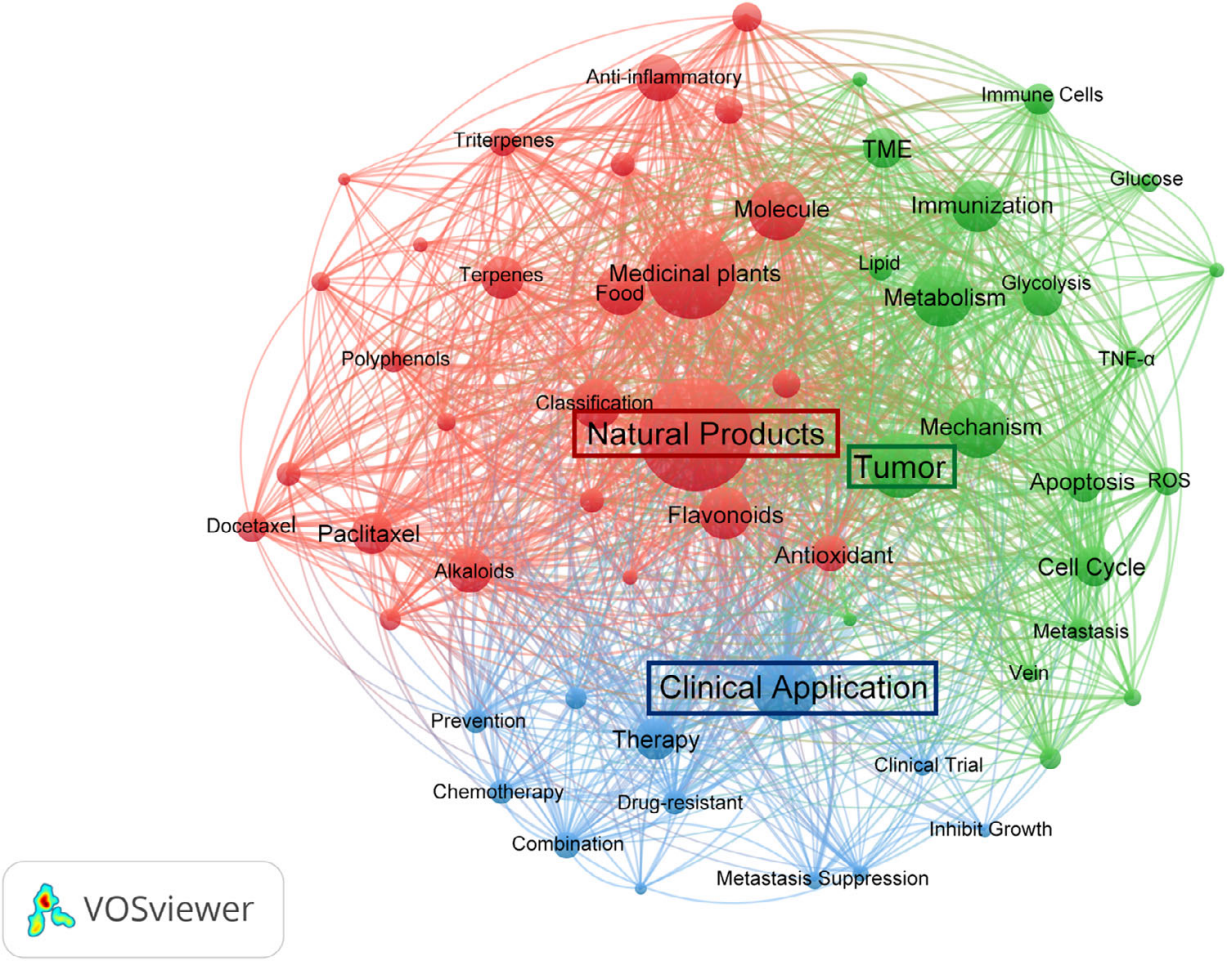
Co‐occurrence network analysis of natural product‐related research using VOSviewer. This network visualization illustrates the central role of natural products in cancer therapy, highlighting their connections to concepts such as clinical applications, tumor metabolism, immune regulation, and mechanisms of action. Key clusters are color coded: red represents natural product sources and classification; green emphasizes cancer biology, related mechanisms, and metabolic processes; and blue denotes tumor clinical treatment and applications.

Natural products exhibit broad applications throughout the cancer treatment continuum. During the perioperative phase, natural products serve as adjuncts to chemotherapy and radiotherapy. They not only enhance cancer cell sensitivity—for example, apigenin increases pancreatic cancer cells’ sensitivity to gemcitabine—but also mitigate the toxic side effects of conventional drugs such as cisplatin, capecitabine, and fluorouracil [2, 3, 4, 5]. At the same time, certain natural products, such as the active components in rhubarb, can improve patients’ physical condition and reduce inflammatory responses during the perioperative period [6]. In the later stages of treatment and during rehabilitation, natural products play a pivotal role in maintaining therapeutic efficacy, preventing metastasis, and improving overall quality of life [7, 8]. Figure 1 highlights the interconnected roles of natural products in cancer therapy and their clinical applications.

The primary advantage of natural products lies in their ability to address the limitations of conventional cancer therapies. Drugs like cisplatin and oxaliplatin often induce nephrotoxicity, neurotoxicity, and multidrug resistance. Natural compounds such as the organosulfur compounds, present in garlic, can reverse platinum resistance by downregulating P‐glycoprotein (P‐gp) on tumor cells, thereby enhancing intracellular drug accumulation and therapeutic efficacy [9]. Antimetabolites like 5‐fluorouracil suffer from narrow therapeutic windows, significant toxicity, and extensive first‐pass metabolism [10]. Research suggests that chemically linking 5‐fluorouracil with the natural product curcumin (CUR) to form new derivatives can potentiate their synergistic effects while minimizing adverse reactions [11]. Taxol, derived from the yew tree, exhibits significant efficacy against various malignancies by targeting tumor cell mitosis and inducing apoptosis, and it is now widely used after being synthesized via semi‐synthetic techniques and synthetic biology [12]. Additionally, natural products such as astragaloside IV, matrine, and ginsenosides feature novel structures, multiple targets, diverse pathways, minimal toxicity, and multifaceted biological activities. These properties enable them to exert unique antitumor effects, particularly by modulating tumor metabolism and regulating the tumor immune microenvironment (TIME) to generate antitumor activity [13].

Mechanistically, tumor metabolic reprogramming plays a pivotal role as a core component, underpinning effects such as directly inducing tumor cell apoptosis, inhibiting metastasis, and counteracting oxidative stress. For instance, dietary flavonoids suppress glucose, lipid, and glutamine metabolism in cancer cells, disrupting their biosynthesis and energy supply while inhibiting cell proliferation [14]. Furthermore, natural products profoundly reshape the tumor microenvironment (TME) by modulating the functions of cancer‐associated fibroblast (CAFs), tumor‐associated macrophages (TAMs), T lymphocytes, and other immune cells [15]. This remodeling reverses immunosuppression, thereby activating the body's innate antitumor immune response.

However, translating the vast potential of natural products into effective clinical therapies remains a considerable challenge. Major issues include low oral bioavailability, complex in vivo metabolism, and substantial patient variability, which can significantly influence therapeutic efficacy. To overcome these challenges, cutting‐edge technologies, including nanocarriers and targeted delivery systems, are being extensively explored [16, 17]. Additionally, with the advancement of precision medicine, biomarker‐driven patient stratification strategies are essential for tailoring natural product‐based therapies to individual patients.

This review approaches the topic from the overarching framework of natural products in cancer prevention and therapy, outlining their sources, structural classifications, and functional roles across different clinical stages. At the mechanistic level, particular emphasis is placed on the antitumor effects mediated through metabolic reprogramming and modulation of the TIME, with detailed analysis of their multitarget regulation of glucose, lipid, and glutamine metabolic pathways. In parallel, the review examines the current status of clinical translation, summarizing key challenges such as limited bioavailability, formulation barriers, and patient‐specific variability, while highlighting emerging opportunities including nanodelivery platforms, combination therapeutic strategies, and precision medicine‐oriented applications. Collectively, this work provides an integrated perspective to advance the scientific application and future development of natural products in oncology.

## 2 Classification and Clinical Context of Natural Products for Therapeutic Application

Natural products hold significant value in clinical cancer treatment due to their diverse taxonomic classification and stage‐specific applications [18]. These compounds are systematically categorized based on origin and chemical structure, encompassing flavonoids, alkaloids, terpenoids, polyphenols, and other classes, each exhibiting unique biological activities [19]. Moreover, they demonstrate multitarget regulatory functions throughout the entire clinical cancer treatment process—from primary prevention, perioperative support, and concurrent chemoradiotherapy to postoperative recovery, maintenance therapy, and end‐of‐life care. This exploration of chemical taxonomy and clinical‐stage applications aids in understanding the application strategies of natural products in precision oncology.

### 2.1 Sources and Classifications of Natural Products

Natural products can be categorized into food‐derived compounds and medicinal‐derived compounds based on their sources and intended applications. Food‐derived compounds, such as lycopene in tomatoes and anthocyanins in blueberries, originate from the daily diet. Their core value lies in providing nutrition and maintaining physiological homeostasis, emphasizing long‐term, low‐dose disease prevention [20]. These compounds are typically consumed through regular diets or dietary supplements and are generally considered to have relatively high safety profiles. In contrast, medicinal‐source compounds, such as paclitaxel, ginsenosides, and berberine, are derived from medicinal plants with well‐defined pharmacological activities [21, 22, 23]. Their primary development targets disease treatment directly, requiring extraction, purification, and formulation into specific pharmaceuticals to enable high‐dose, phased therapeutic interventions. However, these compounds may carry potential risks, including hepatic, renal, or neurotoxicity [24]. It is important to note that these two categories are not entirely distinct. Many substances, such as CUR, are “food‐medicine dual‐use” compounds, serving both as everyday seasonings and as potential drug candidates, representing a continuum of intervention strategies from dietary prevention to targeted therapy [25].

It is noteworthy that there exists an intrinsic connection between the origin of natural products and their core mechanisms of action, profoundly influencing their positioning within disease intervention strategies. Compounds derived from medicinal sources typically target critical direct targets essential for the survival of rapidly proliferating cells, exhibiting potent cytotoxic effects [26]. For example, Halorotetin A, a novel terpenoid compound isolated from the ascidian Halocynthia rotetzi, significantly inhibits the proliferation of the hepatocellular carcinoma cell line HepG‐2. Its mechanism of action is closely associated with regulating the expression of oncogenes (such as c‐myc and c‐met) and tumor suppressor genes (such as TP53 and KEAP1), demonstrating the ability to directly intervene in tumorigenesis pathways [27]. In contrast, compounds derived from food sources predominantly act on regulatory pathways, indirectly influencing tumor initiation and progression through multitarget mechanisms [28]. For example, the compound isoegomaketone isolated from the plant P. frutescens significantly inhibited cell growth and xenograft tumor formation, potentially by blocking the PI3K/Akt signaling pathway in HCC cells [29]. Asparagus polysaccharides selectively inhibit proliferation in HepG2 and HepG3B cell lines by inducing G/M phase arrest and apoptosis through Bax, Bcl‐2, and caspase‐3 regulation, while exhibiting low cytotoxicity toward normal human hepatocyte 7702 cells [30]. This classification system of mechanisms—ranging from “direct cell killing” to “multidimensional physiological regulation”—establishes a theoretical foundation for the precise application of diverse natural products.

Natural products can also be classified into several important categories based on their chemical structures and biosynthetic pathways. Flavonoids, a diverse group of polyphenolic compounds abundantly present in fruits, vegetables, tea, legumes, and other plant‐based foods, have emerged as significant candidates for cancer prevention and adjuvant therapy [31]. Numerous studies have demonstrated that regular consumption of flavonoid‐rich foods is associated with a reduced incidence of various malignancies, indicating their remarkable potential as dietary chemopreventive agents [32]. All flavonoids share a core structure consisting of two aromatic benzene rings (A and B) connected by a three‐carbon bridge, forming a heterocyclic pyran ring (C), commonly referred to as the C6–C3–C6 skeleton [33]. Their potent biological activities are attributed to their antioxidant properties and their influence on tumor metabolic reprogramming. Representative compounds include quercetin, found in vegetables and fruits, and the widely distributed apigenin, both of which demonstrate multifaceted potential in anticancer research, such as inducing apoptosis, suppressing inflammatory responses, and inhibiting tumor angiogenesis [2, 34].

Alkaloids are naturally occurring organic compounds containing nitrogen atoms. Their use dates back to ancient Greece, where poppies were employed for pain relief. In modern times, alkaloids’ potential in cancer prevention and treatment has been extensively studied, with their anticancer effects often linked to interference with cell division [35]. Vinblastine, extracted from Catharanthus roseus, and its derivative vincristine inhibit microtubule protein polymerization, thereby blocking cancer cell mitosis [36]. Similarly, two derivatives of camptothecin, topotecan and irinotecan, have been widely used in cancer treatment [37].

Terpenoids play a crucial role in chemical communication between plants and their environment. Plants can produce a wide variety of terpenoids, making them the largest class of plant‐derived natural products. Composed of isoprene units, terpenoids exhibit significant antitumor activity and are considered promising agents for antitumor drug development [38]. Paclitaxel, isolated from the Pacific yew tree (Taxus brevifolia), was the first plant‐derived chemotherapeutic agent widely used in oncology and remains one of the most common drugs in this field. It exerts potent cytotoxic effects by stabilizing microtubules and preventing their depolymerization [21].

Natural polyphenols, a broad category of polyhydroxyphenolic compounds, are among the most widely distributed and abundant natural compounds in nature. Flavonoids, phenolic acids, lignans, and stilbenes are the four main structural types of natural polyphenols. In recent years, polyphenols have garnered extensive attention due to their broad therapeutic effects, including anti‐inflammatory, antioxidant, and antitumor activities [39]. Representative compounds include resveratrol from grape skin and epigallocatechin gallate (EGCG) from green tea. These compounds primarily inhibit tumor initiation and development by regulating key signaling pathways, such as NF‐κB and Wnt [40, 41].

Other categories of natural products, such as quinones (e.g., juglone), saponins (e.g., ginsenosides), polysaccharides (e.g., Ganoderma lucidum polysaccharides), and organosulfur compounds (e.g., allicin), also exhibit unique anticancer activities, further enriching the source for anticancer drug research and development [9, 22, 42, 43].

### 2.2 The Functions of Natural Products Across Various Clinical Phases

Throughout the entire course of cancer treatment, natural products demonstrate unique value at different clinical stages due to their multitargeted regulation and relatively low toxicity. They play significant roles in primary prevention, concurrent treatment, postoperative recovery, maintenance therapy, and end‐of‐life care, offering adjunctive treatment strategies distinct from traditional chemotherapy (Table 1) [44, 45, 46, 47, 48, 49, 50, 51, 52, 53, 54, 55, 56, 57].

**TABLE 1:** Representative natural products and their applications across various stages of cancer management.

Disease stage	Natural products	Cancer type	Function	Dosage and administration	Research phase	Mechanism of action	References
Primary prevention	Soy isoflavones	Breast cancer	Reduce the risk of cancer	Oral, 2 servings of soy products daily (containing approximately 50 mg of isoflavone aglycones)	Randomized controlled trial	Competitively binds to estrogen receptors, blocking potent estrogen's procancer signaling; affects levels of risk biomarkers such as IGF‐1, CRP, and leptin	[44]
	Novel purified fraction of almond oil (ricinol, γ‐tocopherol)	LoVo, HT29, and Hep3B cell lines	Chemoprevention of potential cancer	0.001, 0.01, and 0.1 mg/mL	In vitro study	Alter cell cycle characteristics; tumor cell migration activity↓; suppress metabolic pathways	[45]
	*C. pyrenoidosa *water extract	Human endometrial adenocarcinoma cells	Malignancy chemoprevention	50–1000 µg/mL	In vitro study	Cancer cell metabolic activity↓; DNA synthesis capability↓; induces cell death via apoptosis and/or membrane damage	[46]
	Resveratrol	Head and neck cancer	As an adjunct to home enteral nutrition, enhances antioxidant defenses, improves cellular health indicators (e.g., phase angle)	Oral, 400 mg daily of liposomal resveratrol, divided into two doses (5 mL each)	Randomized controlled trial	Glutathione peroxidase activity↑; total antioxidant capacity and superoxide dismutase activity↑; increased oxidative stress, malondialdehyde levels↑; induced cytotoxicity	[47]
Concurrent treatment phase	SH003 (*Astragalus membranaceus*, *Angelica gigas*, and *Trichosanthes kirilowii*)	Lung cancer, breast cancer	Enhance chemotherapy efficacy while reducing toxicity	SH003: oral, 2400–4800 mg/day Docetaxel: intravenous injection, 75 mg/m^2^	Phase I clinical trial	Affects the cell cycle and induces apoptosis; inhibits the Akt/mTOR pathway; cancer cells' antiapoptotic capacity↓; immune modulation↑	[48]
	Quercetin	Breast cancer, prostate cancer	Inhibits lymphocyte tyrosinase activity	1400 mg/m^2^, IV infusion every 3 weeks	Phase I clinical trial	Induces apoptosis and has antiproliferative effects	[49]
	EGCG	Breast, prostate, lung, colorectal cancer	Treatment of COVID‐19 pneumonia	30 mL daily nebulization (1760–8817 µmol/L)	Phase I/II clinical trial	Downregulates inflammatory mediator expression and signaling by acting on STAT1/3 and NF‐κB factors	[50]
	Tanshinone IIA (Tan IIA)	Colorectal cancer	Induces ferroptosis in colorectal cancer cells	20 µM Tan IIA + SLC7A11 plasmid	In vivo study	Suppresses SLC7A11 expression via the PI3K/AKT/mTOR pathway	[51]
Postoperative recovery	Rikkunshito	Gastric cancer	Reduces weight loss, improves nutritional indicators, increases food intake	Oral, 7.5 g/day	Retrospective observational study	Auxin secretion↑, appetite↑; Regulates gastrointestinal motility and hormone levels	[52]
	Ginsenoside Rg1/Rh2	Melanoma (mouse model)	Promotes humoral immunity, enhances T lymphocyte infiltration	Injection, 0.5 or 0.2 mg/kg	In vivo study	Influences Th cell activity, IgA production↑; induces infiltration of CD4^+^ and CD8a^+^ T lymphocytes	[53]
	Icariin	Prostate cancer	Inhibits tumor growth, promotes immunity	Intraperitoneal injection, 10 mg/kg	In vivo study	Amounts of probiotics (such as Lactobacillus and Bifidobacterium)↑; activity of various lymphocyte subsets↑	[54]
Maintenance therapy	Shikonin	CT26 mouse colon cancer cells	Inhibits tumor growth and metastasis, induces cell death	0.16–20 µg/mL	Preclinical study (in vitro and in vivo)	Induces apoptosis and cell cycle arrest; induces novel cell death forms (e.g., ferroptosis, pyroptosis); inhibits tumor metabolism (Warburg effect) by targeting PKM2	[55]
	Berberine	Colorectal cancer	Long‐term prevention of adenoma recurrence, reduces risk of colorectal tumorigenesis	Oral, 0.3 g, twice daily	Randomized controlled trial	Multitargeted regulation of the intestinal microenvironment and inflammatory response.	[56]
End‐of‐life care	Longteng tongluo recipe (LTTL)	Lung cancer	Effectively alleviates lung cancer pain and significantly reduces opioid consumption	Apply LTTL tincture topically, 0.5 mL per cm^2^ per application, twice daily for 8 consecutive days	Randomized controlled trial	Serum miRNA expression levels associated with cancer pain (hsa‐miR‐2110, hsa‐miR‐7d‐3p)↓	[57]

#### 2.2.1 Perioperative Period

During the perioperative period, the use of traditional chemotherapy drugs is often limited due to their significant toxic side effects, making it difficult to employ them safely for adjuvant interventions either preoperatively or shortly after surgery [58]. Furthermore, surgical trauma triggers systemic inflammatory responses and hypercoagulable states, which may create favorable conditions for residual or circulating tumor cells, thus increasing the risk of postoperative recurrence and metastasis [59]. In contrast, natural products hold unique value in this regard.

A recent clinical study in gastric cancer patients demonstrated that initiating oral administration of the Japanese Kampo medicine Rikkunshito immediately after minimally invasive gastrectomy (on postoperative Day 1) effectively mitigated weight loss, improved nutritional indicators (such as PNI and GNRI), and increased postoperative food intake [52]. The mechanism of action is closely linked to the multitargeted properties of natural products: Rikkunshito stimulates appetite by promoting ghrelin secretion and creates a more recovery‐friendly physiological environment by regulating gastrointestinal motility and hormone levels [60]. These findings confirm that natural products serve as a safe perioperative adjunct when combined with postoperative nutritional support regimens. They directly address surgery‐induced anorexia and malnutrition without significant side effects, offering a novel strategy beyond chemotherapy drugs to improve both short‐term outcomes and long‐term prognosis.

#### 2.2.2 Concurrent Chemoradiotherapy

During the combined chemotherapy and radiotherapy phase, conventional treatments face numerous challenges, including multidrug resistance, adverse reactions limiting dosage, and severe immunosuppression, all of which constrain improvements in therapeutic efficacy [61].

Natural products demonstrate multifaceted synergistic advantages at this stage. They serve as radiosensitizers and chemotherapeutic sensitizers, enhancing efficacy while reducing toxicity. For example, a multicenter randomized clinical trial in non‐small cell lung cancer (NSCLC) demonstrated that the compound traditional Chinese medicine formulation Shengbai Oral Solution, when combined with platinum‐based chemotherapy, significantly reduced the incidence of chemotherapy‐induced neutropenia and decreased the use of granulocyte colony‐stimulating factor. Concurrently, this treatment markedly alleviated the burden of core symptoms reported by patients, such as fatigue and pain [62]. Modified peanut skin decoction (PSD) demonstrated efficacy in preventing and treating chemotherapy‐induced bone marrow suppression in patients with advanced squamous cell lung cancer. PSD significantly delayed the onset of Grade III–IV bone marrow suppression, shortened its duration, and improved patients’ quality of life [63]. These provide direct clinical evidence for natural products as chemotherapy adjuvants, enabling synergistic anticancer strategies that enhance efficacy while reducing toxicity.

The steroid alkaloid paimonin (PMI) from Fritillariae Thunbergii Bulbus synergizes with Oxa to significantly enhance cell death and inhibit gastric cancer cell proliferation. This combination activates the mitochondrial apoptosis pathway by upregulating Bax, inducing CYCS release, and cleaving caspase‐9, caspase‐3, and poly(ADP‐ribose) polymerase (PARP). Concurrently, it inhibits the RAS/PI3K/AKT survival pathway by suppressing Ras‐GTP and phosphorylated AKT [64]. Tanshinone IIA synergistically potentiates the cytotoxic effects of Olaparib in both wild‐type and BRCA‐deficient triple‐negative breast cancer cells. It induces apoptosis by increasing double‐strand breaks in these cells and subsequently disrupting ataxia–telangiectasia mutated stability, presenting a potential combination therapy approach with PARP inhibitors [65].

#### 2.2.3 Postoperative and Recovery Phase

Postoperative patients often face challenges such as persistent immunosuppression, cancer‐related fatigue, and intestinal dysfunction, while conventional chemotherapy is strictly limited during this phase. Natural products demonstrate unique advantages during this period: Astragalus has been shown to possess anti‐inflammatory and antifibrotic properties. It inhibits the aggregation and activation of monocytes/macrophages while reducing TGF‐β1 production in the peritoneal cavity [66]. Ginsenoside Rg1 directly influences Th cell activity and Th1/Th2 system development, selectively enhancing glycocalyx transcription product expression, increasing IgA antibody production, and promoting humoral immunity [67]. In a melanoma mouse model, ginsenoside Rh2 induces substantial infiltration of CD4^+^ and CD8^+^ T lymphocytes into tumor tissues, enhancing immune responses [68]. Gegen Qinlian Tablets (GQT), a classical Chinese herbal formula, demonstrates synergistic effects with immune checkpoint inhibitor (ICI) therapy in patients with advanced NSCLC. GQT significantly reduces the incidence of immune‐related adverse events and delays their onset. Regarding antitumor efficacy, GQT also exhibits higher objective response rates and disease control rates [69].

Furthermore, growing research indicates that various natural compounds can improve immune function by altering tight junction structures, microbial metabolites, and gut microbiota composition [70]. For instance, diosgenin can improve gut microbiota composition. After diosgenin administration, the abundance of Sutterella and Lactobacillus species within the Proteobacteria phylum, as well as Bacteroides species within the Bacteroidetes phylum, significantly increased. Tumor tissues exhibited clear IFN‐γ production and CD4/CD8 T cell infiltration [71]. Treatment with icariin significantly increased probiotic bacteria such as Lactobacillus and Bifidobacterium, whose ability to promote antitumor immunity and enhance the activity of various lymphocyte subsets has been demonstrated [72]. Additionally, metabolites like short‐chain fatty acids and indole derivatives increased, exhibiting proven immunomodulatory functions and promoting host metabolic regulation via colonic Gpr41/Gpr43 and AhR signaling pathways [73]. These natural compounds can be integrated with nutritional support and exercise rehabilitation to construct a precise immunonutrition strategy, promoting functional recovery and reducing recurrence risk.

#### 2.2.4 Maintenance and Metastasis Prevention

During the maintenance therapy phase, traditional chemotherapy struggles to achieve long‐term intervention due to its toxic side effects. In contrast, natural products, characterized by low toxicity and suitability for prolonged use, emerge as an ideal choice for maintenance therapy. Research indicates that genistein, the primary active component in soy isoflavones, inhibits tumor metastasis through a multitarget mechanism: on one hand, it downregulates the hypoxia‐inducible factor‐1α (HIF‐1α)/vascular endothelial growth factor (VEGF) signaling pathway to suppress tumor angiogenesis [74]; on the other hand, it modulates the Wnt/β‐catenin pathway to influence the epithelial–mesenchymal transition (EMT) process, thereby reducing tumor cell invasiveness [75]. Concurrently, resveratrol activates the deacetylase SIRT1, promoting metabolic reprogramming in tumor stem cells and inducing their transition toward a differentiated state [76]. Furthermore, it inhibits the activation of the NF‐κB signaling pathway, reducing the release of proinflammatory factors in the TME and altering the microenvironment that favors tumor stem cell maintenance [77]. These natural products with well‐defined molecular mechanisms can achieve sustained “micro‐control” of tumors through standardized dietary supplements, offering novel strategies for long‐term disease management.

#### 2.2.5 Palliative and Adjuvant Therapy

In end‐stage cancer treatment, traditional chemotherapy often becomes unsuitable due to excessive toxicity burdens. Natural products frequently replace chemotherapeutic agents by leveraging their multitarget regulatory properties. Triterpenoids in medicinal Ganoderma lucidum exert comprehensive therapeutic effects through dual mechanisms: centrally, by activating μ‐opioid receptors and modulating the GABA ergic system for analgesic effects; and peripherally, by inhibiting COX‐2 and TNF‐α expression to provide anti‐inflammatory effects. They also improve cachexia‐related anorexia by stimulating ghrelin receptors [78]. The primary active component in Indian frankincense extract, boswellic acid, alleviates anxiety and depression by regulating hypothalamic–pituitary–adrenal axis function, reducing cortisol levels, and enhancing 5‐HT1A receptor activity in the prefrontal cortex [79]. These natural products, with well‐defined molecular mechanisms, not only delay disease progression but also significantly improve patients’ quality of life through multitarget regulation. They provide scientific evidence aligned with holistic medical principles for palliative care, achieving an optimal balance between disease control and quality of life.

## 3 Mechanistic Insights: Targeting Tumor Hallmarks With Natural Products

Natural products exert their anticancer activity by targeting key hallmark features of tumors. These compounds can modulate multiple core biological processes, including apoptosis and cell cycle arrest, angiogenesis and metastasis, and inflammation and oxidative stress [80, 81, 82]. Metabolic alterations in tumor cells play a pivotal role as a central mechanism influencing tumor growth. Natural products can precisely intervene in tumor cell metabolic reprogramming to exert anticancer activity [83].

### 3.1 Apoptosis and Cell Cycle Arrest

Apoptosis is a regulated form of cell death triggered in response to developmental cues or cellular stress. Disruption of apoptotic regulatory mechanisms leads to abnormal proliferation and survival [80]. This selective cell suicide plays a crucial role in numerous physiological and pathological processes, including development, immunity, and disease, where the elimination of damaged or redundant cells helps ensure organismal health [84]. Key apoptotic regulators include Bcl‐2 family proteins (Bcl‐2, Bax) and caspase family proteins (caspase‐3) [85, 86].

The BCL‐2 protein family modulates the activation of intrinsic apoptotic pathways in response to cellular stressors such as DNA damage, γ‐irradiation, oncogene activation, and growth factor withdrawal [87]. Certain natural products can modulate tumor cell apoptosis by influencing BCL‐2 protein levels [88]. Leaf extracts of Vitex doniana, particularly its dichloromethane extract, significantly downregulate the expression of the antiapoptotic gene Bcl‐2 in cervical carcinoma HeLa cells. This pivotal action relieves Bcl‐2's inhibition of the mitochondrial apoptosis pathway, thereby promoting the initiation of intrinsic apoptosis programmes in cancer cells and ultimately effectively suppressing tumour cell proliferation [89]. In DEN‐induced mouse models of hepatocellular carcinoma, leaf extracts from Haloxylon scoparium significantly downregulated the expression of the prosurvival protein Bcl‐2 while simultaneously upregulating levels of the proapoptotic protein BAX. Molecular docking studies confirmed that the flavonoid glycosides abundant in these extracts—such as quercetin and isorhamnetin glycosides—exhibit high‐affinity binding to targets including BCL‐2 [90]. Celastrol has been demonstrated to significantly promote apoptosis in MDA‐MB‐231 cells. Its mechanism of action involves directly targeting and binding to HSDL2, inhibiting its expression, which subsequently downregulates the level of the Bcl‐2 and upregulates the expression of Bax. Ultimately, this process induces apoptosis by suppressing the HSDL2/mitogen‐activated protein kinase (MAPK)/ERK signaling pathway [91].

As a member of the protease family, caspase is a key executor of cell death, participating in both the initiation and execution phases of apoptosis. Numerous pathological processes have been found to correlate with altered caspase activity or variations in the gene expression levels of these enzymes across different cancer types [92]. Among them, caspase‐3 plays a particularly crucial role. As a key protease in the clear apoptosis pathway, its absence or downregulation is associated with carcinogenic effects, indicating its potential as a biomarker for cancer prevention and treatment [93]. Research indicates that Moringa extract significantly inhibits EL4 lymphoma cell growth at a concentration of 100 µg/mL [94]. Tobacco leaf extract effectively suppresses the prosurvival EGFR/PI3K/AKT signaling pathway while activating the TP53‐mediated apoptotic cascade. This mechanism ultimately leads to caspase‐3 cleavage, thereby inducing cell apoptosis [95].

Meanwhile, mutations in cell cycle regulatory genes play a significant role in tumorigenesis. Under normal circumstances, DNA damage triggers cell cycle arrest at checkpoints, providing time for repair to minimize mutations and prevent tumor formation [96]. Zedoary turmeric oil exerts anti‐HCC effects primarily by modulating the EGFR/p53/Bcl‐2 signaling axis, inhibiting cell proliferation and migration, inducing apoptosis, and causing G1 phase cell cycle arrest [97]. The important anticancer natural product—paclitaxel—promotes the assembly of tubulin into microtubules and prevents their depolymerization, thereby further facilitating the formation of stable microtubules. It effectively blocks the G2/M phase of the cell cycle, prevents mitosis, and ultimately inhibits cancer cell proliferation or promotes apoptosis [98]. Podophyllotoxin, an aryltetralin‐type lignan isolated from Podophyllum plants, is widely used in the treatment of various cancers. Similar to colchicine, podophyllotoxin binds to the colchicine site at the α‐ and β‐tubulin interface, inhibits tubulin assembly into microtubules, blocks the cell cycle at the G2/M phase, and ultimately leads to mitotic arrest [99].

### 3.2 Anti‐angiogenesis and Metastasis Suppression

Angiogenesis refers to the process by which tumor cells obtain increased nutrient and oxygen supply through the formation of new blood vessels, which also facilitates their metastasis to other sites via the circulatory system. Therefore, interventions targeting tumor angiogenesis have become a key strategy in cancer treatment [81].

Natural products exert their antiangiogenic effects primarily by inhibiting VEGF and NF‐κB expression, as well as suppressing vascular endothelial cell (EC) growth. The flavonoid natural product naringenin possesses certain antiangiogenic properties. Research indicates that naringenin first reduces interactions among angiogenic factors by inhibiting the secretion of inflammatory cytokines such as IL‐6 and MCP‐1. It then directly suppresses the tyrosine kinase activity of the VEGFR, thereby inhibiting the phosphorylation of related cytokines like FAK and Akt in downstream signaling cascades. and downregulates estrogen receptor alpha to inhibit VEGF production, thereby mediating its antiangiogenic effects [100]. Additional studies indicate that treatment of prostate cancer cells with Tripterygium wilfordii homospermeolide significantly reduces VEGF and COX‐2 expression, inhibiting angiogenesis while enhancing autophagy signaling intensity in PC tissues [101]. Furthermore, dihydroartemisinin(DHA) exhibits potent antiangiogenic activity by blocking NF‐κB p65 nuclear translocation and specifically downregulating VEGF‐2 expression in ECs, thereby demonstrating antiangiogenic properties. Consequently, DHA represents an ideal candidate for use as an angiogenesis inhibitor in tumor therapy [102].

Cancer metastasis is a major cause of cancer mortality, and it begins with the degradation of extracellular matrix (ECM) proteins, enabling tumor cells to enter capillaries or lymphatic vessels and infiltrate various tissues throughout the body [103]. Proteases that promote ECM degradation, such as matrix metalloproteinases (MMPs), are considered enzymes that facilitate metastasis [104]. Compounds from flavonoids, isoflavonoids, cannabinoids, chalcones, naphthoquinones, terpenoids, alkaloids, steroids, and saponins have been identified to inhibit tumor invasion and metastasis. The antimetastatic mechanisms of many natural compounds are found to be associated with MMP inhibition [105]. Studies indicate that evodiamine significantly inhibits the invasion of two nasopharyngeal carcinoma cell lines (HONE1 and CNE1) while only mildly affecting cell proliferation. Evodiamine treatment markedly reduces MMP‐2 mRNA and protein levels, potentially through reduced translocation of nuclear factor‐κB p65, but does not affect MMP‐9 expression levels [106]. According to extensive literature, the anticancer effects of baicalin may be related to inhibiting cell metastasis and invasion [107, 108]. Dose‐dependent anti‐invasive activity of baicalin was observed in OC2 human oral cancer cells and MDA‐MB‐231 human breast cancer cells, accompanied by downregulation of MMP‐2, MMP‐9, and uPA [108, 109].

### 3.3 Targeting Inflammation and Oxidative Stress

Inflammation is one of the primary determinants of cancer, and inflammatory responses are also key characteristics of cancer. Inflammatory cells—including macrophages, dendritic cells (DCs), and neutrophils—are vital components of the TME. Cancer cells can also release large quantities of cytokines and chemokines, which summon immune cells and further exacerbate inflammation, demonstrating the crucial link between inflammation and cancer [110]. Research indicates that chronic inflammation increases cancer risk [111]. For instance, chronic bronchitis elevates lung cancer risk; chronic pancreatitis can induce pancreatic cancer; and Helicobacter pylori load is a major determinant of gastric cancer [112, 113, 114]. Natural products play a vital role in anti‐inflammatory and antioxidant stress mechanisms, holding significant potential for cancer treatment by modulating inflammatory responses.

Puerarin, extracted from kudzu root, has demonstrated anti‐inflammatory effects across various disease models. Puerarin elevated IL‐2 and superoxide dismutase activity in plasma of U14 cervical carcinoma mice. At specific doses, it eliminated excess free radicals [115]. Reports indicate puerarin mitigated reactive oxygen species (ROS)‐induced tissue damage, subsequently enhancing antitumor efficacy [116]. Luteolin has been found to possess multiple therapeutic effects. It promotes tumor cell apoptosis by inhibiting NF‐κB activation through TNF‐α suppression and enhancing natural killer (NK) cell activity [117]. EGCG is renowned for its diverse pharmacological activities. As a catechin compound, EGCG simultaneously inhibits COX‐2 by suppressing NF‐κB activation in HeLa and SiHa cells while regulating ROS levels, suggesting its antitumor effects may operate through antioxidant pathways [118]. In line with this mechanism, extracts from the traditional medicinal plant *Rhus punjabensis* Stewart and its isolated major triterpenoid compounds (such as lupeol) significantly inhibit the NF‐κB inflammatory signaling pathway, effectively scavenge free radicals (as confirmed by the DPPH assay), and induce apoptosis in cancer cells, demonstrating remarkable anticancer potential [119].

### 3.4 Metabolic Reprogramming as a Key Step in Natural Product‐Based Cancer Therapy

**FIGURE 2:**
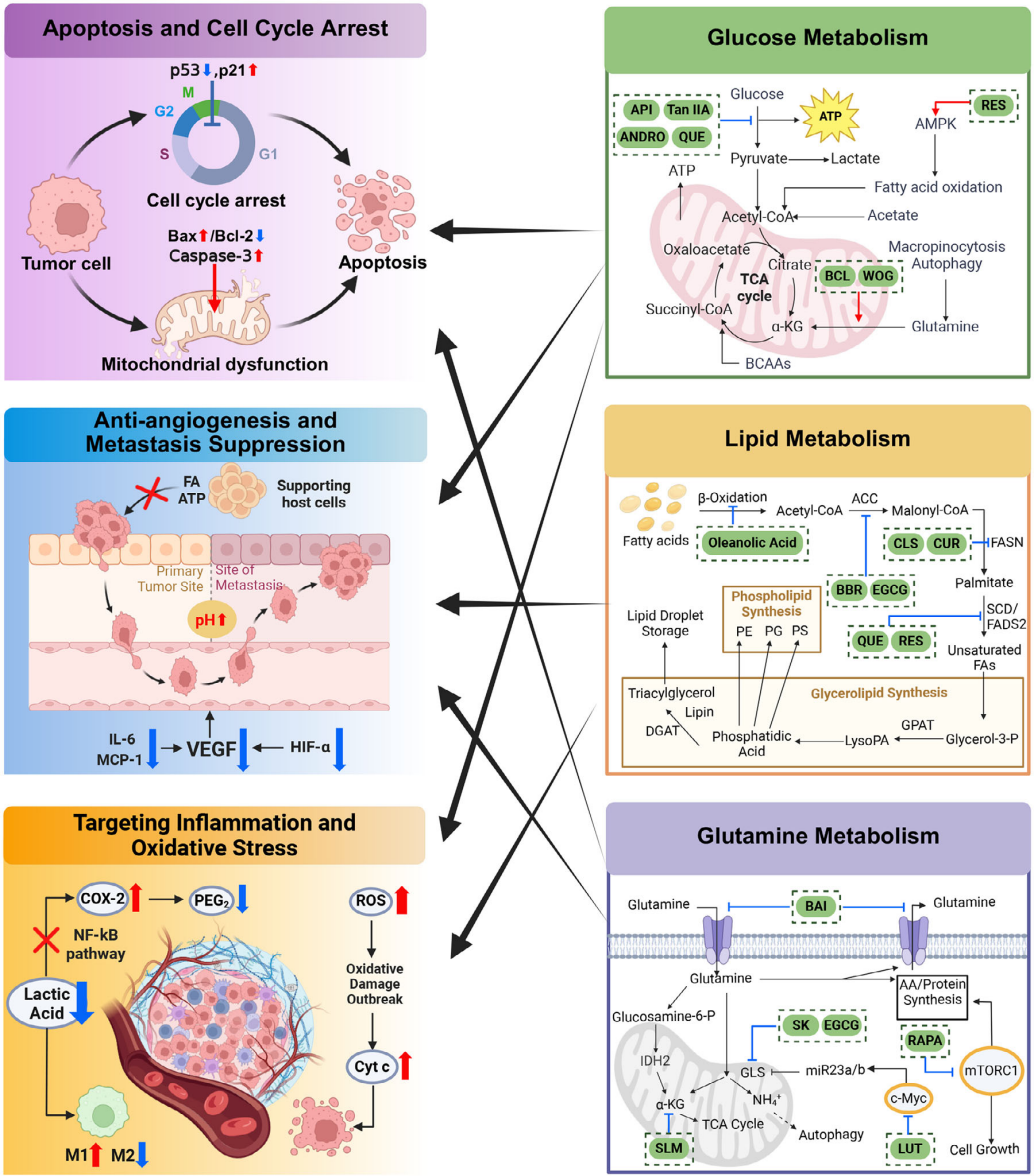
Multitarget antitumor mechanisms of natural products: from metabolic reprogramming to tumor hallmarks. *Abbreviations*: API: apigenin, Tan IIA: tanshinone IIA, ANDRO: andrographolide, QUE: quercetin, RES: resveratrol, BCL: baicalin, WOG: wogonin, CLS: celastrol, CUR: curcumin, BBR: berberine, EGCG: epigallocatechin‐3‐gallate, BAI: baicalein, SK: shikonin, RAPA: rapamycin, SLM: silymarin, LUT: luteolin.

Metabolic reprogramming serves as the core driver of natural product‐targeted tumor therapy mechanisms. The defining characteristics of cancer cells lie not only in their uncontrolled proliferation but also in their unique energy metabolism patterns [120]. The classic “Warburg effect” reveals that even under oxygen‐rich conditions, cancer cells tend to undergo high‐rate glycolysis. This phenomenon signifies that metabolic reprogramming has become one of the core hallmarks of cancer, enabling tumor cells to sustain rapid proliferation, resist cell death, and adapt to microenvironmental stress [121]. Consequently, targeting these abnormal metabolic pathways has emerged as a highly promising anticancer strategy. Structurally diverse, multitargeted natural products can exert their anticancer effects by precisely intervening in key metabolic processes of cancer cells [83]. Figure 2 integrates the multitarget mechanisms of natural products’ anticancer effects.

Regarding glucose metabolism, cancer cells heavily rely on aerobic glycolysis, and natural products can effectively inhibit key nodes in this process. For instance, berberine has been demonstrated to downregulate glucose transporter 1 (GLUT1) expression and inhibit hexokinase 2 (HK2) activity [122]. Concurrently, flavonoids like quercetin interfere with pyruvate kinase M2 (PKM2) function, effectively suppressing glycolytic flux [123]. Ferulic acid reduces GLUT1 expression and glucose uptake in cancer cells, while silymarin competitively inhibits GLUT4, offering pathways to restrict nutrient supply and inhibit tumor growth [124, 125]. Regarding lipid metabolism, cancer cells enhance lipid synthesis to meet the rapid demands of cell membrane construction. Among dietary flavonoids, EGCG from green tea exhibits significant fatty acid synthase (FASN) inhibitory activity, primarily through its galloyl group specifically binding to the ketone reductase domain of FASN. Despite EGCG's multifaceted effects on tumor cells, its FASN‐targeting properties make it a highly promising lead compound for chemoprevention and therapy [126]. Additionally, natural products play crucial roles in glutamine metabolism. Glutaminase 1 (GLS1), a key enzyme in glutamine degradation, can be downregulated by compounds like CUR and quercetin. This deprives cancer cells of essential nitrogen and carbon sources, thereby inhibiting their proliferation [127, 128]. From alkaloids to polyphenols, diverse classes of natural products effectively reverse cancer cell metabolic reprogramming through multitargeted, multilevel mechanisms.

## 4 The Role of Flavonoids in Cancer Metabolic Reprogramming

Flavonoids, as important natural products, exert significant anticancer effects by targeting tumor metabolic reprogramming processes. These compounds can systematically regulate core pathways such as glucose metabolism, lipid metabolism, and glutamine metabolism, thereby influencing energy supply and biosynthesis in tumor cells [129]. As multitarget metabolic modulators, they hold significant application potential in cancer therapy.

### 4.1 Structural Types and Sources

As natural compounds, flavonoids are representative compounds that exert antitumor effects by regulating abnormal metabolic networks. By targeting key metabolic pathways—including glycolysis, lipid synthesis, glutamine utilization, and nucleotide metabolism—flavonoids disrupt both the energy supply and biosynthetic demands essential for tumor survival and progression [129]. Uniquely, flavonoids can simultaneously influence cancer cell energy metabolism, redox homeostasis, and intracellular signaling cascades, underscoring their versatility as metabolic regulators in cancer therapy [130].

Structurally, over 4000 flavonoid compounds have been identified in plants. All flavonoids share a core backbone composed of two aromatic benzene rings (A and B) connected by a three‐carbon bridge forming a heterocyclic pyran ring (C), commonly described as the C6–C3–C6 skeleton [33]. Variations in hydroxylation, methoxylation, glycosylation, and prenylation patterns give rise to multiple subclasses, including flavonols, flavones, isoflavones, flavanones, flavanols (catechins), chalcones, anthocyanins, and flavanonols [131]. This structural diversity is closely linked to their biological activities and influences their capacity to target various aspects of tumor metabolism.

Different flavonoid subclasses exert distinct effects on cancer‐related metabolic pathways. For instance, flavanols such as EGCG modulate receptor tyrosine kinases, lipid rafts, fatty acid metabolism, and epigenetic regulators, and also target proteasomes, telomerase, and cancer stem cells [132]. Isoprenylated flavonoids and chalcones influence carcinogen metabolism by inhibiting phase I metabolizing enzymes and activating phase II detoxification systems, while also exerting anti‐inflammatory and antiproliferative effects [133]. Flavonols such as kaempferol demonstrate antiproliferative activity in lung and endothelial cancer cells through MAPK signaling modulation [134]. Polymethoxyflavones derived from citrus peels have been shown to inhibit ovarian cancer cell growth by downregulating Akt, HIF‐1α, NF‐κB, and VEGF, thereby impairing angiogenesis [135].

Anthocyanins also play key roles in metabolic regulation. These pigments suppress cancer cell migration and reverse EMT by inactivating PI3K/Akt signaling in hepatocellular carcinoma cells [136]. Certain anthocyanin derivatives, such as geranylgeranyl anthocyanins, induce G2/M cell cycle arrest, mitochondrial dysfunction, and ROS‐mediated autophagy, while inhibiting p‐PI3K and p‐Akt in a dose‐dependent manner [137].

Together, the structural diversity and functional specificity of flavonoids enable them to act on multiple metabolic and signaling axes in cancer cells. These properties make flavonoids a promising class of natural compounds for innovative anticancer strategies. Their ability to selectively target tumor metabolism while maintaining low systemic toxicity offers considerable therapeutic potential for both prevention and adjuvant cancer treatment [138].

### 4.2 Mechanism of Action: Glucose, Lipid, and Glutamine Metabolism

Flavonoids play a crucial role in inhibiting tumor growth by interfering with the metabolic reprogramming of cancer cells. Their regulatory effects are primarily manifested in three core pathways: glucose metabolism, lipid metabolism, and glutamine metabolism [129].

#### 4.2.1 Inhibition of Glucose Metabolism

**FIGURE 3:**
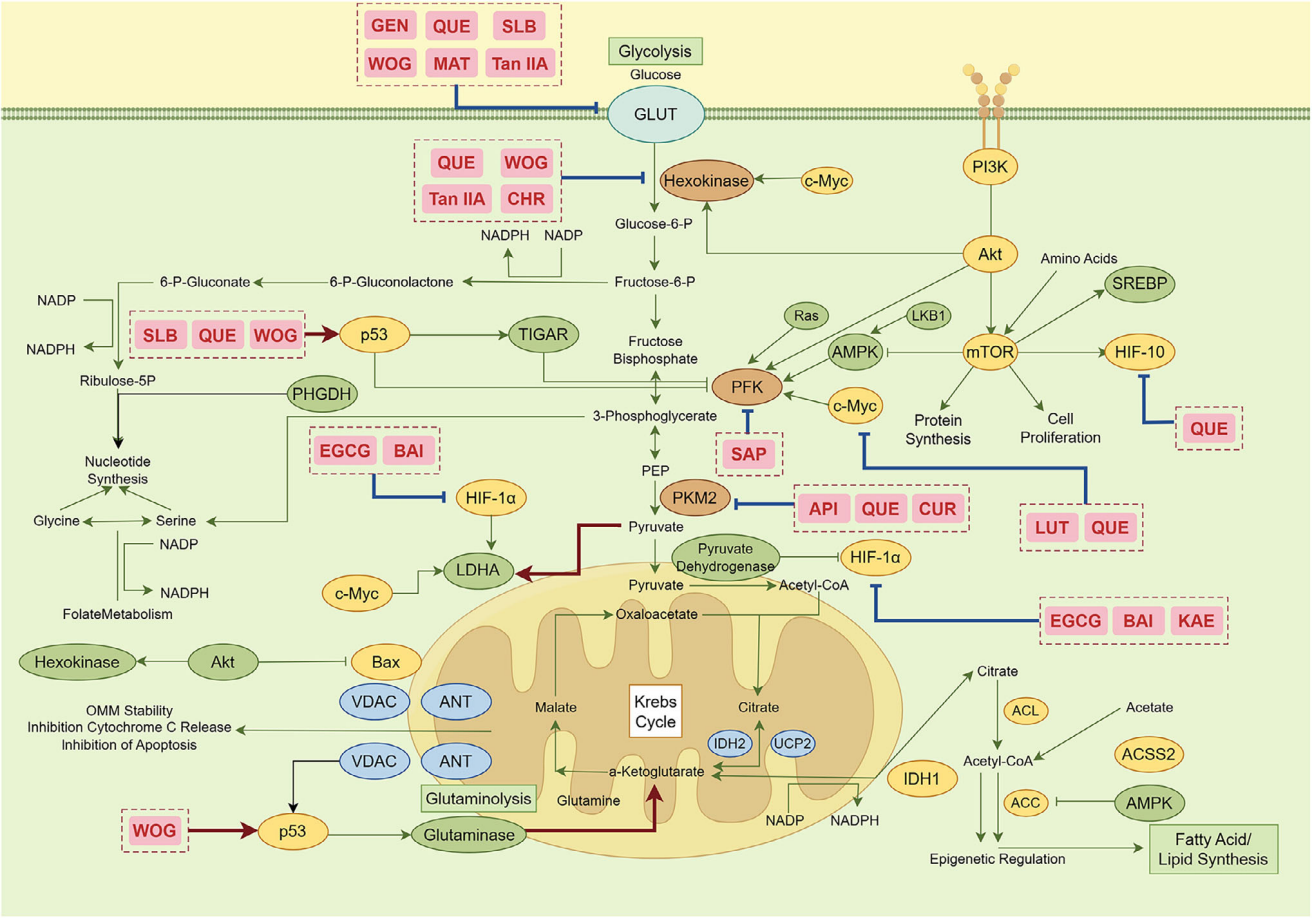
Central regulatory network of flavonoids targeting glucose‐driven metabolic reprogramming in cancer cells. *Abbreviations*: GEN: genistein, QUE: quercetin, SLB: silybin, MAT: matrine, WOG: wogonin, Tan IIA: tanshinone IIA, chrysin: CHR, EGCG: epigallocatechin‐3‐gallate, BAI: baicalein, SAP: sapogenin, API: apigenin, CUR: curcumin, LUT: luteolin, KAE: kaempferol.

The aerobic glycolysis characteristic of cancer cells is a hallmark of their metabolic reprogramming. This metabolic shift enables cancer cells to rapidly generate ATP while acquiring abundant biosynthetic precursors to meet their proliferative demands [139]. Figure 3 demonstrates that flavonoids effectively reverse this metabolic abnormality through multitargeted action.

At the molecular mechanism level, apigenin significantly downregulates the expression and activity of PKM2 by inhibiting the Akt/mTOR signaling pathway. As a key regulatory enzyme in glycolysis, reduced PKM2 activity directly leads to decreased glycolytic flux, triggering ATP depletion and NADH/NAD^+^ imbalance. This downregulates antiapoptotic proteins such as Mcl‐1, thereby promoting mitochondrial apoptosis [140]. Concurrently, ferulic acid reduces glucose transporter GLUT1 expression, curtailing glucose uptake at its source [124]. Notably, catechin inhibits lactate dehydrogenase A (LDHA), blocking pyruvate conversion to lactate. This not only elevates intracellular pH but also triggers substantial ROS accumulation, ultimately inducing apoptosis [141].

Furthermore, flavonoids exert global regulation of glycolysis by modulating key transcription factors. For instance, quercetin and EGCG activate the AMPK pathway to inhibit the stability and transcriptional activity of HIF‐1α [142, 143]. This simultaneously impairs glycolysis while inducing cell cycle arrest and senescence. Meanwhile, flavonoids like  Oroxylin A stabilize the p53 protein, enhancing its suppression of glycolysis‐related genes [144]. Collectively, these actions substantially diminish cancer cells' glycolytic capacity, reshaping their energy metabolism patterns.

#### 4.2.2 Regulate Lipid Metabolism

Lipid metabolism reprogramming is another key feature enabling cancer cells to sustain rapid proliferation. By enhancing the synthesis of fatty acids, cholesterol, and phospholipids, cancer cells provide the material foundation for cell membrane biogenesis, signaling, and energy storage [145]. Flavonoids exhibit multifaceted regulatory roles in this process.

Regarding fatty acid metabolism, EGCG specifically binds to the ketone acyl reductase domain of FASN via its unique galloyl group, effectively inhibiting the enzyme's catalytic activity. As the key enzyme for de novo fatty acid synthesis, FASN inhibition directly impedes fatty acid production [126]. Studies indicate that flavonoids such as quercetin, kaempferol, and luteolin exhibit similar FASN inhibitory activity, suggesting this may represent a common mechanism of action for flavonoids [146, 147, 148]. As the key rate‐limiting enzyme in de novo fatty acid synthesis, inhibition of FASN directly blocks fatty acid supply, leading to impaired membrane lipid synthesis and disruption of lipid rafts. This induces endoplasmic reticulum stress and activates the mitochondrial apoptosis pathway [149].

In cholesterol metabolism regulation, flavonoids modulate the transcription of cholesterol synthesis‐related genes by influencing the activity and expression of SREBPs. Quercetin and EGCG interfere with SREBP cleavage and nuclear translocation, thereby suppressing the expression of key enzymes like 3‐hydroxy‐3‐methylglutaryl‐coenzyme A reductase (HMGCR)[150]. Additionally, these compounds activate liver X receptor, promoting cholesterol reverse transport and further maintaining cholesterol homeostasis.

At the phospholipid metabolism level, resveratrol reduces arachidonic acid release and subsequent inflammatory mediator production by inhibiting PLA2 activity [151]. This effect blocks prosurvival signaling pathways and reduces levels of antiapoptotic factors such as PGE_2_, thereby sensitizing cells to death signals like TNF‐α [152]. Simultaneously, quercetin interferes with phosphatidylcholine synthesis by suppressing choline kinase activity, directly affecting cell membrane biosynthesis and associated signaling pathways [153]. This blocks cell membrane biosynthesis and disrupts lipid second messenger balance, leading to cell cycle arrest in the S phase [154].

#### 4.2.3 Disrupt Glutamine Metabolism

Glutamine serves as a crucial carbon and nitrogen source for cancer cells, playing a vital role in sustaining their proliferation [155]. Flavonoids effectively disrupt the metabolic balance of cancer cells by interfering with different stages of glutamine metabolism.

Silymarin specifically binds to isocitrate dehydrogenase 1 (IDH1), inhibiting its catalytic activity. IDH1 catalyzes the oxidative decarboxylation of isocitrate to form α‐ketoglutarate (α‐KG), forming a critical link between glutamine metabolism and the tricarboxylic acid cycle. Inhibiting IDH1 activity alters the NADP^+^/NADPH ratio, elevates oxidative stress levels, and ultimately suppresses tumor cell proliferation [156].

**FIGURE 4:**
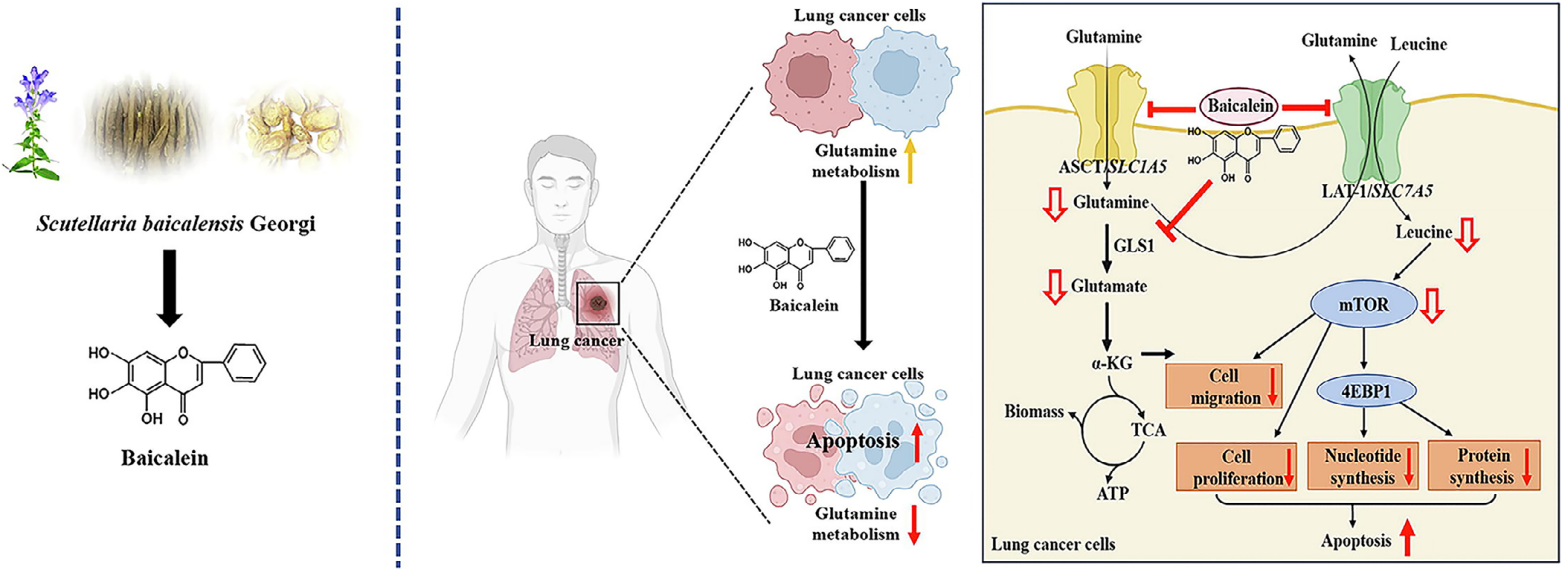
Baicalein derived from Scutellaria baicalensis inhibits glutamine uptake and mTOR signaling, leading to metabolic disruption and apoptosis in lung cancer cells. Reproduced with permission [157]. Published by Elsevier Inc.

Notably, baicalin achieves dual inhibition of glutamine metabolism by suppressing the mTOR signaling pathway and downregulating glutamine transporter expression. In lung cancer models, baicalin treatment significantly reduced intracellular glutamine utilization and inhibited tumor growth (Figure 4) [157]. This multitargeted action deprives tumor cells of their synthetic precursors and energy sources, inducing autophagic cell death by activating the AMPK/p53 axis.

In addition to targeting enzymatic regulators, flavonoids also interfere with glutamine uptake and downstream catabolism. Flavokawain A, a chalcone derived from Piper methysticum (kava), significantly reduces intracellular levels of glutamine, glutamate, and proline in prostate cancer cells. This metabolic suppression leads to a reduction in glutathione (GSH) synthesis, triggering oxidative stress and inducing apoptosis via ROS accumulation [158].

## 5 Metabolic Reprogramming of Other Categories of Natural Products and Their Anticancer Activity

Natural products play a crucial role in modulating the metabolic reprogramming of cancer cells. These compounds regulate key processes such as tumor cell energy metabolism, biosynthesis, and redox balance, contributing to their anticancer activity [159]. A comprehensive overview of how various natural products influence tumor metabolism is summarized in Table 2 [127, 160, 161, 162, 163, 164, 165, 166, 167, 168, 169, 170, 171, 172, 173, 174, 175, 176, 177, 178, 179, 180, 181, 182, 183, 184, 185, 186, 187, 188], highlighting the diverse mechanisms through which they exert their therapeutic effects. In addition to flavonoids, a wide range of structurally diverse natural products also target metabolic pathways to interfere with cancer cell reprogramming, offering unique mechanisms of action for cancer therapy [83].

**TABLE 2:** Mechanisms of action of natural products in regulating tumor metabolism.

Metabolism type	Key target	Representative natural products	Mechanism of action	Cancer type/cell model	Effects	References
Glucose metabolism	PKM2	Quercetin, apigenin	PKM2 expression via Akt/mTOR or β‐catenin/c‐MYC/PTBP2↓	Colorectal cancer and breast cancer cells	Glycolysis↓, apoptosis↑	[161, 162]
	HK2	Quercetin, xanthohumol	HK2 via Akt/mTOR or EGFR–Akt signaling↓	HCC, glioblastoma	Glucose phosphorylation and ATP production↓	[160, 163]
	PFK1	Amentoflavone	PFKP expression↓, glycolysis inhibition	HepG2	Glycolytic flux↓	[164]
	GAPDH	Luteolin	Competitive inhibition of GAPDH enzymatic activity	HepG2, HeLa	Glycolytic flux↓, cell cycle arrest	[165]
	ENO1	Silibinin	mRNA levels of ENO1 gene↓, disrupting glycolysis	Breast cancer	ATP↓, chemosensitivity↑	[166]
	GLUT1/GLUT4	Apigenin, casticin	Glucose uptake via transporter suppression↓	Multiple solid tumors	Glucose availability↓, cell proliferation↓	[167, 168]
	SGLT1/2	Trilobatin	Nonselective inhibition of SGLT‐mediated uptake	Human hepatoblastoma	Glucose uptake↓, tumor suppression	[169]
	PGK1	Isoflavones	miR‐29a/miR‐1256‐mediated PGK1 inhibition	Prostate cancer	↓Migration, invasion	[170]
	LDHA	Catechins	LDHA↓ → ROS↑, apoptosis induction	Gastric cancer	Induce ROS‐mediated cytotoxicity	[171]
	HIF‐1α	EGCG, baicalein, kaempferol	HIF‐1α expression/activity via AMPK or direct inhibition↓	HeLa, HepG2	Angiogenesis↓, glycolysis↓, apoptosis↑	[172, 173, 174]
	p53	Oroxylin A, baicalein	p53 stability↑, expression → GLUTs↓, apoptosis↑	HepG2	Tumor growth↓, glycolysis suppression↑	[175, 176]
	c‐MYC	Lignans	c‐MYC signaling↓ (PI3K/Akt, Src/Cortactin, EMT‐related)	Breast, lung cancer	Proliferation↓, metastasis↓, glycolysis↓	[177]
	NF‐κB	Epicatechin	NF‐κB nuclear translocation↓	Multiple solid tumors	Glycolysis‐related gene expression↓	[178]
	miR‐214‐5p	Cyanidin‐3‐O‐glucoside (C3G)	miRNA‐mediated regulation of GLUT1 and AKT/mTOR/PTEN pathway	Oral squamous carcinoma, others	Metabolic inhibition, cell growth↓	[179]
	AMPK, Bax/Bcl‐2	Multiple flavonoids	AMPK activation, apoptosis modulation	Various cancer types	Mitochondrial dysfunction, viability↓	[180, 181]
Lipid metabolism	FASN	EGCG	KR domain activity of FASN↓, blocks fatty acid synthesis	Various solid tumors	Tumor growth↓	[182]
	Acetyl‐CoA carboxylase (ACC)	Fisetin	ACC activity↓, malonyl‐CoA production↓	Prostate cancer	Fatty acid synthesis↓, apoptosis↑	[183]
	Stearoyl‐CoA desaturase‐1 (SCD1)	Genistein	SCD1 expression via AMPK activation↓	Lung cancer	Disrupts membrane fluidity, metastasis↓	[184]
	NRF2	Hesperetin	Activates NRF2 antioxidant pathway, indirectly regulates cholesterol homeostasis	HepG2	Stress resistance↑, delays tumor progression	[185]
	HMG‐CoA reductase (HMGCR)	Naringenin	Competitive inhibition of HMGCR catalytic site	Liver cancer	Cholesterol synthesis blocks the mevalonate pathway↓	[186]
	PLA2	Resveratrol	PLA2 activity↓, production of proinflammatory lipid mediators↓	Breast cancer	Dual antitumor and anti‐inflammatory effects	[187]
Glutamine metabolism	Glutaminase (GLS)	Curcumin	Glutaminase activity↓, glutamine utilization↓	Adrenocortical carcinoma	Deprives cancer cells of nitrogen and carbon sources, inhibits proliferation	[127]
	Glutamine	Pholiota adiposa ethanol extract	Levels of amino acids such as glutamine↓, the compensatory energy supply from branched‐chain amino acids↓	Liver cancer	Tumor growth↓, tumor cell apoptosis↑, protect liver and kidney function, and regulate immune responses	[188]

### 5.1 Alkaloids

Alkaloid compounds exhibit significant activity in regulating tumor metabolism [189]. Berberine, as a representative of isoquinoline alkaloids, effectively disrupts glucose metabolism and lipid synthesis in tumor cells by inhibiting mitochondrial complex I function and activating the AMPK signaling pathway, thereby inducing metabolic stress, and this energy and synthetic impairment ultimately triggers oxidative stress and mitochondrial dysfunction, leading to cancer cell apoptosis [190]. Research confirms that physapubescin modulates glutamine metabolism by inhibiting kidney‐type glutaminase activity, leading to reduced intracellular α‐KG levels and exerting antitumor effects [191].

### 5.2 Terpenoids

Terpenoids exert multitarget regulatory effects in metabolic reprogramming. DHA significantly reduces the viability of JF‐305 cells, arrests the cell cycle at the G2/M phase, and induces apoptosis by diminishing mitochondrial membrane potential and ROS accumulation. Concurrently, it suppresses tumor cell glucose uptake, lactate production, and ATP generation, while also downregulating the Akt/mTOR signaling pathway and the expression of GLUT1 [192]. Triptolide, a primary active compound isolated from *Tripterygium wilfordii* Hook. F., exhibits significant anticancer potential by promoting cell death and autophagy in lung and prostate cancer cells through the activation of CaMKKβ–AMPK signaling and modulation of the AMPK/mTORC2 axis [193].

### 5.3 Polyphenols

Other polyphenolic compounds demonstrate unique advantages in regulating tumor metabolic reprogramming. CUR not only inhibits FASN and ATP citrate lyase, but also reduces GLUT1 and HK2 expression by modulating c‐Myc [194, 195]. These effects synergistically impair cancer cells’ lipid synthesis and glycolytic capacity, leading to membrane structural damage and energy depletion, which in turn trigger apoptosis and cell cycle arrest. Green tea polyphenol EGCG inhibits glucose transporter and lactate dehydrogenase activity, while ellagic acid interferes with the expression of key enzymes in glutamine metabolism [196, 197]. Furthermore, studies indicate that water extracts of Chamerion angustifolium L. and its primary polyphenolic component oenothein B can inhibit colon cancer cells by targeting mitochondrial energy metabolism. Both agents reduce Caco‐2 cell viability in a dose‐dependent manner and significantly suppress oxidative phosphorylation function in mitochondrial complexes I and II [198]. These polyphenolic compounds effectively disrupt tumor cell metabolic networks through multitarget synergistic actions.

### 5.4 Sulfur Compounds

Sulfur‐containing natural products play a significant role in metabolic regulation due to their unique mechanisms of action. Allicin specifically inhibits HK2 and PKM2 activity while influencing lipid synthesis by activating the AMPK pathway [199]. Sulforaphane not only enhances antioxidant capacity through the Nrf2 pathway but also promotes mitochondrial metabolism by suppressing PDK activity. By inhibiting PDK to promote mitochondrial oxidative phosphorylation, sulforaphane may induce metabolic imbalance while enhancing oxidation, ultimately eliminating cancer cells through activation of apoptotic pathways or ferroptosis [200]. Furthermore, glucosinolate metabolites in broccoli inhibit histone deacetylases, affecting metabolic gene expression; while sulfur compounds in onions disrupt GSH metabolism, amplifying oxidative stress [201, 202]. These sulfur‐containing compounds offer unique targets for modulating tumor metabolism.

### 5.5 Synergistic Metabolic Regulation Strategy

The combined use of different categories of natural products can produce synergistic metabolic regulatory effects. Traditional Chinese medicine formulas, as typical representatives of multicomponent natural products, demonstrate unique advantages in synergistically regulating tumor metabolism. For example, Huangqi Fuling Decoction (HQFLD) synergistically targets the RAS–MEK–ERK signaling pathway through its multiple active components (such as astragaloside IV and astragaloside V) to inhibit gastric cancer cell proliferation and metastasis. It also downregulates key metabolism‐related genes including VCAM1, ICAM1, PTGS2, thereby disrupting tumor energy metabolism and cell adhesion processes [203]. This multitargeted intervention collectively impairs the tumor's energy supply, invasive capacity, and inflammatory microenvironment, ultimately enhancing tumor growth suppression through metabolic collapse and enhanced apoptotic signaling.

Another compound LTTL, regulates serum microRNAs (e.g., miR‐2110, miR‐7d‐3p) associated with glucose metabolism pathways (such as glycolysis/gluconeogenesis, fructose, and mannose metabolism). This modulates metabolic reprogramming processes related to lung cancer pain, demonstrating the compound's multitarget synergistic effects within the metabolism–immunity–pain regulatory network. miR‐7d‐3p to influence metabolic reprogramming processes associated with lung cancer pain, demonstrating the multitarget synergistic effects of the compound within the metabolic–immune–pain regulatory network [57].

These studies demonstrate that traditional Chinese medicine formulas, through the synergistic effects of multiple natural products, can simultaneously intervene in several key aspects of tumor metabolism—such as energy metabolism, oxidative stress, and signaling pathway activation—thereby more effectively addressing the heterogeneity and plasticity of tumor metabolism.

## 6 Regulation of the TME by Natural Products

The TME is a complex ecosystem composed of tumor cells, immune cells, stromal cells, vascular networks, and ECM [204]. Through intricate interactions, these components collectively influence cancer progression, immune evasion, and therapeutic resistance [205]. Recent studies have revealed that natural products can effectively modulate various components within the TME via multitarget mechanisms, thereby inhibiting tumor progression [206].

### 6.1 Cancer‐Associated Fibroblasts

CAFs are key effector cells in the tumor stroma that actively participate in ECM remodeling by secreting multiple cytokines and growth factors, thereby promoting tumor growth and metastasis [207]. Studies indicate that Huaier can significantly reduce the expression levels of α‐smooth muscle actin and fibronectin in CAFs by inhibiting the TGF‐β/Smad signaling pathway, thereby suppressing the activation process of CAFs [208]. Tripterygium glycosides can inhibit the TGF‐β signaling pathway in CAFs, reversing their activated state to a quiescent state. This reduces ECM secretion and disrupts their role in supporting tumor growth [209]. Additionally, the traditional Chinese medicine compound Qingre Huayu Jianpi decoction has been demonstrated in colorectal cancer studies to effectively inhibit the activation and migration of CAFs, reduce the deposition of ECM components such as collagen, and suppress the growth of tumor organoids by regulating the Wnt/β‐catenin pathway. This highlights the comprehensive advantages of natural multicomponent drugs in synergistically regulating both tumor cells and CAFs [210].

### 6.2 Endothelial Cells

Tumor‐associated ECs support tumor angiogenesis through unique metabolic reprogramming, a process critical for tumor growth and metastasis [211]. CUR effectively inhibits the phosphorylation of VEGFR2, blocking the activation of downstream angiogenic signaling pathways [212]. Berberine B induces EC apoptosis, specifically disrupting the structural integrity of the tumor vascular network [213]. Notably, the natural product deoxypodophyllotoxin isolated from Anthriscus sylvestris promotes cytoskeletal remodeling in human umbilical vein ECs by activating the AMPK signaling pathway, thereby inhibiting angiogenesis and blocking tumor growth and metastasis [214]. Recent studies indicate that betulin, a triterpenoid compound derived from white birch bark, reduces mRNA stability by inhibiting m6A modification of PAX2, thereby decreasing PAX2 expression. This process dissociates PAX2 from the VEGF‐A promoter, ultimately blocking the VEGF‐A/VEGFR2 signaling pathway. This process inhibits EC migration and tubulogenesis in vitro, reduces tumor microvascular density in vivo, and thereby effectively suppresses tumor growth and metastasis [215]. These natural products stimulate ECs, collectively exerting an inhibitory effect on tumor angiogenesis.

### 6.3 ECM Components

During tumor progression, the ECM undergoes a series of dynamic changes that profoundly influence the physical properties and biochemical characteristics of the TME [216]. Flavonoids including lignans counteract these effects by inhibiting MMP expression through modulation of Notch signaling and specific microRNAs (e.g., miR‐21) [217]. Compounds like hypericin further suppress MMP‐3, MMP‐13, and ADAMTS5 expression in response to TNF‐α, thereby preserving ECM integrity and restricting tumor cell access to metabolic resources [218]. Collectively, these actions maintain the structural and functional integrity of the ECM, creating favorable conditions for suppressing tumor invasion and metastasis.

### 6.4 Diverse Tumor‐Infiltrating Immune Cells

Natural products exert extensive regulatory effects on immune cells within the TME [219]. Icariin promotes the maturation process of DCs, enhancing their antigen‐presenting function [220]. Arctigenin activates the Toll‐like receptor signaling pathway in macrophages, enhancing their phagocytic and cytotoxic capabilities [221]. The traditional Chinese medicine compound Jianpi‐Huayu Decoction has been shown to enhance NK cell cytotoxicity by suppressing TREM1/DAPI2 signaling in TAMs, thereby alleviating immunosuppression in the TME and synergizing with PD‐1 inhibitors in hepatocellular carcinoma [222]. Tetrandrine, derived from *Stephania tetrandra* S. Moore, is a bisbenzylisoquinoline alkaloid with the ability to activate the STING/TBK1/IRF3 pathway, promoting CCL5 and CXCL10 production. This enhances the infiltration of macrophages, DCs, and CD8 T cells in the TME, significantly inhibiting the growth of NSCLC [223]. Collectively, these immunomodulatory effects amplify the body's antitumor immune response, offering novel insights for tumor immunotherapy.

## 7 The Regulatory Effects of Natural Products on the Immune System

**FIGURE 5:**
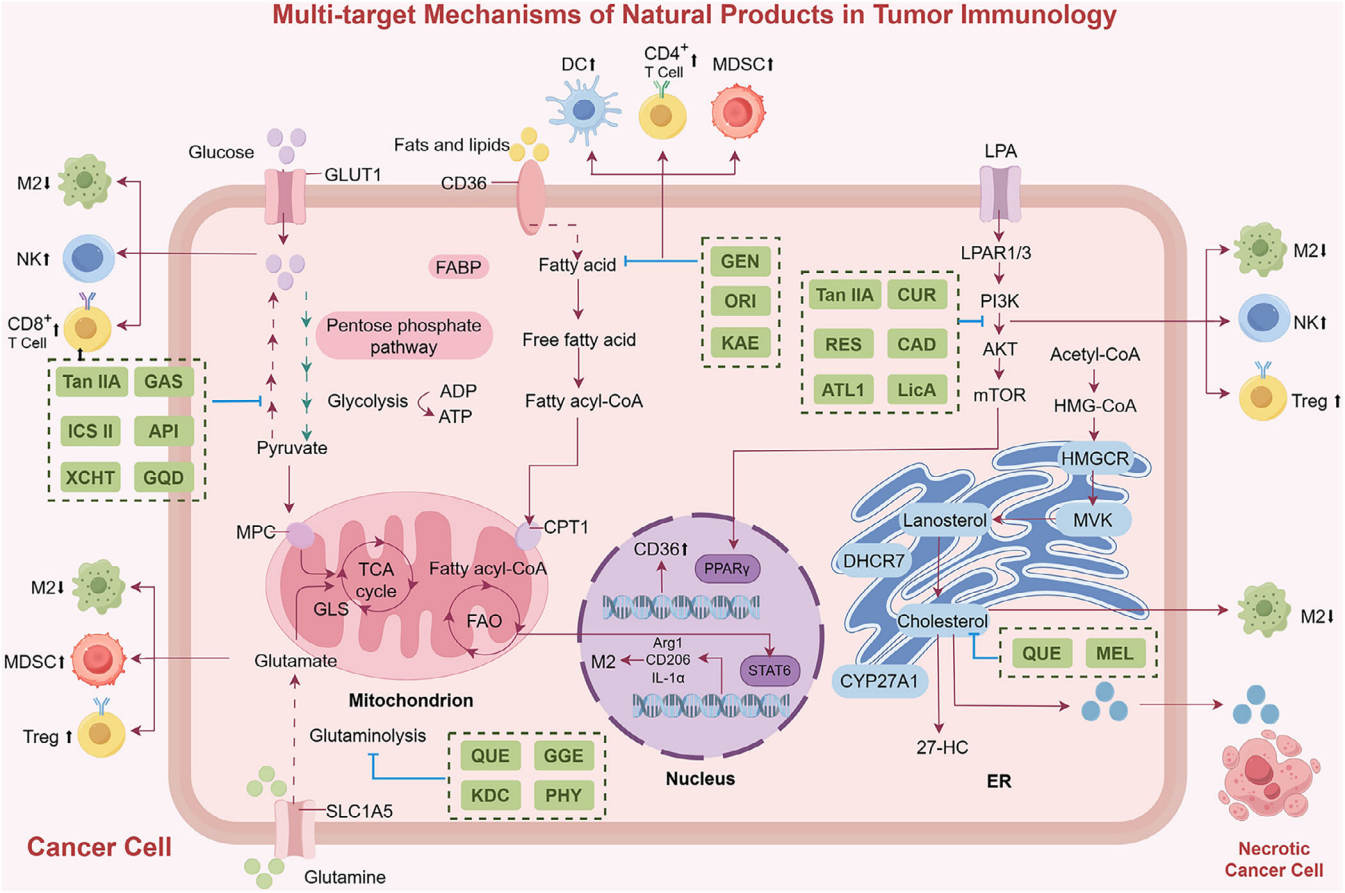
Multitarget mechanisms of natural products in tumor immunology. *Abbreviations*: Tan IIA: tanshinone IIA, GAS: gastrodin, ICS II: icariside II, API: apigenin, XCHT: Xiao Chai Hu Tang, QUE: quercetin, GGE: Glycyrrhiza glabra extract, KDC: Llex tarajois (Kudingcha), PHY: physapubescin I, GEN: genistein, ORI: oridonin, KAE: kaempferol, CUR: curcumin, RES: resveratrol, CAD: cardamonin, ATL1: atractylenolide I, LicA: licochalcone A, MEL: melitidin.

Natural products modulate tumor immune responses through multiple pathways and targets, not only directly enhancing immune cell function but also effectively reversing immune suppression in the TME, thereby strengthening the body's antitumor immune capacity [224] (Figure 5).

### 7.1 Tumor‐Associated Macrophages

Ginsenoside Rb1 promotes macrophage polarization toward the M1 phenotype with antitumor functions by regulating the NF‐κB signaling pathway, while simultaneously enhancing phagocytic activity [225]. Soy isoflavones, conversely, inhibit the polarization of macrophages toward the tumor‐promoting M2 phenotype, reducing the secretion of immunosuppressive factors [226]. Notably, resveratrol influences the metabolic reprogramming of macrophages by modulating the AMPK/mTOR signaling pathway [227]. In the context of STAT pathway‐mediated M2 polarization driving an aggressive tumor phenotype, Garcinone E blocks the M2 polarization process by inhibiting STAT6 phosphorylation, while Mongolian Iris Extract specifically targets the IL‐10/STAT3/PD‐L1 axis to reverse M2‐mediated immune evasion [228, 229]. Collectively, these actions reverse macrophage‐mediated immunosuppression, creating favorable conditions for improving the TME.

### 7.2 Dendritic Cells

Berberine promotes DC maturation and antigen presentation by activating the AMPK signaling pathway [230]. Research indicates that quercetin improves the immune function of DCs by influencing glucose metabolism [231]. These studies hold significant reference value for the development of whole‐cell nanovaccines targeting DCs. The GGT nanovaccine carrier, based on the self‐assembly of chitin oligosaccharides and Ganoderma lucidum polysaccharides, activates systemic antitumor immunity across multiple tumor models by efficiently delivering antigens to DCs and promoting their maturation [232]. Collectively, these effects amplify the pivotal role of DCs in antitumor immune responses.

### 7.3 NK Cells

Apigenin enhances NK cells’ ability to recognize and kill tumor cells by regulating the expression of surface activation receptors [233]. EGCG promotes NK cell production of perforin and granzyme, thereby amplifying their cytotoxic effects [234]. Furthermore, allicin improves mitochondrial function, thereby enhancing NK cell metabolic activity and sustained killing capacity [235]. The natural alkaloid piperlongumine significantly enhances tumor cell sensitivity to NK cell‐mediated killing by inducing ROS accumulation within tumor cells, disrupting protein homeostasis, and enhancing NK cell binding to tumor cells. This provides new evidence for utilizing natural products to synergistically enhance NK cell‐based immunotherapy [236].

### 7.4 T Lymphocytes (CD4^+^ and CD8^+^ T Cells)

Trichosanthin can alleviate immunosuppression by modulating the TGF‐β signaling pathway and inhibiting the function of regulatory T cells [237]. CUR promotes the proliferation and effector functions of CD8^+^ T cells, enhancing cytotoxic T lymphocyte responses [238]. Artesunate targets Ido1 to elevate tryptophan levels, thereby inhibiting NFATc1‐mediated PD1 transcription and activating CD8 T cells [239]. The structurally unique arabinogalactan‐polysaccharide fraction NLBPE1, isolated and purified from Lycium barbarum, induces proinflammatory cytokine release by activating the TLR4–IKK–NF‐κB signaling axis in DCs. This efficiently initiates antigen‐specific CD8^+^ T cell responses, demonstrating potent tumor suppression and preventive effects across multiple melanoma models [240]. Collectively, these actions enhance T cell‐mediated specific antitumor immune responses.

### 7.5 Myeloid‐Derived Suppressor Cells

Myeloid‐derived suppressor cells (MDSCs) promote immune evasion by inhibiting T cell activity in the TME. Their function is closely associated with lipid uptake and accumulation, a mechanism that enhances their immunosuppressive phenotype [241]. Studies indicate that soy isoflavones reduce lipid production in the TME, potentially weakening the function of MDSCs and reactivating T cell responses [226]. The traditional Chinese medicine formulation HBK, composed of 17 herbs and foods, modulates tumor immunity by promoting MDSC reduction through inhibition of the TLR3/NF‐κB signaling pathway [242]. Among bioactive plant‐derived compounds, sanguinarine has broad therapeutic applications in lung cancer. It inhibits M2 macrophage polarization through the Wnt/β‐catenin pathway, thereby blocking angiogenesis in lung cancer. Additionally, it induces the differentiation of MDSCs into macrophages and DCs via the NF‐κB pathway [243].

## 8 Delivery Systems and Formulation Innovation

Innovation in natural product delivery systems is key to enhancing their clinical efficacy. Intelligent delivery systems responsive to tumor metabolism and microenvironments, novel nanocarriers for oral and mucosal administration, and combined delivery systems integrating natural products with chemotherapy or immunotherapy collectively demonstrate the pivotal role of delivery technology innovation in advancing the clinical translation of natural products [244, 245, 246].

### 8.1 Natural Product‐Targeted Delivery Systems: Tumor Metabolism and Microenvironmental Adaptation

The therapeutic efficacy of natural products is often limited by their poor bioavailability and nonspecific distribution. To address these challenges, researchers are developing advanced delivery systems targeting tumor‐specific metabolic pathways and microenvironmental characteristics. These systems leverage the unique properties of tumor cells and their surrounding environment to achieve targeted delivery [247, 248].

Regarding tumor metabolic adaptation, natural product delivery systems can be engineered to target key metabolic pathways. For example, a nanocarrier loaded with mangiferin and CUR can effectively regulate tumor metabolism and induce apoptosis by synergistically inhibiting the PI3K/Akt/mTOR pathway in ovarian cancer. This nanomedicine also significantly enhances drug bioavailability and therapeutic efficacy [249]. Collagen‐modified nanoliposomes loaded with ginkgo biloba leaf and green tea extracts can induce apoptosis and arrest tumor cell cycles at the G0/G1 phase, demonstrating significant efficacy against hepatocellular carcinoma and colorectal cancer [250]. MPEG‐b‐PLA diblock copolymer micelles encapsulate the natural anticancer compound podophyllotoxin and its source, juniper extract. This carrier not only significantly enhances the drug's water solubility and stability but also selectively induces apoptosis in skin squamous cell carcinoma cells by inducing cell cycle arrest, activating caspase‐3, and generating ROS [251].

In terms of microenvironment adaptation, intelligent delivery systems can respond to specific physicochemical conditions within the TME to achieve precise drug release. By engineering nanoparticles to modulate their surface chemistry and functional groups for binding to receptors overexpressed on senescent cells, resveratrol‐loaded nanoparticles enhance targeting and uptake efficiency toward immune‐senescent cells within the TME [252]. The highly CAF‐specific nanomedicine system (Dex‐GP‐DOCA, DPD) developed by Huo's team responds to fibroblast activation protein‐alpha overexpressed on CAF surfaces, enabling precise release of the flavonoid compound quercetin. This targeted delivery achieves multiple effects including ECM degradation, vascular normalization, reversal of hypoxia resistance, and blockade of Wnt16 paracrine signaling [253]. Collectively, these cases demonstrate that microenvironment‐responsive smart delivery mechanisms can effectively intervene in tumor metabolism and matrix processes, enabling multifaceted synergistic regulation of the TME.

### 8.2 Nanocarriers for Oral and Mucosal Delivery of Natural Products

Natural products primarily originate from food and medicinal substances. Their oral administration method aligns closely with traditional medication habits, offering patients convenience and high compliance. Moreover, after absorption through the gastrointestinal mucosa, oral drugs can activate mucosal immune responses and leverage the mucosal system's efficient transport mechanisms to enhance systemic absorption. This approach avoids the trauma of injections while capitalizing on the unique advantages of the oral route [254]. However, the poor solubility and instability of natural products in the gastrointestinal tract present significant challenges. Nanocarrier systems offer a highly promising solution for enhancing oral bioavailability and enabling mucosal drug delivery.

Multiple nanotechnology approaches have been developed for oral delivery, including polymeric micelles (NPs) loaded with naringenin (NAR) designed for oral cancer treatment. Both free oral NAR and NAR‐NPs reversed lipid peroxidation and antioxidant status in DMBA‐treated animal buccal mucosal tissues. Compared with free NAR, NAR‐NPs exhibited higher antilipoperoxidation, antiproliferative, and antioxidant activities. NAR nanoparticles demonstrated significant chemopreventive potential by limiting or reducing abnormal cell growth in buccal mucosa through disruption of DMBA‐induced metabolic activation [245]. Inulin (IN) is a polymer with colon‐specific targeting capability, while hyaluronic acid (HA) targets CD44 on the surface of colon cancer cells. Existing research has developed an oral‐colon targeted delivery system (5‐Flu/MET@MSNs/Ce6@HIL) with HA and IN as key components, effectively inhibiting tumor growth [255].

Mucosal delivery systems offer an alternative pathway for local and systemic effects, enabling rapid absorption of natural products through the oral mucosa while bypassing hepatic metabolism [256]. Chemical constituents in blackberries have demonstrated preventive effects against multiple malignancies. A mucosal adhesion gel composed of Noveon AA1 and Carbopol 971 polymers has been developed and studied for site‐specific intraoral delivery of blackberry anthocyanins. Gel pH and storage temperature significantly impact blackberry anthocyanins chemical stability and mucosal permeability. The mucosal adhesion gel exhibits optimal stability for blackberry anthocyanins. Following incubation with human oral mucosal explants, anthocyanins rapidly diffuse into the human oral mucosa [257].

### 8.3 Co‐delivery Systems for Combination Therapy: Natural Products + Chemotherapy or Immunotherapy

Combination therapy strategies integrating natural products with conventional treatments represent a novel research focus. This approach enhances therapeutic efficacy while reducing side effects, producing synergistic effects [246].

In chemotherapy combination approaches, multiple drugs have been studied and developed. Quercetin competitively blocks various multidrug resistance efflux transporters, such as P‐gp, MRP‐1, and BCPR. Quercetin nanocrystals stabilized by glycyrrhizic acid (QT‐NCs/GL) successfully achieved liver‐targeted delivery, demonstrating significantly higher liver distribution compared with conventional nanocrystals (QT‐NCs/P188) in vivo. This provides an effective targeted delivery strategy for treating liver cancer using the combination of quercetin and glycyrrhizic acid [258]. A nanoscale system based on HA self‐assembly for targeted delivery of CUR (CUR@CS‐NP@HA) achieves active targeting of triple‐negative breast cancer cells expressing high levels of CD44. The nanoparticles exhibit pH‐responsive drug release properties, accelerating drug delivery within the acidic TME. Concurrently, CUR@CS‐NP@HA significantly enhances drug accumulation in tumor tissues, prolongs circulation time, improves bioavailability, and effectively inhibits tumor growth and metastasis [259]. The cabazitaxel liposomes modified with ginsenoside Rk1 (Rk1/CTX‐Lip) also serve as a typical example. This system utilizes Rk1 to replace cholesterol in constructing liposomes, which not only enhances drug targeting and stability but also achieves synergistic antitumor effects through the immunomodulatory action of Rk1 combined with cabazitaxel, while significantly reducing chemotherapy‐induced immunosuppression and infection risks [260].

In immunotherapy combinations, natural products modulate the TME to enhance immune responses. Nanoparticles codelivering anti‐PD‐1 antibodies and EGCG demonstrate improved T‐cell activation and tumor infiltration [261]. Micelles containing natural product immunomodulators (e.g., ginsenosides) with ICIs promote DC maturation and antigen presentation [262]. These approaches leverage the immunomodulatory properties of natural products to overcome resistance to conventional immunotherapies.

## 9 Clinical Translation and Precision Strategies

Natural products hold immense potential for clinical translation and precision applications in oncology [263]. We propose four approaches to achieve precise clinical translation of natural products: patient stratification based on molecular phenotypes and the immune microenvironment; promoting multimodal combinations of natural products with conventional therapies; personalized nutritional interventions guided by biomarkers; and concurrently advancing evidence accumulation and standardization in clinical translation. These strategies provide an implementation pathway for the scientific and rational application of natural products in precision oncology.

### 9.1 Patient Stratification for Natural Product‐Based Therapy

Although the biological activity of natural products has been well established, their therapeutic efficacy varies significantly across patient populations due to substantial differences in tumor biology, host metabolism, and genetic makeup. This variability underscores the necessity of stratification based on tumor‐specific signaling pathway abnormalities to identify individuals most likely to benefit from specific natural product interventions [264].

Natural product monomers can be selected based on specific signaling pathway abnormalities in a patient's tumor. For example, quercetin and CUR demonstrate significant therapeutic potential in tumors with abnormally activated Notch signaling pathways. Quercetin blocks downstream target gene expression by inhibiting proteolytic activation of Notch receptor proteins, while CUR effectively reverses chemotherapy resistance mediated by the Notch pathway [265, 266]. In tumors with overactivated PI3K/AKT/mTOR pathways, artemisinin and EGCG demonstrate multitarget regulatory advantages. Artemisinin simultaneously regulates the PI3K/AKT signaling axis and mTOR pathway, while EGCG synergistically induces programmed cell death through multiple nodes including p53 and Akt [267, 268]. A similar layered strategy applies to tumors with NF‐κB pathway overactivation, where ginsenoside Rg3 blocks signaling by inhibiting IκBα phosphorylation and p65 nuclear translocation [269].

The composition and functional state of the TIME can determine responses to natural product monomers. Certain natural compounds can reprogram the immune microenvironment, such as shifting TAMs from a protumor M2 phenotype to an antitumor M1 phenotype [219]. Baicalin has been demonstrated to promote TAM polarization toward M1, thereby alleviating the immunosuppressive environment [270]. For patients with immunologically “cold” tumors (characterized by insufficient T‐cell infiltration), natural product monomers like camptothecin and psoralen can induce immunogenic cell death (ICD), potentially transforming tumors into “hot” tumors and enhancing their sensitivity to immunotherapy. This enables stratification based on immune signatures such as the M1/M2 macrophage ratio or PD‐L1 expression [271].

Natural product monomers help overcome resistance to conventional therapies, providing a theoretical basis for stratification based on patient‐specific resistance mechanisms. For tumors exhibiting multidrug resistance due to overexpression of ABC transporters such as ABCG2, berberine inhibits these transporters and increases the accumulation of chemotherapeutic drugs within cancer cells [272]. In resistance scenarios linked to excessive inflammatory signaling, oxymatrine can reverse EMT‐associated resistance by inhibiting the NF‐κB pathway [273]. Furthermore, the natural compound acevaltrate can induce ferroptosis—a novel form of cell death—by dual‐targeting iron metabolism proteins (PCBP1/2) and the ferroptosis inhibitor GPX4, effectively eliminating drug‐resistant colorectal cancer cells [274].

In summary, these stratified strategies—ranging from molecular mechanisms to the immune microenvironment and specific resistance markers—enable the establishment of precision medicine models based on natural products. This approach allows the selection of natural products not only based on their general biochemical activity but also precisely matched to the individual tumor's biological characteristics, thereby unlocking immense potential for precision clinical oncology.

### 9.2 Integrating Natural Products Into Multimodal Clinical Regimens

The scientific integration of natural products into multimodal clinical treatment regimens represents a key strategy for enhancing cancer therapy outcomes. In current clinical practice, treatment strategies combining natural products with conventional therapies are widely adopted [246].

Regarding synergistic use with first‐line chemotherapeutic agents, multiple studies confirm that natural products can enhance the efficacy of chemotherapy drugs while reducing their toxic side effects. When CUR is combined with cisplatin, it not only increases tumor cell sensitivity to cisplatin by inhibiting the NF‐κB signaling pathway but also significantly mitigates cisplatin‐induced nephrotoxicity [275]. When combined with paclitaxel, baicalin reverses tumor cell multidrug resistance by regulating P‐gp function, thereby increasing paclitaxel accumulation in tumor tissues [276]. However, this combination strategy faces challenges such as unpredictable pharmacokinetic interactions and unclear optimal dosing sequences.

Combination therapy with ICIs shows promising prospects. Research indicates that resveratrol enhances PD‐1 inhibitor‐mediated antitumor immune responses by modulating gut microbiota composition, promoting DC maturation, and activating T cells [277]. Ginsenoside Rg3 improves the TIME by downregulating PD‐L1 expression in tumor cells, thereby increasing immunotherapy response rates [278]. These mechanisms provide a robust theoretical foundation for synergistic applications of natural products with immunotherapy.

Synergistic effects with metabolic inhibitors and antiangiogenic agents have also been extensively studied. Combining quercetin with bevacizumab not only enhances antiangiogenic effects but also delays resistance development by inhibiting the HIF‐1α/VEGF signaling pathway [279]. The combination of metformin and apigenin synergistically modulates the HGF/MET signaling pathway, inhibits MET‐mediated invasion, and influences downstream pathways such as PI3K/AKT, offering an innovative approach to enhance the efficacy and outcomes of cancer treatment [280].

Regarding application timing, natural products demonstrate unique value across different treatment phases. Preoperative administration of Ganoderma lucidum polysaccharides improves immune function, creating more favorable conditions for surgery [281]. Postoperative use of astragaloside IV promotes wound healing and reduces surgical stress responses [282]. During intervals between radiotherapy and chemotherapy, silymarin accelerates normal tissue repair and reduces cumulative toxicity [283]. These sequential application strategies provide comprehensive supportive care throughout the treatment journey for cancer patients.

However, integrating natural products into multimodal treatment regimens still faces challenges such as determining standardized dosages, assessing drug interactions, and optimizing treatment sequencing. Further clinical research is needed to establish appropriate combination therapies and fully leverage the potential of natural products in comprehensive cancer treatment.

### 9.3 Biomarker‐Driven Precision Nutrition and Supplementation

Natural products are playing an increasingly vital role in precision cancer therapy. Their interactions with biomarkers not only elucidate drug mechanisms of action but also drive paradigm shifts in biomarker discovery. Traditionally, biomarker identification relied primarily on comparative analysis between tumor and normal tissues [284]. However, natural small molecules, acting as “chemical probes,” offer a functionally guided approach to biomarker discovery.

By modulating pathways associated with tumor hallmarks, natural products directly alter the expression levels of specific molecular markers. This approach not only validates drug targets but also uncovers potential biomarkers. For instance, EGCG effectively inhibits tumor invasion and metastasis by suppressing MMP‐9 and upregulating E‐cadherin [285]. Resveratrol and quercetin reverse the Warburg effect by downregulating glycolytic key molecules such as lactate dehydrogenase (LDHA) and GLUT1. These changes not only demonstrate the metabolic regulatory function of natural products but also establish LDHA and GLUT1 as functional biomarkers for assessing tumor metabolic status [286]. Similarly, baicalin suppresses the STAT3 signaling pathway to reduce PD‐L1 expression, thereby modulating the TIME while suggesting that p‐STAT3 and PD‐L1 can serve as monitoring indicators for immunomodulatory therapy efficacy [287].

More importantly, natural products play a unique “reverse‐engineering” role in biomarker discovery. Unlike traditional “pathological differential” approaches, this mechanism‐driven method starts with natural small‐molecule interventions and identifies key functional molecules by analyzing their induced systemic responses. In colorectal cancer research, the natural compound acevaltrate was revealed to potently induce ferroptosis in cancer cells by dual‐targeting PCBP1/2 and GPX4 proteins. Based on this mechanism, the expression levels of PCBP1/2 and GPX4 can serve as potential biomarkers for predicting acevaltrate treatment sensitivity [274]. Furthermore, studies indicate that Tripterygium glycosides and camptothecin can significantly regulate the expression profiles of characteristic miRNAs in osteosarcoma cells. These miRNA populations, specifically modulated by natural products, are emerging as novel molecular markers for evaluating the anticancer effects of dual‐purpose medicinal and edible compounds. This approach reveals functional sensitive sites undetectable by conventional pathological differential analysis, thereby identifying therapeutic response biomarkers with higher clinical guidance value [288].

In summary, the interaction between natural products and biomarkers is propelling the advancement of precision cancer therapy. Clinically, by monitoring the dynamic changes in biomarkers following natural product interventions, physicians can not only objectively evaluate the comprehensive regulatory effects of drugs on tumor metabolism, apoptosis, and immunity but also identify patient populations sensitive to specific natural products based on “reverse‐guided” biomarker discovery, thereby achieving true personalized treatment. This mutually reinforcing and inspiring model between natural products and biomarkers provides more precise scientific rationale for the clinical translation of natural products [289].

### 9.4 Clinical Trials, Real‐World Evidence, and Translational Challenges

These findings highlight the emerging role of natural products—not only as therapeutic agents but also as multidimensional regulatory tools in biomarker‐guided precision nutrition and oncology. By modulating tumor‐specific characteristics, natural product‐based interventions enable personalized customization, enhancing therapeutic efficacy while minimizing adverse reactions and drug resistance [290].

At the epigenetic regulation level, natural products exhibit remarkable potential. Genistein from soy isoflavones reverses the silencing of tumor suppressor genes by inhibiting DNA methyltransferase activity; resveratrol modulates the expression of multiple tumor‐associated genes by activating the histone deacetylase SIRT1; EGCG in tea polyphenols influences the malignant phenotype of tumor cells by regulating the microRNA expression profile [76, 288, 291].

From multiple perspectives, natural products demonstrate significant advantages at cellular, microenvironmental, and clinical levels. At the cellular level, terpenoids like Tripterygium lactones effectively inhibit tumor proliferation by inducing DNA damage and cell cycle arrest; alkaloids such as vincristine block tumor cell mitosis by interfering with tubulin polymerization; while polysaccharides like Ganoderma lucidum polysaccharides enhance the body's antitumor immune response by activating immune‐related signaling pathways [36, 281].

At the TME level, saponins like ginsenoside Rg3 reduce tumor invasion and metastasis by inhibiting MMP‐2/9 activity; polyphenols improve the immunosuppressive microenvironment by modulating TAM polarization; wedelolactone is a naturally occurring coumarin that enhances IFN‐γ signaling by inhibiting the phosphatase activity of T‐cell protein tyrosine phosphatase, thereby promoting apoptosis in tumor cells via a STAT1‐dependent mechanism [227, 292, 293].

Clinically, natural products’ multitargeted properties, low toxicity, and ability to modulate multiple biomarkers (e.g., PD‐L1, CTLA‐4, VEGF) make them ideal candidates for personalized cancer therapy. For instance, alkaloids like homoharringtonine demonstrate unique advantages in leukemia treatment by inducing cell differentiation; flavonoids like baicalin demonstrate significant efficacy in treating precancerous lesions by suppressing inflammatory factor expression [108, 294].

Natural products have emerged as a key focus in innovative drug development for cancer treatment. Among those successfully applied clinically, paclitaxel is widely used for breast and ovarian cancer; camptothecin and its derivatives, such as irinotecan, target colorectal cancer and leukemia; while vincristine and vinblastine serve as core agents in treatment regimens for leukemia, lymphoma, and various solid tumors [21, 36, 37]. In recent years, chlorogenic acid for injection has demonstrated promising results in clinical studies for treating recurrent Grade IV glioblastoma. Early research data indicate that it significantly prolongs median survival in patients [295]. Among promising drug candidates, the small molecule 3,4‐diisobutyryl derivative of auxarthrol A—isolated from endophytic fungi of Euphorbiaceae plants and chemically modified—directly targets the LIC1 protein. It inhibits NSCLC tumor growth by inducing autophagic cell death and enhancing tumor sensitivity to anti‐PD‐1 immunotherapy [296]. Furthermore, multiple studies indicate that various natural plant bioactive compounds can reverse tumor multidrug resistance by downregulating P‐gp expression on tumor cell membranes, offering a potential strategy to overcome clinical chemotherapy resistance [9, 258, 276].

Meanwhile, real‐world studies further support the value of natural products in cancer prevention and long‐term maintenance therapy. For instance, a prospective cohort study involving 820 Greek women demonstrated a significant inverse association between flavonoid intake and breast cancer risk: a 13% reduction in risk for every 0.5 mg increase in daily intake. Specific dietary flavonoids may exert preventive effects against breast cancer, providing crucial epidemiological evidence for the cancer‐preventive potential of natural products [297]. Another study involving patients with postoperative tumor‐free metastatic colorectal cancer demonstrated that adding Qu‐yu‐jie‐du decoction (QYJD) to standard care for 2 years significantly improved disease‐free survival and showed a trend toward overall survival benefit. The study also found QYJD treatment reduced systemic inflammatory markers and lactate dehydrogenase levels with good safety [298]. This provides clinical‐grade evidence for natural products as low‐toxicity, effective maintenance strategies in preventing tumor recurrence and metastasis. Natural products demonstrate significant adjunctive value in postcancer surgery and long‐term care. Danggui Buxue Decoction significantly reduces the incidence and severity of chemotherapy‐induced thrombocytopenia in patients with malignant solid tumors, minimizing treatment interruptions [299]. The Japanese Kampo medicine Yokukansan prevents delirium following major cancer surgery in elderly patients by regulating plasma phospholipid metabolism, with its mechanism of alleviating cerebral oxidative stress validated in animal models [300].

Despite these significant achievements, the clinical translation of natural products still faces numerous critical challenges that extend far beyond target identification and drug properties to include key aspects such as quality control and standardization. Take the study on HBK compound herbal preparations as an example: This research employed UHPLC–ESI–Q‐TOF–MS/MS technology to establish a chemical fingerprint profile of the compound formulation, precisely identifying seven phytochemical markers including rutin and astragaloside IV, along with three nutritional components such as β‐glucan. Simultaneously adhering to Chinese Pharmacopoeia standards, it rigorously monitored safety indicators including heavy metals, pesticide residues, and microbial limits, fully demonstrating the practical application of natural product standardization. These systematic quality control practices provide critical technical support for the clinical translation of natural products [242].

**FIGURE 6:**
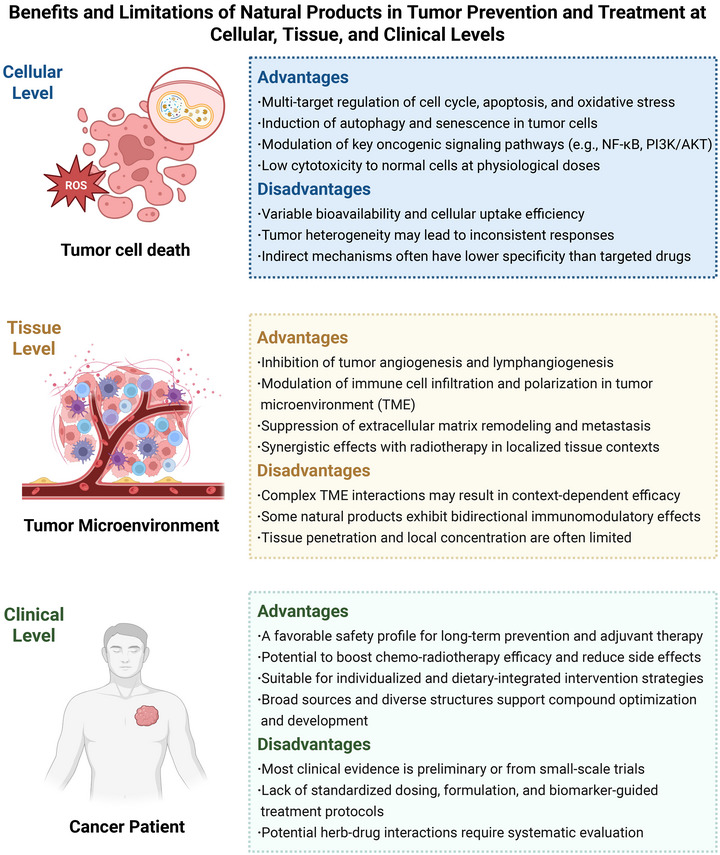
Benefits and limitations of natural products in tumor prevention and treatment at cellular, tissue, and clinical levels.

With continuous advancements in chemical biology, nanotechnology, and synthetic biology, multiple challenges in the clinical translation of natural products are gradually being overcome [301, 302]. Future research should focus on establishing more comprehensive quality control standards, strengthening the evidence base of evidence‐based medicine, and promoting the deep integration of natural products with precision medicine. This will ultimately provide cancer patients with safer and more effective personalized treatment options (Figure 6).

## 10 Conclusion

Natural products demonstrate multidimensional value in cancer prevention and treatment, with their core advantage lying in influencing the entire process of tumorigenesis and progression through multitarget regulatory mechanisms. These compounds not only directly intervene in cancer cell metabolic reprogramming, induce apoptosis, and inhibit metastasis, but also reshape the TME and modulate immune cell function to achieve synergistic tumor suppression. From prevention to treatment, from adjuvant radiotherapy and chemotherapy to improving quality of life, natural products offer unique complementary strategies for tumor therapy.

However, the journey from laboratory to clinical application presents significant challenges. Issues such as low bioavailability, complex quality control, and substantial interindividual variability in efficacy remain major obstacles. Fully realizing their potential necessitates multifaceted strategies. These include leveraging nanotechnology and novel delivery systems to enhance bioavailability, employing biomarker‐driven approaches for patient stratification, and integrating rigorous clinical trials with real‐world evidence. The future of natural products in oncology lies in their integration into multimodal and precision treatment regimens, transforming them into evidence‐based, effective components of precision cancer medicine. This ultimately offers safer, more personalized therapeutic options for patients worldwide.

In summary, natural products not only exert multifaceted preventive and therapeutic effects against cancer but also demonstrate highly promising adjunctive benefits when combined with metabolic targeting and immune response strategies. Moving forward, interdisciplinary collaboration among food science, oncology, and systems biology will be pivotal in transforming these natural compounds into effective tools for precision cancer therapy.

## Author Contributions

Ruimiao Qian, Hui Fu, and Jun Ge framed the synthesis. Ni Fan and Zheng Sun collected literature and sifted through articles. Ruimiao Qian, Chengcheng Zhao, and Yunfei Li wrote the manuscript and drew diagrams. Hui Fu, Yujiao Sun, and Yingpeng Li proposed the project and revised the manuscript. All authors have contributed to the article and approved the submitted version.

## Ethics Statement

The authors have nothing to report.

## Conflicts of Interest

The authors declare no conflicts of interest.

## Data Availability

The authors have nothing to report.

## References

[1] Y. Zhu , Z. J. Ouyang , H. J. Du , et al., “New Opportunities and Challenges of Natural Products Research: When Target Identification Meets Single‐cell Multiomics,” Acta Pharmaceutica Sinica B 12, no. 11 (2022): 4011–4039. 36386472 10.1016/j.apsb.2022.08.022 PMC9643300

[2] S. H. Lee , J. K. Ryu , K. Y. Lee , et al., “Enhanced Anti‐tumor Effect of Combination Therapy With Gemcitabine and Apigenin in Pancreatic Cancer,” Cancer Letters 259, no. 1 (2008): 39–49. 17967505 10.1016/j.canlet.2007.09.015

[3] E. A. Mohamad , M. R. El‐Garhy , and M. M. Rageh , “Improving Cisplatin Chemotherapy in Vivo by Niosomal Propolis and Chrysin as Nanoadjuvants,” Medical Oncology 42, no. 12 (2025): 539. 41193918 10.1007/s12032-025-03105-5

[4] H. Shulan , N. Peng , L. Likun , et al., “Effectiveness of Yiqi Chupi Powder for Alleviating Cancer‐related Fatigue in Patients Following Colorectal Cancer Surgery: A Randomized Controlled Trial,” Journal of Traditional Chinese Medicine 45, no. 5 (2025): 1119–1126. 41015810 10.19852/j.cnki.jtcm.2025.05.017 PMC12454259

[5] F. Yang , Z. Li , H. Zhang , et al., “Natural Product Erianin: Mitigating FOLFOX Toxicity and Enhancing Against Colorectal Cancer,” Front Chem 13 (2025): 1650197. 40919243 10.3389/fchem.2025.1650197 PMC12411860

[6] J. Cai , Z. R. Xuan , Y. P. Wei , H. B. Yang , and H. Wang , “[Effects of perioperative administration of Rhubarb on acute inflammatory response in patients With gastric cancer],” Zhong Xi Yi Jie He Xue Bao = Journal of Chinese Integrative Medicine 3, no. 3 (2005): 195–198. 15885167 10.3736/jcim20050309

[7] Y. Zheng , N. Feng , C. L. Li , and Z. Q. Li , “Natural Products Target Programmed Cell Death Signaling Mechanisms to Treat Colorectal Cancer,” Frontiers In Pharmacology 16 (2025): 26. 10.3389/fphar.2025.1565332 PMC12058791 40342991

[8] C. Yu , C. Jia , G. Chen , et al., “Yiai Fuzheng Decoction Inhibits Triple‐negative Breast Cancer by Remodeling the Immune Microenvironment,” Frontiers In Immunology 16 (2025): 1615631. 41098716 10.3389/fimmu.2025.1615631 PMC12518410

[9] M. Demeule , M. Brossard , S. Turcotte , et al., “Diallyl Disulfide, a Chemopreventive Agent in Garlic, Induces Multidrug Resistance‐associated Protein 2 Expression,” Biochemical and Biophysical Research Communications 324, no. 2 (2004): 937–945. 15474518 10.1016/j.bbrc.2004.09.141

[10] A. B. P. van Kuilenburg , R. Meinsma , and A. H. van Gennip , “Pyrimidine Degradation Defects and Severe 5‐fluorouracil Toxicity,” Nucleosides Nucleotides & Nucleic Acids 23, no. 8‐9 (2004): 1371–1375. 10.1081/NCN-200027624 15571261

[11] M. M. Monteiro , B. B. Silveira , L. Di Carvalho Melo , et al., “Chitosan‐Based Systems for Curcumin Delivery: Evidence for Therapeutic Applications,” Journal of Biochemical and Molecular Toxicology 39, no. 10 (2025): e70515. 40995635 10.1002/jbt.70515

[12] K. Subban and F. Kempken , “Insights Into Taxol® Biosynthesis by Endophytic Fungi,” Applied Microbiology And Biotechnology 107, no. 20 (2023): 6151–6162. 37606790 10.1007/s00253-023-12713-y PMC10560151

[13] P. Y. Yao , S. Liang , Z. Y. Liu , and C. P. Xu , “A Review of Natural Products Targeting Tumor Immune Microenvironments for the Treatment of Lung Cancer,” Frontiers In Immunology 15 (2024): 26. 10.3389/fimmu.2024.1343316 PMC10867126 38361933

[14] C. Gerhäuser , “Cancer Cell Metabolism, Epigenetics and the Potential Influence of Dietary Components—A Perspective,” Biomedical Research‐India 23 (2012): 69–89.

[15] J. C. Wu , H. Ji , T. T. Li , et al., “Targeting the Prostate Tumor Microenvironment by Plant‐derived Natural Products,” Cellular Signalling 115 (2024): 15. 10.1016/j.cellsig.2023.111011 38104704

[16] Y. Hu , J. Z. Song , A. J. Feng , et al., “Recent Advances in Nanotechnology‐Based Targeted Delivery Systems of Active Constituents in Natural Medicines for Cancer Treatment,” Molecules (Basel, Switzerland) 28, no. 23 (2023): 33. 10.3390/molecules28237767 PMC10708032 38067497

[17] Z. Li , T. T. Zhao , J. X. Li , et al., “Nanomedicine Based on Natural Products: Improving Clinical Application Potential,” Journal Of Nanomaterials 2022 (2022): 11.

[18] S. Dutta , S. Mahalanobish , S. Saha , S. Ghosh , and P. C. Sil , “Natural Products: An Upcoming Therapeutic Approach to Cancer,” Food And Chemical Toxicology 128 (2019): 240–255. 30991130 10.1016/j.fct.2019.04.012

[19] A. W. Shi , L. Liu , S. Li , and B. Qi , “Natural Products Targeting the MAPK‐signaling Pathway in Cancer: Overview,” Journal Of Cancer Research And Clinical Oncology 150, no. 1 (2024): 20. 38193944 10.1007/s00432-023-05572-7 PMC10776710

[20] D. Zhou , Z. S. Bai , and T. T. Guo , “Dietary Flavonoids and human Top‐ranked Diseases: The Perspective of in Vivo Bioactivity and Bioavailability,” Trends In Food Science & Technology 120 (2022): 374–386.

[21] A. A. Khalil , A. Rauf , F. A. Alhumaydhi , et al., “Recent Developments and Anticancer Therapeutics of Paclitaxel: An Update,” Current Pharmaceutical Design 28, no. 41 (2022): 3363–3373. 36330627 10.2174/1381612829666221102155212

[22] W. X. Fan , L. H. Fan , Z. Y. Wang , et al., “Rare Ginsenosides: A Unique Perspective of Ginseng Research,” Journal Of Advanced Research 66 (2024): 303–328. 38195040 10.1016/j.jare.2024.01.003 PMC11674801

[23] D. Shah , N. Challagundla , V. Dave , et al., “Berberine Mediates Tumor Cell Death by Skewing Tumor‐associated Immunosuppressive Macrophages to Inflammatory Macrophages,” Phytomedicine 99 (2022): 12. 10.1016/j.phymed.2021.153904 35231825

[24] G. W. Qin and R. S. Xu , “Recent Advances on Bioactive Natural Products From Chinese Medicinal Plants,” Medicinal Research Reviews 18, no. 6 (1998): 375–382. 9828038 10.1002/(sici)1098-1128(199811)18:6<375::aid-med2>3.0.co;2-8

[25] B. Cong , “Perspectives in Food & Amp; Medicine Homology,” Food & Medicine Homology 1, no. 1 (2024): 9420018.

[26] H. Qamar , S. Rehman , and D. K. Chauhan , “Current Status and Future Perspective for Research on Medicinal Plants With Anticancerous Activity and Minimum Cytotoxic Value,” Current Drug Targets 20, no. 12 (2019): 1227–1243. 31486747 10.2174/1389450120666190429120314

[27] J. Li , S. Han , Y. Zhu , B. Dong , and A. Halorotetin , “A Novel Terpenoid Compound Isolated From Ascidian Halocynthia rotetzi Exhibits the Inhibition Activity on Tumor Cell Proliferation,” Marine Drugs 21, no. 1 (2023): 51. 36662224 10.3390/md21010051 PMC9860651

[28] B. Mubeen , A. Hasnain , S. A. H. Naqvi , et al., “Phytochemicals as Multi‐Target Therapeutic Agents for Oxidative Stress‐Driven Pathologies: Mechanisms, Synergies, and Clinical Prospects,” Phyton‐International Journal Of Experimental Botany 94, no. 7 (2025): 1941–1971.

[29] Y. Wang , X. Huang , J. Han , W. Zheng , and W. Ma , “Extract of Perilla Frutescens Inhibits Tumor Proliferation of HCC via PI3K/AKT Signal Pathway,” Afr J Tradit Complement Altern Med 10, no. 2 (2013): 251–257. 24146448 PMC3746572

[30] Y. Zhou , Y. Li , T. Zhou , et al., “Dietary Natural Products for Prevention and Treatment of Liver Cancer,” Nutrients 8, no. 3 (2016): 156. 26978396 10.3390/nu8030156 PMC4808884

[31] J. A. Ross and C. M. Kasum , “Dietary Flavonoids: Bioavailability, Metabolic Effects, and Safety,” Annual Review Of Nutrition 22 (2002): 19–34. 10.1146/annurev.nutr.22.111401.144957 12055336

[32] M. Li , M. Q. Qian , Q. Jiang , et al., “Evidence of Flavonoids on Disease Prevention,” Antioxidants 12, no. 2 (2023): 19. 10.3390/antiox12020527 PMC9952065 36830086

[33] T. Y. Wang , Q. Li , and K. S. Bi , “Bioactive Flavonoids in Medicinal Plants: Structure, Activity and Biological Fate,” Asian Journal Of Pharmaceutical Sciences 13, no. 1 (2018): 12–23. 32104374 10.1016/j.ajps.2017.08.004 PMC7032191

[34] X. Li , X. He , B. Lin , et al., “Quercetin Limits Tumor Immune Escape Through PDK1/CD47 Axis in Melanoma,” American Journal Of Chinese Medicine 52, no. 02 (2024): 541–563. 38490807 10.1142/S0192415X2450023X

[35] I. Ahmad , S. Fakhri , H. Khan , et al., “Targeting Cell Cycle by β‐carboline Alkaloids in Vitro: Novel Therapeutic Prospects for the Treatment of Cancer,” Chemico‐Biological Interactions 330 (2020): 9. 10.1016/j.cbi.2020.109229 32835667

[36] C. V. Rao , C. D. Kurkjian , and H. Y. Yamada , “Mitosis‐Targeting Natural Products for Cancer Prevention and Therapy,” Current Drug Targets 13, no. 14 (2012): 1820–1830. 23140292 10.2174/138945012804545533

[37] S. C. Leonard , H. Lee , D. F. Gaddy , et al., “Extended Topoisomerase 1 Inhibition Through Liposomal Irinotecan Results in Improved Efficacy Over topotecan and irinotecan in Models of Small‐cell Lung Cancer,” Anti‐Cancer Drugs 28, no. 10 (2017): 1086–1096. 28857767 10.1097/CAD.0000000000000545

[38] C. El‐Baba , A. Baassiri , G. Kiriako , et al., “Terpenoids' anti‐cancer Effects: Focus on Autophagy,” Apoptosis 26, no. 9‐10 (2021): 491–511. 34269920 10.1007/s10495-021-01684-y

[39] M. Asensi , A. Ortega , S. Mena , F. Feddi , and J. M. Estrela , “Natural Polyphenols in Cancer Therapy,” Critical Reviews In Clinical Laboratory Sciences 48, no. 5‐6 (2011): 197–216. 22141580 10.3109/10408363.2011.631268

[40] A. Brockmueller , S. Sameri , A. Liskova , et al., “Resveratrol's Anti‐Cancer Effects Through the Modulation of Tumor Glucose Metabolism,” Cancers 13, no. 2 (2021): 35. 10.3390/cancers13020188 PMC7825813 33430318

[41] K. W. Luo , W. Chen , W. Y. Lung , et al., “EGCG Inhibited Bladder Cancer SW780 Cell Proliferation and Migration both in Vitro and in Vivo via Down‐regulation of NF‐κB and MMP‐9,” Journal Of Nutritional Biochemistry 41 (2017): 56–64. 28040581 10.1016/j.jnutbio.2016.12.004

[42] V. M. Shah , S. Rizvi , A. Smith , et al., “Micelle‐Formulated Juglone Effectively Targets Pancreatic Cancer and Remodels the Tumor Microenvironment,” Pharmaceutics 15, no. 12 (2023): 26. 10.3390/pharmaceutics15122651 PMC10747591 38139993

[43] Z. T. Xu , X. U. Chen , Z. F. Zhong , L. D. Chen , and Y. T. Wang , “Ganoderma Lucidum Polysaccharides: Immunomodulation and Potential Anti‐Tumor Activities,” American Journal Of Chinese Medicine 39, no. 1 (2011): 15–27. 21213395 10.1142/S0192415X11008610

[44] G. Maskarinec , D. Ju , Y. Morimoto , A. A. Franke , and F. Z. Stanczyk , “Soy Food Intake and Biomarkers of Breast Cancer Risk: Possible Difference in Asian Women?,” Nutrition And Cancer 69, no. 1 (2017): 146–153. 27918846 10.1080/01635581.2017.1250924 PMC5248572

[45] M. E. Vaccari , V. Cavalloro , M. Bedeschi , et al., “Preparation of a Micronutrient‐Enriched Apricot Kernel Oil and Assessment of in Vitro Chemopreventive Properties,” International Journal Of Molecular Sciences 26, no. 18 (2025). 10.3390/ijms26189237 PMC12470769 41009803

[46] W. Rzeska , M. Chojnacki , A. Adamiak‐Godlewska , A. Semczuk , and M. K. Lemieszek , “First Look at Chemopreventive Properties of Chlorella Pyrenoidosa Water Extract in Human Endometrial Adenocarcinoma Cells‐Preliminary in Vitro Study,” International Journal Of Molecular Sciences 26, no. 18 (2025): 9142. 41009705 10.3390/ijms26189142 PMC12471056

[47] M. Ławiński , K. Zadka , N. Ksepka , et al., “Does Resveratrol Impact Oxidative Stress Markers in Patients With Head and Neck Cancer Receiving Home Enteral Nutrition?,” Nutrients 17, no. 3 (2025): 504. 39940362 10.3390/nu17030504 PMC11819975

[48] C. Cheon , H. Kim , S. Y. Kang , et al., “Safety Evaluation of SH003 and Docetaxel Combination in Patients with Breast and Lung Cancer: A Multi‐Center, Open‐Label, Dose Escalation Phase I Clinical Trial,” Integrative Cancer Therapies 24 (2025): 15347354251363892. 40782124 10.1177/15347354251363892 PMC12335644

[49] D. R. Ferry , A. Smith , J. Malkhandi , et al., “Phase I Clinical Trial of the Flavonoid Quercetin: Pharmacokinetics and Evidence for in Vivo Tyrosine Kinase Inhibition,” Clinical Cancer Research 2, no. 4 (1996): 659–668. 9816216

[50] X. Yin , W. Zhu , X. Tang , et al., “Phase I/II Clinical Trial of Efficacy and Safety of EGCG Oxygen Nebulization Inhalation in the Treatment of COVID‐19 Pneumonia Patients With Cancer,” BMC cancer 24, no. 1 (2024): 486. 38632501 10.1186/s12885-024-12228-3 PMC11022442

[51] T. Ge , H. Li , P. Xiang , et al., “Tanshinone IIA Induces Ferroptosis in Colorectal Cancer Cells Through the Suppression of SLC7A11 Expression via the PI3K/AKT/mTOR Pathway,” European Journal Of Medical Research 30, no. 1 (2025): 576. 40618168 10.1186/s40001-025-02842-7 PMC12228160

[52] K. Iwasaki , E. Barroga , Y. Takano , et al., “Effects of Rikkunshito on Postgastrectomy Weight Loss and Nutritional Status in Gastric Cancer Patients: A Retrospective Observational Study,” Medicine 104, no. 33 (2025): e43950. 40826685 10.1097/MD.0000000000043950 PMC12366934

[53] M. Wang , S. J. Yan , H. T. Zhang , et al., “Ginsenoside Rh2 Enhances the Antitumor Immunological Response of a Melanoma Mice Model,” Oncology Letters 13, no. 2 (2017): 681–685. 28356946 10.3892/ol.2016.5490 PMC5351349

[54] W. Xu , Y. Li , L. Liu , et al., “Icaritin‐curcumol Activates CD8(+) T Cells Through Regulation of Gut Microbiota and the DNMT1/IGFBP2 Axis to Suppress the Development of Prostate Cancer,” Journal Of Experimental & Clinical Cancer Research 43, no. 1 (2024): 149. 38778379 10.1186/s13046-024-03063-2 PMC11112810

[55] W. Gao , S. Xu , B. Kang , et al., “Sodium Hyaluronate‐modified Fe(III)‐shikonin Self‐assembly Nanoparticle for Effective Tumor Therapy and Reduced Tumor Metastasis,” Carbohydrate Polymers 367 (2025): 123939. 40817465 10.1016/j.carbpol.2025.123939

[56] Y. X. Chen , Q. Y. Gao , T. H. Zou , et al., “Berberine versus Placebo for the Prevention of Recurrence of Colorectal Adenoma: A Multicentre, Double‐blinded, Randomised Controlled Study,” Lancet Gastroenterol Hepatol 5, no. 3 (2020): 267–275. 31926918 10.1016/S2468-1253(19)30409-1

[57] W. U. Ruixin , F. Qingliang , G. Sisi , et al., “A Pilot Study of Precision Treatment for Patients With Lung Cancer Pain by Longteng Tongluo Recipe Using Serum Genomics,” Journal Of Traditional Chinese Medicine 44, no. 5 (2024): 1006–1016. 39380232 10.19852/j.cnki.jtcm.20240828.005 PMC11462530

[58] O. L. Elvir‐Lazo , P. F. White , H. C. Eng , et al., “Impact of Chronic Medications in the Perioperative Period: Mechanisms of Action and Adverse Drug Effects (Part I),” Postgraduate Medicine 133, no. 8 (2021): 939–952. 34551662 10.1080/00325481.2021.1982297

[59] Y. K. Liu , J. He , M. Li , K. B. Ren , and Z. H. Zhao , “Inflammation‐Driven Nanohitchhiker Enhances Postoperative Immunotherapy by Alleviating Prostaglandin E2‐Mediated Immunosuppression,” Acs Applied Materials & Interfaces 16, no. 6 (2024): 6879–6893. 38300288 10.1021/acsami.3c17357

[60] S. Takiguchi , Y. Hiura , T. Takahashi , et al., “Effect of Rikkunshito, a Japanese Herbal Medicine, on Gastrointestinal Symptoms and Ghrelin Levels in Gastric Cancer Patients After Gastrectomy,” Gastric Cancer 16, no. 2 (2013): 167–174. 22895614 10.1007/s10120-012-0164-3

[61] R. F. Ozols , “Challenges for Chemotherapy in Ovarian Cancer,” Annals Of Oncology 17 (2006): V181–V187. 16807453 10.1093/annonc/mdj978

[62] J. Jin , S. Ren , Q. Shi , et al., “Shengbai Oral Solution for Chemotherapy‐induced Neutropenia and Symptom Management in Non‐small Cell Lung Cancer: A Multicenter, Randomized, Open‐label Trial,” Phytomedicine 148 (2025): 157502. 41202374 10.1016/j.phymed.2025.157502

[63] J. R. Hu , Q. Ji , W. W. He , et al., “Efficacy of Modified Peanut Skin Decoction for Lung Cancer Myelosuppression,” Journal Of Visualized Experiments: JoVE no. 223 (2025). 10.3791/68880 41052050

[64] J. Zhang , L. Zhang , C. Wang , et al., “The Fritillaria Alkaloid Peiminine Acts as a Chemosensitizer to Potentiate Oxaliplatin Efficacy Against Gastric Cancer,” Pathology, Research and Practice 275 (2025): 156208. 40912051 10.1016/j.prp.2025.156208

[65] Q. Liu , Q. Zhou , X. Yang , et al., “Tanshinone IIA Is Synergistic With the PARP Inhibitor olaparib in Inducing BRCAs‐proficient and ‐deficient Triple‐negative Breast Cancer Cell Apoptosis,” Medical Oncology 42, no. 9 (2025): 419. 40782284 10.1007/s12032-025-02968-y

[66] J. Yao , J. Liu , Y. He , et al., “Systems Pharmacology Reveals the Mechanism of Astragaloside IV in Improving Immune Activity on Cyclophosphamide‐induced Immunosuppressed Mice,” Journal Of Ethnopharmacology 313 (2023): 116533. 37100262 10.1016/j.jep.2023.116533

[67] Y. Zhong , Q. Xiao , J. Huang , et al., “Ginsenoside Rg1 Alleviates Ulcerative Colitis in Obese Mice by Regulating the Gut Microbiota‐Lipid Metabolism‐Th1/Th2/Th17 Cells Axis,” Journal Of Agricultural And Food Chemistry 71, no. 50 (2023): 20073–20091. 38064669 10.1021/acs.jafc.3c04811

[68] M. Y. Huang , Y. C. Chen , W. Y. Lyu , et al., “Ginsenoside Rh2 Augmented Anti‐PD‐L1 Immunotherapy by Reinvigorating CD8(+) T Cells via Increasing Intratumoral CXCL10,” Pharmacological Research 198 (2023): 106988. 37984507 10.1016/j.phrs.2023.106988

[69] X. Lu , A. Wang , D. Chen , et al., “Gegen Qinlian Tablets Attenuate Immune‐related Adverse Events in NSCLC Patients: A Multi‐center Randomized Controlled Trial in China,” Phytomedicine 145 (2025): 156968. 40561860 10.1016/j.phymed.2025.156968

[70] J. Y. Shi , Y. J. Wang , Q. W. Bao , et al., “Polygonatum Cyrtonema Hua Polysaccharide Alleviates Ulcerative Colitis via Gut Microbiota‐independent Modulation of Inflammatory Immune Response,” Carbohydrate Polymers 356 (2025): 19. 10.1016/j.carbpol.2025.123387 40049966

[71] M. Dong , Z. Meng , K. Kuerban , et al., “Diosgenin Promotes Antitumor Immunity and PD‐1 Antibody Efficacy Against Melanoma by Regulating Intestinal Microbiota,” Cell Death & Disease 9, no. 10 (2018): 1039. 30305604 10.1038/s41419-018-1099-3 PMC6179990

[72] B. Li , T. Xiang , M. Bindawa Isah , C. Chen , and X. Zhang , “In Vitro Simulated Saliva, Gastric, and Intestinal Digestion Followed by Faecal Fermentation Reveals a Potential Modulatory Activity of Epimedium on human Gut Microbiota,” Journal Of Pharmaceutical And Biomedical Analysis 245 (2024): 116151. 38652940 10.1016/j.jpba.2024.116151

[73] H. Tazoe , Y. Otomo , I. Kaji , et al., “Roles of Short‐chain Fatty Acids Receptors, GPR41 and GPR43 on Colonic Functions,” Journal Of Physiology And Pharmacology 59 (2008): 251–262. Suppl 2. 18812643

[74] M. G. Bae , J. Hwang‐Bo , D. Y. Lee , Y. H. Lee , and I. S. Chung , “Effects of 6,8‐Diprenylgenistein on VEGF‐A‐Induced Lymphangiogenesis and Lymph Node Metastasis in an Oral Cancer Sentinel Lymph Node Animal Model,” International Journal Of Molecular Sciences 22, no. 2 (2021). 10.3390/ijms22020770 PMC7828717 33466636

[75] L. Avila‐Carrasco , P. Majano , J. A. Sánchez‐Toméro , et al., “Natural Plants Compounds as Modulators of Epithelial‐to‐Mesenchymal Transition,” Frontiers In Pharmacology 10 (2019): 715. 31417401 10.3389/fphar.2019.00715 PMC6682706

[76] W. Liu , M. Hu , F. Cao , and S. Jin , “Polyphenols and Exercise in Autophagy Regulation: Potential Benefits for Cancer Management and Healthspan,” Frontiers in Nutrition 12 (2025): 1618813. 40989799 10.3389/fnut.2025.1618813 PMC12450968

[77] C. Buhrmann , P. Shayan , A. Brockmueller , and M. Shakibaei , “Resveratrol Suppresses Cross‐Talk Between Colorectal Cancer Cells and Stromal Cells in Multicellular Tumor Microenvironment: A Bridge Between in Vitro and in Vivo Tumor Microenvironment Study,” Molecules (Basel, Switzerland) 25, no. 18 (2020): 4292. 32962102 10.3390/molecules25184292 PMC7570736

[78] Y. Hijikata , “Analgesic Treatment With Kampo Prescription,” Expert Review Of Neurotherapeutics 6, no. 5 (2006): 795–802. 16734526 10.1586/14737175.6.5.795

[79] F. Inferrera , N. Tranchida , R. Fusco , et al., “Neuronutritional Enhancement of Antioxidant Defense System Through Nrf2/HO1/NQO1 Axis in Fibromyalgia,” Neurochemistry International 190 (2025): 106057. 40997946 10.1016/j.neuint.2025.106057

[80] Z. Y. Su , Z. Z. Yang , Y. Q. Xu , Y. B. Chen , and Q. Yu , “Apoptosis, Autophagy, Necroptosis, and Cancer Metastasis,” Molecular Cancer 14 (2015): 14. 25743109 10.1186/s12943-015-0321-5 PMC4343053

[81] J. D. Martin , G. Seano , and R. K. Jain , “Normalizing Function of Tumor Vessels: Progress, Opportunities, and Challenges,” Annual Review Of Physiology 81 (2019): 505–534. 10.1146/annurev-physiol-020518-114700 PMC6571025 30742782

[82] F. Cao , H. L. Zhang , C. Guo , X. L. Xu , and Q. Yuan , “Targeting Oxidative Stress With Natural Products: A Novel Strategy for Esophageal Cancer Therapy,” World Journal Of Gastrointestinal Oncology 16, no. 2 (2024): 14. 10.4251/wjgo.v16.i2.287 PMC10900143 38425393

[83] A. R. Guerra , M. F. Duarte , and I. F. Duarte , “Targeting Tumor Metabolism With Plant‐Derived Natural Products: Emerging Trends in Cancer Therapy,” Journal Of Agricultural And Food Chemistry 66, no. 41 (2018): 10663–10685. 30227704 10.1021/acs.jafc.8b04104

[84] C. M. Pfeffer and A. T. K. Singh , “Apoptosis: A Target for Anticancer Therapy,” International Journal Of Molecular Sciences 19, no. 2 (2018): 10. 10.3390/ijms19020448 PMC5855670 29393886

[85] K. J. Campbell and S. W. G. Tait , “Targeting BCL‐2 Regulated Apoptosis in Cancer,” Open Biology 8, no. 5 (2018): 11. 10.1098/rsob.180002 PMC5990650 29769323

[86] B. Tummers and D. R. Green , “Caspase‐8: Regulating Life and Death,” Immunological Reviews 277, no. 1 (2017): 76–89. 28462525 10.1111/imr.12541 PMC5417704

[87] S. N. Qian , Z. Wei , W. T. Yang , et al., “The Role of BCL‐2 family Proteins in Regulating Apoptosis and Cancer Therapy,” Frontiers In Oncology 12 (2022): 16. 10.3389/fonc.2022.985363 PMC9597512 36313628

[88] B. K. Utpal , H. Bouenni , M. Zehravi , et al., “Exploring Natural Products as Apoptosis Modulators in Cancers: Insights Into Natural Product‐based Therapeutic Strategies,” Naunyn‐Schmiedebergs Archives Of Pharmacology 398, no. 7 (2025): 8189–8214. 40014131 10.1007/s00210-025-03876-8

[89] G. Moriasi , M. Ngugi , P. Mwitari , and G. Omwenga , “Promising Anti‐cervical Cancer Efficacy and Phytochemical Analysis of Vitex doniana Sweet (Verbenaceae) Leaf Extracts: An in Vitro Investigation With Molecular Mechanisms of Action,” BMC Complement Med Ther 25, no. 1 (2025): 379. 41102795 10.1186/s12906-025-04923-w PMC12532901

[90] M. A. Ghareeb , B. Bakchiche , Y. Aouiffat , et al., “LC/ESI‐MS/MS Phytochemical Profiling and Apoptotic Effect of Haloxylon Scoparium Leaf Extract on Hepatocellular Carcinoma,” Scientific Reports 15, no. 1 (2025): 37154. 41131348 10.1038/s41598-025-21041-2 PMC12549966

[91] L. Liu , Y. Liu , S. Zhang , et al., “Celastrol Promotes Apoptosis of Breast Cancer MDA‐MB‐231 Cells by Targeting HSDL2,” Acupuncture And Herbal Medicine 4, no. 1 (2024): 92–101.

[92] G. Sahoo , D. Samal , P. Khandayataray , and M. K. Murthy , “A Review on Caspases: Key Regulators of Biological Activities and Apoptosis,” Molecular Neurobiology 60, no. 10 (2023): 5805–5837. 37349620 10.1007/s12035-023-03433-5

[93] M. Asadi , S. Taghizadeh , E. Kaviani , et al., “Caspase‐3: Structure, Function, and Biotechnological Aspects,” Biotechnology And Applied Biochemistry 69, no. 4 (2022): 1633–1645. 34342377 10.1002/bab.2233

[94] D. Shekarabi , S. Safi , and P. Mortazavi , “Evaluation of Apoptosis and Caspase‐3 Activity in EL4 Cell Line Lymphoma Using Moringa Oleifera Plant Extract,” Archives of Razi Institute 80, no. 1 (2025): 37–50. 40951560 10.32592/ARI.2025.80.1.37 PMC12426439

[95] A. M. Mousa , N. M. El‐Sammad , A. H. Abdel‐Halim , et al., “Lagerstroemia Speciosa (L.) Pers Leaf Extract Attenuates Lung Tumorigenesis via Alleviating Oxidative Stress,” Biomolecules 9, no. 12 (2019). 10.3390/biom9120871 PMC6995620 31842482

[96] B. Pucci , M. Kasten , and A. Giordano , “Cell Cycle and Apoptosis,” Neoplasia 2, no. 4 (2000): 291–299. 11005563 10.1038/sj.neo.7900101 PMC1550296

[97] M. Zhong , Y. Li , H. Wang , et al., “Integrated Network Pharmacology and Experimental Validation Reveal EGFR/p53/Bcl‐2‐mediated Anti‐hepatocellular Carcinoma Effects of Zedoary Turmeric Oil,” Journal Of Ethnopharmacology 352 (2025): 120241. 40615101 10.1016/j.jep.2025.120241

[98] T. H. Wang , H. S. Wang , and Y. K. Soong , “Paclitaxel‐induced Cell Death ‐: Where the Cell Cycle and Apoptosis Come Together,” Cancer 88, no. 11 (2000): 2619–2628. 10861441 10.1002/1097-0142(20000601)88:11<2619::aid-cncr26>3.0.co;2-j

[99] S. J. Choi , J. H. Kim , H. J. Kim , et al., “Triggering Mitotic Catastrophe by Podophyllotoxin Induces Apoptosis in Oral Squamous Cell Carcinoma,” Archives Of Oral Biology 179 (2025): 10. 10.1016/j.archoralbio.2025.106396 40966849

[100] O. Prakash , R. Singh , N. Singh , et al., “Anticancer Potential of Naringenin, Biosynthesis, Molecular Target, and Structural Perspectives,” Mini ‐ Reviews In Medicinal Chemistry 22, no. 5 (2022): 758–769. 34517796 10.2174/1389557521666210913112733

[101] Y. Huang , Y. Zhou , Y. Fan , and D. Zhou , “Celastrol Inhibits the Growth of human Glioma Xenografts in Nude Mice Through Suppressing VEGFR Expression,” Cancer Letters 264, no. 1 (2008): 101–106. 18343027 10.1016/j.canlet.2008.01.043

[102] Z. Li , Q. Zhang , X. Zhang , et al., “Dihydroartemisinin Inhibits Melanoma Migration and Metastasis by Affecting Angiogenesis,” Phytotherapy Research 39, no. 4 (2025): 1679–1693. 37982352 10.1002/ptr.8065 PMC12013856

[103] M. Najafi , B. Farhood , and K. Mortezaee , “Extracellular Matrix (ECM) Stiffness and Degradation as Cancer Drivers,” Journal Of Cellular Biochemistry 120, no. 3 (2019): 2782–2790. 30321449 10.1002/jcb.27681

[104] R. A. Reddy , M. S. Varshini , and R. S. Kumar , “Matrix Metalloproteinase‐2 (MMP‐2): As an Essential Factor in Cancer Progression,” Recent Patents On Anti‐Cancer Drug Discovery 20, no. 1 (2025): 26–44. 37861020 10.2174/0115748928251754230922095544

[105] N. M. Abdel‐Hamid and S. A. Abass , “Matrix Metalloproteinase Contribution in Management of Cancer Proliferation, Metastasis and Drug Targeting,” Molecular Biology Reports 48, no. 9 (2021): 6525–6538. 34379286 10.1007/s11033-021-06635-z

[106] X. Peng , Q. Zhang , Y. Zeng , et al., “Evodiamine Inhibits the Migration and Invasion of Nasopharyngeal Carcinoma Cells in Vitro via Repressing MMP‐2 Expression,” Cancer Chemotheraphy And Pharmacology 76, no. 6 (2015): 1173–1184. 10.1007/s00280-015-2902-9 26546460

[107] T. Zhou , A. J. Zhang , G. Kuang , et al., “Baicalin Inhibits the Metastasis of Highly Aggressive Breast Cancer Cells by Reversing Epithelial‐to‐mesenchymal Transition by Targeting β‐catenin Signaling,” Oncology Reports 38, no. 6 (2017): 3599–3607. 29039569 10.3892/or.2017.6011

[108] X. F. Wang , Q. M. Zhou , J. Du , et al., “Baicalin Suppresses Migration, Invasion and Metastasis of Breast Cancer via p38MAPK Signaling Pathway,” Anti‐Cancer Agents In Medicinal Chemistry 13, no. 6 (2013): 923–931. 23387975 10.2174/18715206113139990143

[109] Ü. Yırtıcı , A. Ergene , Ş. Adem , et al., “Centaurea Mersinensis Phytochemical Composition and Multi‐dimensional Bioactivity Properties Supported by Molecular Modeling,” Journal Of Biomolecular Structure & Dynamics 42, no. 5 (2024): 2341–2357. 37098809 10.1080/07391102.2023.2204496

[110] A. Korniluk , O. Koper , H. Kemona , and V. Dymicka‐Piekarska , “From Inflammation to Cancer,” Irish Journal Of Medical Science 186, no. 1 (2017): 57–62. 27156054 10.1007/s11845-016-1464-0 PMC5323483

[111] Y. Z. Wu , S. Antony , J. L. Meitzler , and J. H. Doroshow , “Molecular Mechanisms Underlying Chronic Inflammation‐associated Cancers,” Cancer Letters 345, no. 2 (2014): 164–173. 23988267 10.1016/j.canlet.2013.08.014 PMC3935998

[112] H. H. Rahman , D. Niemann , and S. H. Munson‐McGee , “Association Between Asthma, Chronic Bronchitis, Emphysema, Chronic Obstructive Pulmonary Disease, and Lung Cancer in the US Population,” Environmental Science And Pollution Research 30, no. 8 (2023): 20147–20158. 36251191 10.1007/s11356-022-23631-3

[113] D. M. Ma , X. W. Dong , X. Han , et al., “Pancreatitis and Pancreatic Cancer Risk,” Technology In Cancer Research & Treatment 22 (2023): 8. 10.1177/15330338231164875 PMC10052482 36972517

[114] R. Mansilla‐Vivar , C. A. Serrano , C. Palma , et al., “High Helicobacter pylori Bacterial Load and Low Cytokine Expression Levels Are Associated With Nodular Gastropathy,” Digestive Diseases And Sciences 65, no. 2 (2020): 565–575. 31392473 10.1007/s10620-019-05769-2

[115] L. H. Jia , Y. L. Hu , G. H. Yang , and P. L. Li , “Puerarin Suppresses Cell Growth and Migration in HPV‐positive Cervical Cancer Cells by Inhibiting the PI3K/mTOR Signaling Pathway,” Experimental And Therapeutic Medicine 18, no. 1 (2019): 543–549. 31258692 10.3892/etm.2019.7589 PMC6566033

[116] Z. Wu , S. Kan , C. Li , and X. Lu , “Effect of puerarin on Apoptosis of human Hepatocellular Carcinoma Cells Under Oxidative Stress and Its Mechanisms,” J buon 24, no. 2 (2019): 628–633. 31128016

[117] X. Wang , J. Zhao , Y. Li , S. Gao , and L. Su , “Luteolin as a Multifaceted Immunomodulator: Insights Into Its Effects on Diverse Immune Cell Populations and Therapeutic Implications,” Frontiers In Immunology 16 (2025): 1621367. 41164200 10.3389/fimmu.2025.1621367 PMC12558972

[118] M. Panji , V. Behmard , Z. Zare , et al., “Suppressing Effects of Green Tea Extract and Epigallocatechin‐3‐gallate (EGCG) on TGF‐β‐ induced Epithelial‐to‐mesenchymal Transition via ROS/Smad Signaling in human Cervical Cancer Cells,” Gene 794 (2021): 145774. 34126197 10.1016/j.gene.2021.145774

[119] S. Tabassum , J. S. Ali , R. F. Saeed , et al., “Bioactivity‐Directed Isolation of Anticancer Constituents From Underexplored Folklore: Rhus punjabensis Stewart,” Molecules (Basel, Switzerland) 30, no. 22 (2025). 10.3390/molecules30224339 PMC12655016 41302399

[120] K. Y. Zhu , Y. Cai , L. Lan , and N. Luo , “Tumor Metabolic Reprogramming and Ferroptosis: The Impact of Glucose, Protein, and Lipid Metabolism,” International Journal Of Molecular Sciences 25, no. 24 (2024): 23. 10.3390/ijms252413413 PMC11676715 39769177

[121] P. Vaupel , H. Schmidberger , and A. Mayer , “The Warburg Effect: Essential Part of Metabolic Reprogramming and central Contributor to Cancer Progression,” International Journal Of Radiation Biology 95, no. 7 (2019): 912–919. 30822194 10.1080/09553002.2019.1589653

[122] X. H. Guo , S. S. Jiang , L. L. Zhang , et al., “Berberine Exerts Its Antineoplastic Effects by Reversing the Warburg Effect via Downregulation of the Akt/mTOR/GLUT1 Signaling Pathway,” Oncology Reports 46, no. 6 (2021): 253. 34643248 10.3892/or.2021.8204 PMC8548812

[123] L. Jia , S. Huang , X. Yin , et al., “Quercetin Suppresses the Mobility of Breast Cancer by Suppressing Glycolysis Through Akt‐mTOR Pathway Mediated Autophagy Induction,” Life Sciences 208 (2018): 123–130. 30025823 10.1016/j.lfs.2018.07.027

[124] Y. Zhao , W. Lai , Y. Xu , et al., “Exogenous and Endogenous Therapeutic Effects of Combination Sodium Ferulate and Bone Marrow Stromal Cells (BMSCs) Treatment Enhance Neurogenesis After Rat Focal Cerebral Ischemia,” Metabolic Brain Disease 28, no. 4 (2013): 655–666. 23955489 10.1007/s11011-013-9425-z

[125] T. Zhan , M. Digel , E. M. Küch , W. Stremmel , and J. Füllekrug , “Silybin and Dehydrosilybin Decrease Glucose Uptake by Inhibiting GLUT Proteins,” Journal Of Cellular Biochemistry 112, no. 3 (2011): 849–859. 21328458 10.1002/jcb.22984

[126] Y. J. Tan , A. Ali , S. Y. Tee , et al., “Galloyl Esters of Trans‐stilbenes Are Inhibitors of FASN With Anticancer Activity on Non‐small Cell Lung Cancer Cells,” European Journal Of Medicinal Chemistry 182 (2019): 111597. 31422225 10.1016/j.ejmech.2019.111597

[127] M. C. Nocito , P. Avena , L. Zavaglia , et al., “Adrenocortical Carcinoma (ACC) Cells Rewire Their Metabolism to Overcome Curcumin Antitumoral Effects Opening a Window of Opportunity to Improve Treatment,” Cancers (Basel) 15, no. 4 (2023): 1050. 36831394 10.3390/cancers15041050 PMC9954484

[128] M. Shi , Y. Mobet , and H. Shen , “Quercetin Attenuates Acute Kidney Injury Caused by Cisplatin by Inhibiting Ferroptosis and Cuproptosis,” Cell Biochemistry AOnd Biophysics 82, no. 3 (2024): 2687–2699. 10.1007/s12013-024-01379-6 39026057

[129] A. Shahzad , W. J. Liu , Y. J. Sun , et al., “Flavonoids as Modulators of Metabolic Reprogramming in Renal Cell Carcinoma (Review),” Oncology Reports 52, no. 6 (2024): 17. 10.3892/or.2024.8826 PMC11526433 39422066

[130] M. Samec , A. Mazurakova , V. Lucansky , et al., “Flavonoids Attenuate Cancer Metabolism by Modulating Lipid Metabolism, Amino Acids, Ketone Bodies and Redox state Mediated by Nrf2,” European Journal Of Pharmacology 949 (2023): 19. 10.1016/j.ejphar.2023.175655 36921709

[131] N. Shen , T. F. Wang , Q. Gan , et al., “Plant Flavonoids: Classification, Distribution, Biosynthesis, and Antioxidant Activity,” Food Chemistry 383 (2022): 13. 10.1016/j.foodchem.2022.132531 35413752

[132] J. W. Kim , A. R. Amin , and D. M. Shin , “Chemoprevention of Head and Neck Cancer With Green Tea Polyphenols,” Cancer Prev Res (Philadelphia, Pa) 3, no. 8 (2010): 900–909. 10.1158/1940-6207.CAPR-09-0131 PMC2917478 20663981

[133] S. Venturelli , M. Burkard , M. Biendl , et al., “Prenylated Chalcones and Flavonoids for the Prevention and Treatment of Cancer,” Nutrition (Burbank, Los Angeles County, Calif) 32, no. 11‐12 (2016): 1171–1178. 27238957 10.1016/j.nut.2016.03.020

[134] G. D. Kim , “Kaempferol Inhibits Angiogenesis by Suppressing HIF‐1α and VEGFR2 Activation via ERK/p38 MAPK and PI3K/Akt/mTOR Signaling Pathways in Endothelial Cells,” Prev Nutr Food Sci 22, no. 4 (2017): 320–326. 29333385 10.3746/pnf.2017.22.4.320 PMC5758096

[135] J. Chen , A. Y. Chen , H. Huang , et al., “The Flavonoid Nobiletin Inhibits Tumor Growth and Angiogenesis of Ovarian Cancers via the Akt Pathway,” International Journal Of Oncology 46, no. 6 (2015): 2629–2638. 25845666 10.3892/ijo.2015.2946 PMC4441297

[136] X. Li , Z. S. Zhang , X. H. Zhang , et al., “Cyanidin Inhibits EMT Induced by Oxaliplatin via Targeting the PDK1‐PI3K/Akt Signaling Pathway,” Food Funct 10, no. 2 (2019): 592–601. 30672917 10.1039/c8fo01611a

[137] Y. Chen , S. Wang , B. Geng , and Z. Yi , “Pelargonidin Induces Antitumor Effects in human Osteosarcoma Cells via Autophagy Induction, Loss of Mitochondrial Membrane Potential, G2/M Cell Cycle Arrest and Downregulation of PI3K/AKT Signalling Pathway,” J buon 23, no. 3 (2018): 735–740. 30003744

[138] C. Martinez‐Perez , C. Ward , G. Cook , et al., “Novel Flavonoids as Anti‐cancer Agents: Mechanisms of Action and Promise for Their Potential Application in Breast Cancer,” Biochemical Society Transactions 42, no. 4 (2014): 1017–1023. 25109996 10.1042/BST20140073

[139] H. W. Wu , H. Huang , and Y. M. Zhao , “Interplay Between Metabolic Reprogramming and Post‐translational Modifications: From Glycolysis to Lactylation,” Frontiers In Immunology 14 (2023): 12. 10.3389/fimmu.2023.1211221 PMC10338923 37457701

[140] S. Daneshvar , M. Y. Zamanian , M. S. Ivraghi , et al., “A Comprehensive View on the Apigenin Impact on Colorectal Cancer: Focusing on Cellular and Molecular Mechanisms,” Food Sci Nutr 11, no. 11 (2023): 6789–6801. 37970406 10.1002/fsn3.3645 PMC10630840

[141] J. H. Han , M. Kim , H. J. Kim , et al., “Targeting Lactate Dehydrogenase A With Catechin Resensitizes SNU620/5FU Gastric Cancer Cells to 5‐Fluorouracil,” International Journal Of Molecular Sciences 22, no. 10 (2021): 5406. 34065602 10.3390/ijms22105406 PMC8161398

[142] H. S. Kim , T. Wannatung , S. Lee , et al., “Quercetin Enhances Hypoxia‐mediated Apoptosis via Direct Inhibition of AMPK Activity in HCT116 Colon Cancer,” Apoptosis 17, no. 9 (2012): 938–949. 22684842 10.1007/s10495-012-0719-0

[143] J. W. Gu , K. L. Makey , K. B. Tucker , et al., “EGCG, a Major Green Tea Catechin Suppresses Breast Tumor Angiogenesis and Growth via Inhibiting the Activation of HIF‐1α and NFκB, and VEGF Expression,” Vasc Cell 5, no. 1 (2013): 9. 23638734 10.1186/2045-824X-5-9 PMC3649947

[144] J. Y. Yao , S. Xu , Y. N. Sun , et al., “Novel CDK9 Inhibitor Oroxylin A Promotes Wild‐type P53 Stability and Prevents Hepatocellular Carcinoma Progression by Disrupting both MDM2 and SIRT1 Signaling,” Acta Pharmacologica Sinica 43, no. 4 (2022): 1033–1045. 34188177 10.1038/s41401-021-00708-2 PMC8975870

[145] H. Cheng , M. Wang , J. J. Su , et al., “Lipid Metabolism and Cancer,” Life‐Basel 12, no. 6 (2022): 34. 10.3390/life12060784 PMC9224822 35743814

[146] R. M. Rifaat , M. W. Kamel , M. Sharaky , Y. M. Attia , and S. A. Shouman , “Targeting Lipid Metabolism to Overcome Tamoxifen Resistance in Breast Cancer: Evaluating the Synergistic Therapeutic Potential of Quercetin,” Cancer Treat Res Commun 44 (2025): 100953. 40554412 10.1016/j.ctarc.2025.100953

[147] S. Gómez‐Zorita , A. Lasa , N. Abendaño , et al., “Phenolic Compounds Apigenin, Hesperidin and Kaempferol Reduce in Vitro Lipid Accumulation in human Adipocytes,” Journal Of Translational Medicine 15, no. 1 (2017): 237. 29162103 10.1186/s12967-017-1343-0 PMC5696737

[148] D. T. Coleman , R. Bigelow , and J. A. Cardelli , “Inhibition of Fatty Acid Synthase by Luteolin Post‐transcriptionally Down‐regulates c‐Met Expression Independent of Proteosomal/Lysosomal Degradation,” Molecular Cancer Therapeutics 8, no. 1 (2009): 214–224. 19139131 10.1158/1535-7163.MCT-08-0722 PMC2741738

[149] K. Yang , G. Zhu , T. Peng , Y. Cheng , and X. Guo , “FASN Regulates CSE‐induced Apoptosis, Oxidative Stress and Mitochondrial Damage in Type 2 Alveolar Epithelial Cells by Regulating NRF2 Expression and Nuclear Translocation,” Redox Report: Communications In Free Radical Research 30, no. 1 (2025): 2550412. 41025365 10.1080/13510002.2025.2550412 PMC12486463

[150] Z. M. Zhang , X. Y. Yang , J. H. Yuan , Z. Y. Sun , and Y. Q. Li , “Modulation of NRF2 and UGT1A Expression by Epigallocatechin‐3‐gallate in Colon Cancer Cells and BALB/c Mice,” Chinese Medical Journal 122, no. 14 (2009): 1660–1665. 19719968

[151] N. S. Yarla , A. Bishayee , G. Sethi , et al., “Targeting Arachidonic Acid Pathway by Natural Products for Cancer Prevention and Therapy,” Seminars n Cancer Biology (2016): 48–81. 40‐41. 10.1016/j.semcancer.2016.02.001 26853158

[152] C. Monmai and S. H. Baek , “Anti‐Inflammatory Effects of the Combined Treatment of Resveratrol‐ and Protopanaxadiol‐Enriched Rice Seed Extract on Lipopolysaccharide‐Stimulated RAW264.7 Cells,” Molecules (Basel, Switzerland) 29, no. 18 (2024). 10.3390/molecules29184343 PMC11434488 39339339

[153] S. Pisonero‐Vaquero , Á. Martínez‐Ferreras , M. V. García‐Mediavilla , et al., “Quercetin Ameliorates Dysregulation of Lipid Metabolism Genes via the PI3K/AKT Pathway in a Diet‐induced Mouse Model of Nonalcoholic Fatty Liver Disease,” Molecular Nutrition & Food Research 59, no. 5 (2015): 879–893. 25712622 10.1002/mnfr.201400913

[154] S. Nimmagadda , K. Glunde , M. G. Pomper , and Z. M. Bhujwalla , “Pharmacodynamic Markers for Choline Kinase Down‐regulation in Breast Cancer Cells,” Neoplasia 11, no. 5 (2009): 477–484. 19412432 10.1593/neo.81430 PMC2671858

[155] D. Nan , W. P. Yao , L. L. Huang , et al., “Glutamine and Cancer: Metabolism, Immune Microenvironment, and Therapeutic Targets,” Cell Communication And Signaling 23, no. 1 (2025): 17. 39856712 10.1186/s12964-024-02018-6 PMC11760113

[156] Z. Wei , S. Ye , H. Feng , et al., “Silybin Suppresses Ovarian Cancer Cell Proliferation by Inhibiting Isocitrate Dehydrogenase 1 Activity,” Cancer Science 113, no. 9 (2022): 3032–3043. 35730256 10.1111/cas.15470 PMC9459272

[157] J. Li , D. Zhang , S. Wang , et al., “Baicalein Induces Apoptosis by Inhibiting the Glutamine‐mTOR Metabolic Pathway in Lung Cancer,” Journal Of Advanced Research 68 (2025): 341–357. 38432394 10.1016/j.jare.2024.02.023 PMC11785570

[158] K. Wang , W. Zhang , Z. Wang , et al., “Flavokawain A Inhibits Prostate Cancer Cells by Inducing Cell Cycle Arrest and Cell Apoptosis and Regulating the Glutamine Metabolism Pathway,” Journal Of Pharmaceutical And Biomedical Analysis 186 (2020): 113288. 32361091 10.1016/j.jpba.2020.113288

[159] L. Zhexian , G. Xingqi , D. Xinxin , et al., “Clinical Application Prospects of Traditional Chinese Medicine as Adjuvant Therapy for Metabolic Reprogramming in Colorectal Cancer,” Frontiers In Immunology 16 (2025): 1630279. 40735327 10.3389/fimmu.2025.1630279 PMC12303939

[160] H. Y. Wu , L. L. Pan , C. X. Gao , et al., “Quercetin Inhibits the Proliferation of Glycolysis‐Addicted HCC Cells by Reducing Hexokinase 2 and Akt‐mTOR Pathway,” Molecules (Basel, Switzerland) 24, no. 10 (2019): 11. 10.3390/molecules24101993 PMC6572074 31137633

[161] L. J. Jia , S. Huang , X. R. Yin , et al., “Quercetin Suppresses the Mobility of Breast Cancer by Suppressing Glycolysis Through Akt‐mTOR Pathway Mediated Autophagy Induction,” Life Sciences 208 (2018): 123–130. 30025823 10.1016/j.lfs.2018.07.027

[162] S. H. Shan , J. Y. Shi , P. Yang , et al., “Apigenin Restrains Colon Cancer Cell Proliferation via Targeted Blocking of Pyruvate Kinase M2‐Dependent Glycolysis,” Journal Of Agricultural And Food Chemistry 65, no. 37 (2017): 8136–8144. 28829588 10.1021/acs.jafc.7b02757

[163] J. Yuan , G. Peng , G. L. Xiao , et al., “Xanthohumol Suppresses Glioblastoma via Modulation of Hexokinase 2‐mediated Glycolysis,” Journal Of Cancer 11, no. 14 (2020): 4047–4058. 32368287 10.7150/jca.33045 PMC7196271

[164] X. K. Zheng , Y. Y. Ke , A. Z. Feng , et al., “The Mechanism by Which Amentoflavone Improves Insulin Resistance in HepG2 Cells,” Molecules (Basel, Switzerland) 21, no. 5 (2016): 13. 10.3390/molecules21050624 PMC6274486 27187341

[165] Z. Tai , Y. Lin , Y. He , et al., “Luteolin Sensitizes the Antiproliferative Effect of Interferon α/β by Activation of Janus Kinase/Signal Transducer and Activator of Transcription Pathway Signaling Through Protein Kinase A‐mediated Inhibition of Protein Tyrosine Phosphatase SHP‐2 in Cancer Cells,” Cell Signalling 26, no. 3 (2014): 619–628. 24333668 10.1016/j.cellsig.2013.11.039

[166] D. Venkatesh , S. Sarkar , T. Kandasamy , and S. S. Ghosh , “In‐silico Identification and Validation of Silibinin as a Dual Inhibitor for ENO1 and GLUT4 to Curtail EMT Signaling and TNBC Progression,” Computational Biology And Chemistry 115 (2025): 108312. 39689434 10.1016/j.compbiolchem.2024.108312

[167] M. Ashrafizadeh , M. R. Bakhoda , Z. Bahmanpour , et al., “Apigenin as Tumor Suppressor in Cancers: Biotherapeutic Activity, Nanodelivery, and Mechanisms with Emphasis on Pancreatic Cancer,” Front Chem 8 (2020): 829. 33195038 10.3389/fchem.2020.00829 PMC7593821

[168] J. Wei , G. Lei , Q. Chen , et al., “Casticin Inhibits Proliferation of Non‐small Cell Lung Cancer Cells Through Regulating Reprogramming of Glucose Metabolism,” Phytomedicine 136 (2025): 156278. 39647464 10.1016/j.phymed.2024.156278

[169] L. Wang , M. Liu , F. Yin , et al., “Trilobatin, a Novel SGLT1/2 Inhibitor, Selectively Induces the Proliferation of Human Hepatoblastoma Cells,” Molecules (Basel, Switzerland) 24, no. 18 (2019): 3390. 31540429 10.3390/molecules24183390 PMC6767144

[170] Y. Li , D. Kong , A. Ahmad , et al., “Epigenetic Deregulation of miR‐29a and miR‐1256 by Isoflavone Contributes to the Inhibition of Prostate Cancer Cell Growth and Invasion,” Epigenetics 7, no. 8 (2012): 940–949. 22805767 10.4161/epi.21236 PMC3427289

[171] Z. Wang , D. Wang , S. Han , et al., “Bioactivity‐guided Identification and Cell Signaling Technology to Delineate the Lactate Dehydrogenase A Inhibition Effects of Spatholobus Suberectus on Breast Cancer,” PLoS ONE 8, no. 2 (2013): e56631. 23457597 10.1371/journal.pone.0056631 PMC3572989

[172] J. D. Fu , J. J. Yao , H. Wang , et al., “Effects of EGCG on Proliferation and Apoptosis of Gastric Cancer SGC7901 Cells via Down‐regulation of HIF‐1α and VEGF Under a Hypoxic state,” European Review For Medical And Pharmacological Sciences 23, no. 1 (2019): 155–161. 30657557 10.26355/eurrev_201901_16759

[173] F. Chen , M. Zhuang , C. Zhong , et al., “Baicalein Reverses Hypoxia‐induced 5‐FU Resistance in Gastric Cancer AGS Cells Through Suppression of Glycolysis and the PTEN/Akt/HIF‐1α Signaling Pathway,” Oncology Reports 33, no. 1 (2015): 457–463. 25333894 10.3892/or.2014.3550

[174] Q. Liu , X. Chen , Y. Tan , et al., “Natural Products as Glycolytic Inhibitors for Cervical Cancer Treatment: A Comprehensive Review,” Biomedicine & Pharmacotherapy 175 (2024): 116708. 38723515 10.1016/j.biopha.2024.116708

[175] D. Jia , C. Liu , Z. Zhu , et al., “Novel Transketolase Inhibitor Oroxylin A Suppresses the Non‐oxidative Pentose Phosphate Pathway and Hepatocellular Carcinoma Tumour Growth in Mice and Patient‐derived Organoids,” Clinical And Translational Medicine 12, no. 11 (2022): e1095. 36314067 10.1002/ctm2.1095 PMC9619225

[176] K. Toshida , S. Itoh , N. Iseda , et al., “The Impact of TP53‐Induced Glycolysis and Apoptosis Regulator on Prognosis in Hepatocellular Carcinoma: Association With Tumor Microenvironment and Ferroptosis,” Liver Cancer 14, no. 1 (2025): 36–57. 40144470 10.1159/000540180 PMC11936447

[177] P. Giridharan , S. T. Somasundaram , K. Perumal , et al., “Novel Substituted Methylenedioxy Lignan Suppresses Proliferation of Cancer Cells by Inhibiting Telomerase and Activation of c‐myc and Caspases Leading to Apoptosis,” British Journal Of Cancer 87, no. 1 (2002): 98–105. 12085264 10.1038/sj.bjc.6600422 PMC2364294

[178] M. Alam , S. Ali , G. M. Ashraf , et al., “Epigallocatechin 3‐gallate: From Green Tea to Cancer Therapeutics,” Food Chemistry 379 (2022): 132135. 35063850 10.1016/j.foodchem.2022.132135

[179] Y. Zhou , L. Chen , D. Ding , et al., “Cyanidin‐3‐O‐glucoside Inhibits the β‐catenin/MGMT Pathway by Upregulating miR‐214‐5p to Reverse Chemotherapy Resistance in Glioma Cells,” Scientific Reports 12, no. 1 (2022): 7773. 35545654 10.1038/s41598-022-11757-w PMC9095653

[180] J. Cao , X. Ma , X. Yan , et al., “Kaempferol Induces Mitochondrial Dysfunction and Mitophagy by Activating the LKB1/AMPK/MFF Pathway in Breast Precancerous Lesions,” Phytotherapy Research 37, no. 8 (2023): 3602–3616. 37086359 10.1002/ptr.7838

[181] A. Guo , Y. Chang , J. Lin , et al., “Resveratrol Enhances Anticancer Effects of Silybin on HepG2 Cells and H22 Tumor‐bearing Mice via Inducing G2/M Phase Arrest and Increasing Bax/Bcl‐2 Ratio,” Combinatorial Chemistry & High Throughput Screening 28, no. 1 (2025): 89–98. 38204247 10.2174/0113862073263408231101105647

[182] T. Puig , A. Vázquez‐Martín , J. Relat , et al., “Fatty Acid Metabolism in Breast Cancer Cells: Differential Inhibitory Effects of Epigallocatechin Gallate (EGCG) and C75,” Breast Cancer Research And Treatment 109, no. 3 (2008): 471–479. 17902053 10.1007/s10549-007-9678-5

[183] K. Y. Jang , S. J. Jeong , S. H. Kim , et al., “Activation of Reactive Oxygen Species/AMP Activated Protein Kinase Signaling Mediates Fisetin‐induced Apoptosis in Multiple Myeloma U266 Cells,” Cancer Letters 319, no. 2 (2012): 197–202. 22261340 10.1016/j.canlet.2012.01.008

[184] D. Hess and R. A. Igal , “Genistein Downregulates De Novo Lipid Synthesis and Impairs Cell Proliferation in human Lung Cancer Cells,” Experimental Biology And Medicine (Maywood, NJ) 236, no. 6 (2011): 707–713. 10.1258/ebm.2011.010265 21565896

[185] J. Li , T. Wang , P. Liu , et al., “Hesperetin Ameliorates Hepatic Oxidative Stress and Inflammation via the PI3K/AKT‐Nrf2‐ARE Pathway in Oleic Acid‐induced HepG2 Cells and a Rat Model of High‐fat Diet‐induced NAFLD,” Food Funct 12, no. 9 (2021): 3898–3918. 33977953 10.1039/d0fo02736g

[186] N. M. Borradaile , L. E. de Dreu , P. H. Barrett , and M. W. Huff , “Inhibition of Hepatocyte apoB Secretion by Naringenin: Enhanced Rapid Intracellular Degradation Independent of Reduced Microsomal Cholesteryl Esters,” Journal Of Lipid Research 43, no. 9 (2002): 1544–1554. 12235187 10.1194/jlr.m200115-jlr200

[187] W. Jäger , A. Gruber , B. Giessrigl , et al., “Metabolomic Analysis of Resveratrol‐induced Effects in the human Breast Cancer Cell Lines MCF‐7 and MDA‐MB‐231,” Omics 15, no. 1‐2 (2011): 9–14. 21241168 10.1089/omi.2010.0114

[188] X. Y. Wang , L. Dai , Y. Liu , and G. Li , “Untargeted Metabolomic Analysis of the Therapeutic Effects of Pholiota Adiposa in H22 Hepatocellular Carcinoma Tumor‐bearing Mice,” Journal Of Bioenergetics And Biomembranes 57, no. 4‐5 (2025): 315–335. 40772996 10.1007/s10863-025-10070-1

[189] F. Z. Mokhfi , M. Al Amin , M. Zehravi , et al., “Alkaloid‐based Modulators of the PI3K/Akt/mTOR Pathway for Cancer Therapy: Understandings From Pharmacological Point of View,” Chemico‐Biological Interactions 402 (2024): 15. 10.1016/j.cbi.2024.111218 39209016

[190] W. Zhou , A. Asif , C. Situ , J. Wang , and H. Hao , “Multiple Target and Regulatory Pathways of berberine,” Phytomedicine 146 (2025): 157030. 40763599 10.1016/j.phymed.2025.157030

[191] L. Cheng , C. R. Wu , L. H. Zhu , H. Li , and L. X. Chen , “Physapubescin, a Natural Withanolide as a Kidney‐type Glutaminase (KGA) Inhibitor,” Bioorganic & Medicinal Chemistry Letters 27, no. 5 (2017): 1243–1246. 28174105 10.1016/j.bmcl.2017.01.057

[192] W. H. Zhu , W. Zhang , and N. Xu , “Dihydroartemisinin Induces Apoptosis and Downregulates Glucose Metabolism in JF‐305 Pancreatic Cancer Cells,” Rsc Advances 8, no. 37 (2018): 20692–20700. 35542352 10.1039/c8ra00565f PMC9080833

[193] S. Fakhri , S. Z. Moradi , S. Y. Moradi , et al., “Phytochemicals Regulate Cancer Metabolism Through Modulation of the AMPK/PGC‐1α Signaling Pathway,” BMC cancer 24, no. 1 (2024): 29. 39223494 10.1186/s12885-024-12715-7 PMC11368033

[194] R. Yang , X. L. Fang , Q. Zhen , Q. Y. Chen , and C. J. Feng , “Mitochondrial Targeting Nano‐curcumin for Attenuation on PKM2 and FASN,” Colloids And Surfaces B‐Biointerfaces 182 (2019): 7. 10.1016/j.colsurfb.2019.110405 31377611

[195] S. W. Kim , M. J. Cha , S. K. Lee , et al., “Curcumin Treatment in Combination With Glucose Restriction Inhibits Intracellular Alkalinization and Tumor Growth in Hepatoma Cells,” International Journal Of Molecular Sciences 20, no. 10 (2019): 17. 10.3390/ijms20102375 PMC6566721 31091659

[196] R. Wei , L. M. Mao , P. Xu , et al., “Suppressing Glucose Metabolism With Epigallocatechin‐3‐gallate (EGCG) Reduces Breast Cancer Cell Growth in Preclinical Models,” Food & Function 9, no. 11 (2018): 5682–5696. 30310905 10.1039/c8fo01397g PMC7480214

[197] M. Puxeddu , R. Silvestri , and G. L. Regina , “Metabolism, a Blossoming Target for Small‐Molecule Anticancer Drugs,” Molecules (Basel, Switzerland) 30, no. 17 (2025): 39. 10.3390/molecules30173457 PMC12430568 40941984

[198] D. Uogintaite , R. Baniene , A. Jasukaitiene , et al., “Effect of Fireweed (Chamerion angustifolium L.) Extracts and Oenothein B on Colon Cancer Cells: Impact of Leaf Fermentation on Viability and Mitochondrial Function,” Medicina (Kaunas, Lithuania) 61, no. 11 (2025): 1957. 41303794 10.3390/medicina61111957 PMC12654776

[199] A. Chauhan , S. Dewali , V. M. Pathak , et al., “Therapeutic Role of Allicin in Gastrointestinal Cancers: Mechanisms and Safety Aspects,” Discov Oncol 16, no. 1 (2025): 1731. 41003866 10.1007/s12672-025-02591-3 PMC12474849

[200] I. Debnath and M. Kundu , “Therapeutic Potential of Natural Compounds in Targeting Cancer Stem Cells: A Promising Approach for Cancer Treatment,” Discov Oncol 16, no. 1 (2025): 1433. 40721907 10.1007/s12672-025-03190-y PMC12304396

[201] E. M. Kamel , D. A. Abdelrheem , S. I. Othman , S. Maodaa , and A. M. Lamsabhi , “A Multifaceted Mechanistic Approach to Assess the Inhibitory Potential of Broccoli‐Derived Glucosinolates against Tumor‐Associated Carbonic Anhydrase IX,” Arch Pharm (Weinheim) 358, no. 6 (2025): e70019. 40544485 10.1002/ardp.70019

[202] K. Iwar , K. Ochar , Y. A. Seo , B. K. Ha , and S. H. Kim , “Alliums as Potential Antioxidants and Anticancer Agents,” International Journal Of Molecular Sciences 25, no. 15 (2024): 8079. 39125648 10.3390/ijms25158079 PMC11312234

[203] D. Lu , L. Yuan , G. Chen , et al., “Exploring the Effect of Huangqi Fuling Decoction on Gastric Cancer Based on UPLC‐MS, Network Pharmacology and Experiments in Vitro,” BMC Complement Med Ther 25, no. 1 (2025): 405. 41184845 10.1186/s12906-025-05111-6 PMC12581539

[204] B. Arneth , “Tumor Microenvironment,” Medicina‐Lithuania 56, no. 1 (2020): 21. 10.3390/medicina56010015 PMC7023392 31906017

[205] J. J. Wang , K. F. Lei , and F. Han , “Tumor Microenvironment: Recent Advances in Various Cancer Treatments,” European Review For Medical And Pharmacological Sciences 22, no. 12 (2018): 3855–3864. 29949179 10.26355/eurrev_201806_15270

[206] W. L. Zhang , S. B. Li , C. T. Li , T. Y. Li , and Y. Y. Huang , “Remodeling Tumor Microenvironment With Natural Products to Overcome Drug Resistance,” Frontiers In Immunology 13 (2022): 22. 10.3389/fimmu.2022.1051998 PMC9685561 36439106

[207] R. A. Glabman , P. L. Choyke , and N. Sato , “Cancer‐Associated Fibroblasts: Tumorigenicity and Targeting for Cancer Therapy,” Cancers 14, no. 16 (2022): 21. 10.3390/cancers14163906 PMC9405783 36010899

[208] C. Li , X. Wang , L. Xing , et al., “Huaier‐induced Suppression of Cancer‐associated Fibroblasts Confers Immunotherapeutic Sensitivity in Triple‐negative Breast Cancer,” Phytomedicine 135 (2024): 156051. 39299097 10.1016/j.phymed.2024.156051

[209] P. Dauer , X. Zhao , V. K. Gupta , et al., “Inactivation of Cancer‐Associated‐Fibroblasts Disrupts Oncogenic Signaling in Pancreatic Cancer Cells and Promotes Its Regression,” Cancer Research 78, no. 5 (2018): 1321–1333. 29259015 10.1158/0008-5472.CAN-17-2320 PMC5935584

[210] C. Guirong , H. Lili , D. Guifang , et al., “Qingre Huayu Jianpi Decoction Suppresses Colorectal Tumorigenesis by Inhibiting Cancer Stem Cells and Cancer‐associated Fibroblasts,” Journal Of Ethnopharmacology 347 (2025): 119722. 40220938 10.1016/j.jep.2025.119722

[211] X. H. Yao and Y. Zeng , “Tumour Associated Endothelial Cells: Origin, Characteristics and Role in Metastasis and Anti‐angiogenic Resistance,” Frontiers In Physiology 14 (2023): 8. 10.3389/fphys.2023.1199225 PMC10301839 37389120

[212] Z. P. Fu , X. Chen , S. W. Guan , et al., “Curcumin Inhibits Angiogenesis and Improves Defective Hematopoiesis Induced by Tumor‐derived VEGF in Tumor Model Through Modulating VEGF‐VEGFR2 Signaling Pathway,” Oncotarget 6, no. 23 (2015): 19469–19482. 26254223 10.18632/oncotarget.3625 PMC4637299

[213] Q. Zhang , J. Liu , H. Duan , et al., “Activation of Nrf2/HO‐1 Signaling: An Important Molecular Mechanism of Herbal Medicine in the Treatment of Atherosclerosis via the Protection of Vascular Endothelial Cells From Oxidative Stress,” Journal Of Advanced Research 34 (2021): 43–63. 35024180 10.1016/j.jare.2021.06.023 PMC8655139

[214] Y. R. Wang , B. Wang , M. Guerram , et al., “Deoxypodophyllotoxin Suppresses Tumor Vasculature in HUVECs by Promoting Cytoskeleton Remodeling Through LKB1‐AMPK Dependent Rho A Activation,” Oncotarget 6, no. 30 (2015): 29497–29512. 26470595 10.18632/oncotarget.4985 PMC4745742

[215] Z. Li , M. Huang , J. Wang , et al., “Betulin, an Active Component From Chinese Herb Birch Bark, Suppresses Tumor Angiogenesis and Tumor Growth by Inhibiting the PAX2/VEGF‐A/VEGR2 Signaling Pathway in Non‐small Cell Lung Cancer,” Phytomedicine 148 (2025): 157445. 41166909 10.1016/j.phymed.2025.157445

[216] J. S. Di Martino , T. Akhter , and J. J. Bravo‐Cordero , “Remodeling the ECM: Implications for Metastasis and Tumor Dormancy,” Cancers 13, no. 19 (2021): 12. 10.3390/cancers13194916 PMC8507703 34638400

[217] C. Lou , Z. Zhu , Y. Zhao , R. Zhu , and H. Zhao , “Arctigenin, a Lignan From Arctium Lappa L., Inhibits Metastasis of human Breast Cancer Cells Through the Downregulation of MMP‐2/‐9 and Heparanase in MDA‐MB‐231 Cells,” Oncology Reports 37, no. 1 (2017): 179–184. 27878294 10.3892/or.2016.5269

[218] H. Y. Du , M. Olivo , R. Mahendran , et al., “Hypericin Photoactivation Triggers Down‐regulation of Matrix Metalloproteinase‐9 Expression in Well‐differentiated human Nasopharyngeal Cancer Cells,” Cellular And Molecular Life Sciences 64, no. 7‐8 (2007): 979–988. 17385073 10.1007/s00018-007-7030-1 PMC11136193

[219] P. Song , F. Song , T. T. Shao , et al., “Natural Products: Promising Therapeutics for Targeting Regulatory Immune Cells in the Tumor Microenvironment,” Frontiers In Pharmacology 15 (2024): 23. 10.3389/fphar.2024.1481850 PMC11598344 39605905

[220] Z. C. Dongye , X. P. Wu , Y. X. Wen , et al., “Icaritin and Intratumoral Injection of CpG Treatment Synergistically Promote T Cell Infiltration and Antitumor Immune Response in Mice,” International Immunopharmacology 111 (2022): 12. 10.1016/j.intimp.2022.109093 35930912

[221] S. M. Qiao , C. J. Lv , Y. Tao , et al., “Arctigenin Disrupts NLRP3 Inflammasome Assembly in Colonic Macrophages via Downregulating Fatty Acid Oxidation to Prevent Colitis‐associated Cancer,” Cancer Letters 491 (2020): 162–179. 32861708 10.1016/j.canlet.2020.08.033

[222] R. Yao , Y. Zhang , W. Yu , et al., “Jianpi‐huayu Decotion Regulates TREM1/DAP12 Pathway to Improve the Immunosuppressive Tumor Microenvironment and Enhance the Anti‐hepatocellular Carcinoma Effect of PD‐1 Inhibitors,” Journal Of Ethnopharmacology 356 (2026): 120846. 41177238 10.1016/j.jep.2025.120846

[223] H. Zhi , H. Fu , Y. Zhang , et al., “Progress of cGAS‐STING Signaling Pathway‐based Modulation of Immune Response by Traditional Chinese Medicine in Clinical Diseases,” Frontiers In Immunology 15 (2024): 1510628. 39737190 10.3389/fimmu.2024.1510628 PMC11683013

[224] J. L. Huo , W. J. Fu , Z. H. Liu , et al., “Research Advance of Natural Products in Tumor Immunotherapy,” Frontiers In Immunology 13 (2022): 9. 10.3389/fimmu.2022.972345 PMC9494295 36159787

[225] H. Gao , N. Kang , C. Hu , et al., “Ginsenoside Rb1 Exerts Anti‐inflammatory Effects in Vitro and in Vivo by Modulating Toll‐Like Receptor 4 Dimerization and NF‐kB/MAPKs Signaling Pathways,” Phytomedicine 69 (2020): 153197. 32146298 10.1016/j.phymed.2020.153197

[226] T. Hayashi , K. Fujita , M. Matsushita , and N. Nonomura , “Main Inflammatory Cells and Potentials of Anti‐Inflammatory Agents in Prostate Cancer,” Cancers (Basel) 11, no. 8 (2019): 1153. 31408948 10.3390/cancers11081153 PMC6721573

[227] Z. Su , Y. Li , Z. Zhou , et al., “Herbal Medicine for Colorectal Cancer Treatment: Molecular Mechanisms and Clinical Applications,” Cell Proliferation 58, no. 10 (2025): e70065. 40488304 10.1111/cpr.70065 PMC12508688

[228] X. Nie , L. Fu , Y. F. Cheng , et al., “Garcinone E Suppresses Breast Cancer Growth and Metastasis by Modulating Tumor‐associated Macrophages Polarization via STAT6 Signaling,” Phytotherapy Research 37, no. 10 (2023): 4442–4456. 37259475 10.1002/ptr.7909

[229] X. X. Deng , Y. N. Jiao , H. F. Hao , et al., “Taraxacum Mongolicum Extract Inhibited Malignant Phenotype of Triple‐negative Breast Cancer Cells in Tumor‐associated Macrophages Microenvironment Through Suppressing IL‐10 /STAT3 / PD‐L1 Signaling Pathways,” Journal f Ethnopharmacology 274 (2021): 113978. 10.1016/j.jep.2021.113978 33716082

[230] J. Xie , X. Du , Y. Li , et al., “Berberine Shaping the Tumor Immune Landscape via Pyroptosis,” Cellular Immunology 408 (2025): 104908. 39701005 10.1016/j.cellimm.2024.104908

[231] L. Li , M. Zhang , T. Liu , et al., “Quercetin‐ferrum Nanoparticles Enhance Photothermal Therapy by Modulating the Tumor Immunosuppressive Microenvironment,” Acta Biomaterialia 154 (2022): 454–466. 36243377 10.1016/j.actbio.2022.10.008

[232] W. Hu , Y. Ma , Q. Zhang , et al., “DCs‐targeted Full‐cell Nanovaccine Based on Chitosan Oligosaccharide Self‐assembled With Ganoderma Lucidum Polysaccharide for Enhancing Tumor Immunotherapy,” International Journal Of Biological Macromolecules 334, no. Pt 1 (2025): 148934. 41253106 10.1016/j.ijbiomac.2025.148934

[233] Y. B. Feng , L. Chen , F. X. Chen , et al., “Immunopotentiation Effects of Apigenin on NK Cell Proliferation and Killing Pancreatic Cancer Cells,” International Journal Of Immunopathology And Pharmacology 37 (2023): 3946320231161174. 36848930 10.1177/03946320231161174 PMC9974612

[234] J. Gnanagurusamy , R. Sabanayagam , S. Krishnamoorthy , V. Balasubramanian , and S. Muthusami , “EGCG Remodels the TGF‐β Cervical Cancer Micro‐environment towards Immune Responsiveness,” Cellular Immunology 418 (2025): 105027. 41005217 10.1016/j.cellimm.2025.105027

[235] C. J. Wang , C. Wang , J. Han , et al., “Effect of Combined Treatment With Recombinant Interleukin‐2 and Allicin on Pancreatic Cancer,” Molecular Biology Reports 40, no. 12 (2013): 6579–6585. 24135803 10.1007/s11033-013-2766-1

[236] L. O. Afolabi , J. C. Bi , L. Chen , and X. C. Wan , “A Natural Product, Piperlongumine (PL), Increases Tumor Cells Sensitivity to NK Cell Killing,” International Immunopharmacology 96 (2021): 15. 10.1016/j.intimp.2021.107658 33887610

[237] B. L. Wang , H. Su , Y. Chen , J. Wang , and G. L. Xu , “A Role for Trichosanthin in the Expansion of CD4CD25 Regulatory T Cells,” Scandinavian Journal f Immunology 71, no. 4 (2010): 258–266. 10.1111/j.1365-3083.2010.02372.x 20384869

[238] H. Zhou , Y. Zhuang , Y. Liang , et al., “Curcumin Exerts Anti‐tumor Activity in Colorectal Cancer via Gut Microbiota‐mediated CD8(+) T Cell Tumor Infiltration and Ferroptosis,” Food Funct 16, no. 9 (2025): 3671–3693. 40244948 10.1039/d4fo04045g

[239] W. Liu , H. Zhou , W. Lai , et al., “Artesunate Induces Melanoma Cell Ferroptosis and Augments Antitumor Immunity Through Targeting Ido1,” Cell Communication and Signaling 22, no. 1 (2024): 378. 39061097 10.1186/s12964-024-01759-8 PMC11282746

[240] C. J. Bao , M. Q. Liu , Y. N. Liu , et al., “A Structurally Distinct Arabinose‐bridged Arabinogalactan From Lycium barbarum as a TLR4‐activating Adjuvant for Nanovaccine‐based Cancer Immunotherapy,” Carbohydrate Polymers 373 (2026): 124618. 41320397 10.1016/j.carbpol.2025.124618

[241] S. Siddiqui and R. Glauben , “Fatty Acid Metabolism in Myeloid‐Derived Suppressor Cells and Tumor‐Associated Macrophages: Key Factor in Cancer Immune Evasion,” Cancers (Basel) 14, no. 1 (2022): 250. 35008414 10.3390/cancers14010250 PMC8750448

[242] Y. C. Huang , S. J. Huang , H. Y. Yang , et al., “Development of a Compound Herbal Formulation (HBK) With Antitumor and Antioxidant Functions for Cancer Adjuvant Therapy,” Phytomedicine 147 (2025): 157212. 40934759 10.1016/j.phymed.2025.157212

[243] G. X. Wei , Y. H. Xu , T. Peng , et al., “Sanguinarine Exhibits Antitumor Activity via Up‐regulation of Fas‐associated Factor 1 in Non‐small Cell Lung Cancer,” Journal Of Biochemical And Molecular Toxicology 31, no. 8 (2017): 8. 10.1002/jbt.21914 28296008

[244] M. J. Saadh , M. A. Mustafa , H. Malathi , et al., “Targeting the Pancreatic Tumor Microenvironment by Plant‐derived Products and Their Nanoformulations,” Medical Oncology 41, no. 8 (2024): 201. 39001987 10.1007/s12032-024-02443-0

[245] M. H. Akhter , S. Kumar , and S. Nomani , “Sonication Tailored Enhance Cytotoxicity of Naringenin Nanoparticle in Pancreatic Cancer: Design, Optimization, and in Vitro Studies,” Drug Development And Industrial Pharmacy 46, no. 4 (2020): 659–672. 32208984 10.1080/03639045.2020.1747485

[246] B. Q. Li , H. L. Shao , L. Gao , et al., “Nano‐drug co‐delivery System of Natural Active Ingredients and Chemotherapy Drugs for Cancer Treatment: A Review,” Drug Delivery 29, no. 1 (2022): 2130–2161. 35815678 10.1080/10717544.2022.2094498 PMC9275501

[247] S. Hashem , T. A. Ali , S. Akhtar , et al., “Targeting Cancer Signaling Pathways by Natural Products: Exploring Promising Anti‐cancer Agents,” Biomedicine & Pharmacotherapy 150 (2022): 12. 10.1016/j.biopha.2022.113054 35658225

[248] H. P. Zhang and Y. S. Li , “Targeting the Breast Tumor Microenvironment by Plant‐derived Products and Their Nanoformulations,” Journal Of Drug Delivery Science And Technology 93 (2024): 20.

[249] H. M. Alharbi , T. Alqahtani , A. H. Alamri , et al., “Nanotechnological Synergy of Mangiferin and Curcumin in Modulating PI3K/Akt/mTOR Pathway: A Novel front in Ovarian Cancer Precision Therapeutics,” Frontiers In pharmacology 14 (2023): 1276209. 38239204 10.3389/fphar.2023.1276209 PMC10794632

[250] M. Mihaila , N. Badea , M. Birliga , et al., “Ginkgo Biloba and Green Tea Polyphenols Captured Into Collagen‐Lipid Nanocarriers: A Promising Synergistically Approach for Apoptosis Activation and Tumoral Cell Cycle Arrest,” International Journal Of Molecular Sciences 26, no. 19 (2025): 26. 10.3390/ijms26199648 PMC12524377 41096913

[251] R. G. Kalinova , I. V. Dimitrov , Y. Ilieva , et al., “Antineoplastic Activity of Podophyllotoxin and Juniper Extracts Encapsulated in MPEG‐b‐PLA Diblock Copolymer Micelles in Cutaneous Squamous Carcinoma Cells,” International Journal of Molecular Sciences 26, no. 11 (2025): 5167. 40507978 10.3390/ijms26115167 PMC12154351

[252] W. Li , Y. Fu , S. Yan , et al., “Construction and Evaluation of pH‐sensitive Nanoparticles With Resveratrol Release to Trigger the Immunogenic Cell Death in Colon Cancer Cellsin Vitro,” Colloids And Surfaces B, Biointerfaces 255 (2025): 114895. 40541034 10.1016/j.colsurfb.2025.114895

[253] X. Zhou , P. Zhang , N. Liu , et al., “Enhancing Chemotherapy for Pancreatic Cancer Through Efficient and Sustained Tumor Microenvironment Remodeling With a Fibroblast‐targeted Nanosystem,” J Control Release 361 (2023): 161–177. 37536546 10.1016/j.jconrel.2023.07.061

[254] M. Dey , M. Das , A. Chowhan , and T. K. Uri , “Breaking the Barricade of Oral Chemotherapy Through Polysaccharide Nanocarrier,” International Journal Of Biological Macromolecules 130 (2019): 34–49. 30779985 10.1016/j.ijbiomac.2019.02.094

[255] N. Wang , J. Hu , L. Jin , et al., “Inulin and Hyaluronic Acid‐based Oral Liposome for Enhanced Photo‐chemotherapy Against Orthotopic Colon Cancer and Its Reversal Effects on Tumor Hypoxia and Intestinal Microbiota,” International Journal Of Biological Macromolecules 304, no. Pt 2 (2025): 140996. 39952512 10.1016/j.ijbiomac.2025.140996

[256] X. Y. Liu , X. Z. Teng , J. H. Wang , et al., “Exosome‐based Mucosal Therapeutic and Diagnostic System: Towards Clinical Translation,” Journal Of Controlled Release 385 (2025): 24. 10.1016/j.jconrel.2025.113966 40553758

[257] S. R. Mallery , G. D. Stoner , P. E. Larsen , et al., “Formulation and in‐vitro and in‐vivo Evaluation of a Mucoadhesive Gel Containing Freeze Dried Black Raspberries: Implications for Oral Cancer Chemoprevention,” Pharmaceutical Research 24, no. 4 (2007): 728–737. 17372698 10.1007/s11095-006-9192-1 PMC2391087

[258] B. Shen , Y. Zhu , F. Wang , et al., “Fabrication and in Vitro/Vivo Evaluation of Quercetin Nanocrystals Stabilized by Glycyrrhizic Acid for Liver Targeted Drug Delivery,” Int J Pharm X 7 (2024): 100246. 38628619 10.1016/j.ijpx.2024.100246 PMC11019285

[259] Y. Li , L. Liu , H. Shang , et al., “Self‐Assembling Anchorage of Hyaluronic Acid on the Nanoparticle Surface Confers Superiority of Triple Negative Breast Cancer Treatment,” Pharmaceutics 14, no. 11 (2022): 2461. 36432652 10.3390/pharmaceutics14112461 PMC9695327

[260] D. Huang , Z. Tang , X. Pu , et al., “A Novel Cabazitaxel Liposomes Modified With Ginsenoside Rk1 for Cancer Targeted Therapy,” Acupuncture And Herbal Medicine 4, no. 1 (2024): 113–121.

[261] X. Han , G. Zhang , X. Wu , et al., “Microfluidics‐enabled Fluorinated Assembly of EGCG‐ligands‐siTOX Nanoparticles for Synergetic Tumor Cells and Exhausted T Cells Regulation in Cancer Immunotherapy,” J Nanobiotechnology 22, no. 1 (2024): 90. 38439048 10.1186/s12951-024-02328-4 PMC10910710

[262] Y. Zhao , Y. Bai , M. Li , et al., “A pH‐triggered N‐oxide Polyzwitterionic Nano‐drug Loaded System for the Anti‐tumor Immunity Activation Research,” J Nanobiotechnology 22, no. 1 (2024): 420. 39014462 10.1186/s12951-024-02677-0 PMC11253471

[263] L. Raimondi , A. De Luca , G. Giavaresi , et al., “Impact of Natural Dietary Agents on Multiple Myeloma Prevention and Treatment: Molecular Insights and Potential for Clinical Translation,” Current Medicinal Chemistry 27, no. 2 (2020): 187–215. 29956610 10.2174/0929867325666180629153141

[264] S. Uramova , P. Kubatka , Z. Dankova , et al., “Plant Natural Modulators in Breast Cancer Prevention: Status Quo and Future Perspectives Reinforced by Predictive, Preventive, and Personalized Medical Approach,” Epma Journal 9, no. 4 (2018): 403–419. 30538792 10.1007/s13167-018-0154-6 PMC6261910

[265] Y. A. Salama , A. El‐Karef , A. M. El Gayyar , and N. Abdel‐Rahman , “Beyond Its Antioxidant Properties: Quercetin Targets Multiple Signalling Pathways in Hepatocellular Carcinoma in Rats,” Life Sciences 236 (2019): 116933. 31614146 10.1016/j.lfs.2019.116933

[266] G. F. He , T. L. Mu , Y. L. Yuan , et al., “Effects of Notch Signaling Pathway in Cervical Cancer by Curcumin Mediated Photodynamic Therapy and Its Possible Mechanisms in Vitro and in Vivo,” Journal Of Cancer 10, no. 17 (2019): 4114–4122. 31417656 10.7150/jca.30690 PMC6692604

[267] F. E. Cheng , X. Zhang , X. Zou , et al., “The Role and Mechanism of Artemisia Annua L. in Cancer Treatment,” Journal Of Ethnopharmacology 353 (2025): 120447. Pt B. 40840727 10.1016/j.jep.2025.120447

[268] S. Xiao , H. Jia , Y. Guo , X. Ding , and A. Zheng , “Chemoprophylactic Effects of Epigallocatechin Gallate in Female Reproductive Cancers—A Review,” J Diet Suppl 22, no. 4 (2025): 487–510. 40518686 10.1080/19390211.2025.2518409

[269] Z. W. Yao and H. Zhu , “Pharmacological Mechanisms and Drug Delivery Systems of Ginsenoside Rg3: A Comprehensive Review,” Pharmacological Research 216 (2025): 107799. 40414584 10.1016/j.phrs.2025.107799

[270] H. Y. Tan , N. Wang , K. Man , et al., “Autophagy‐induced RelB/p52 Activation Mediates Tumour‐associated Macrophage Repolarisation and Suppression of Hepatocellular Carcinoma by Natural Compound baicalin,” Cell Death & Disease 6 (2015): 13. 10.1038/cddis.2015.271 PMC4632300 26492375

[271] Y. Chen , Z. Wang , C. Zhang , et al., “Revealing the Mechanism of Natural Product‐induced Immunogenic Cell Death: Opening a New Chapter in Tumor Immunotherapy,” Frontiers In Immunology 15 (2024): 1470071. 39445013 10.3389/fimmu.2024.1470071 PMC11496055

[272] K. W. Tan , Y. Li , J. W. Paxton , N. P. Birch , and A. Scheepens , “Identification of Novel Dietary Phytochemicals Inhibiting the Efflux Transporter Breast Cancer Resistance Protein (BCRP/ABCG2),” Food Chemistry 138, no. 4 (2013): 2267–2274. 23497885 10.1016/j.foodchem.2012.12.021

[273] L. Liang , J. Wu , J. Luo , et al., “Oxymatrine Reverses 5‐fluorouracil Resistance by Inhibition of Colon Cancer Cell Epithelial‐mesenchymal Transition and NF‐κB Signaling in Vitro,” Oncology Letters 19, no. 1 (2020): 519–526. 31897166 10.3892/ol.2019.11090 PMC6924048

[274] D. Yu , H. Hu , Q. Zhang , et al., “Acevaltrate as a Novel Ferroptosis Inducer With Dual Targets of PCBP1/2 and GPX4 in Colorectal Cancer,” Signal Transduct Target Ther 10, no. 1 (2025): 211. 40619432 10.1038/s41392-025-02296-7 PMC12230175

[275] M. J. Zhang , X. Wan , M. Shi , et al., “Curcuminoids WM03 Inhibits Ovarian Cancer Cisplatin‐resistant Cells Proliferation and Reverses Cisplatin Resistance by Targeting DYRK2,” Phytomedicine 142 (2025): 156632. 40315643 10.1016/j.phymed.2025.156632

[276] T. Chen , Y. Wei , S. Yin , et al., “Construction and Evaluation of BAL‐PTX Co‐Loaded Lipid Nanosystem for Promoting the Anti‐Lung Cancer Efficacy of Paclitaxel and Reducing the Toxicity of Chemotherapeutic Drugs,” Int J Nanomedicine 19 (2024): 7775–7797. 39099795 10.2147/IJN.S474158 PMC11297572

[277] D. Delmas , F. Hermetet , and V. Aires , “PD‐1/PD‐L1 Checkpoints and Resveratrol: A Controversial New Way for a Therapeutic Strategy,” Cancers (Basel) 13, no. 18 (2021): 4509. 34572736 10.3390/cancers13184509 PMC8467857

[278] D. Sun , Y. Zou , L. Song , et al., “A Cyclodextrin‐based Nanoformulation Achieves co‐delivery of Ginsenoside Rg3 and Quercetin for Chemo‐immunotherapy in Colorectal Cancer,” Acta Pharm Sin B 12, no. 1 (2022): 378–393. 35127393 10.1016/j.apsb.2021.06.005 PMC8799998

[279] D. C. da Silva , G. D. C. Orfali , M. G. Santana , et al., “Antitumor Effect of Isoquercetin on Tissue Vasohibin Expression and Colon Cancer Vasculature,” Oncotarget 13 (2022): 307–318. 35145607 10.18632/oncotarget.28181 PMC8823695

[280] P. Goleij , M. M. Heidari , H. Motevalli , et al., “Targeting the HGF/MET Axis in Cancer: Therapeutic Promise for Natural Compounds,” European Journal Of Medicinal Chemistry 298 (2025): 118027. 40752339 10.1016/j.ejmech.2025.118027

[281] Z. W. Wu , X. C. Liu , C. X. Quan , et al., “Novel Galactose‐rich Polysaccharide From Ganoderma lucidum: Structural Characterization and Immunomodulatory Activities,” Carbohydrate Polymers 362 (2025): 123695. 40409828 10.1016/j.carbpol.2025.123695

[282] H. Xiao , Y. Cao , Z. Wang , and C. Liu , “Recent Advances in Astragaloside IV Modulate Immune Response and Skin Cells Promoting Wound Healing,” Tissue Eng Part B Rev (2025). 10.1177/19373341251381830 41051944

[283] E. Abdelhakeem , H. Attia , M. M. Hashem , et al., “Innovative Antimicrobial Nanofibers: Natural Integrations for Enhanced Wound Healing and Biofilm Disruption,” Aaps Pharmscitech [Electronic Resource] 26, no. 6 (2025): 181. 40593407 10.1208/s12249-025-03178-5

[284] Y. Zhou , L. Tao , J. H. Qiu , et al., “Tumor Biomarkers for Diagnosis, Prognosis and Targeted Therapy,” Signal Transduction And Targeted Therapy 9, no. 1 (2024): 86. 38763973 10.1038/s41392-024-01823-2 PMC11102923

[285] C. Y. Fang , C. C. Wu , H. Y. Hsu , et al., “EGCG Inhibits Proliferation, Invasiveness and Tumor Growth by Up‐regulation of Adhesion Molecules, Suppression of Gelatinases Activity, and Induction of Apoptosis in Nasopharyngeal Carcinoma Cells,” International Journal Of Molecular Sciences 16, no. 2 (2015): 2530–2558. 25625511 10.3390/ijms16022530 PMC4346850

[286] Y. Y. Zhao , L. S. C. Dunmall , Z. G. Cheng , Y. H. Wang , and L. L. Si , “Natural Products Targeting Glycolysis in Cancer,” Frontiers In Pharmacology 13 (2022): 18. 10.3389/fphar.2022.1036502 PMC9663463 36386122

[287] M. Ke , Z. Zhang , B. Xu , et al., “Baicalein and baicalin Promote Antitumor Immunity by Suppressing PD‐L1 Expression in Hepatocellular Carcinoma Cells,” International Immunopharmacology 75 (2019): 105824. 31437792 10.1016/j.intimp.2019.105824

[288] L. Wang , X. Y. Liu , H. Z. Lv , et al., “Research Progress on Natural Products That Regulate miRNAs in the Treatment of Osteosarcoma,” Biology‐Basel 14, no. 1 (2025): 26. 39857292 10.3390/biology14010061 PMC11759184

[289] K. El Bairi , A. G. Atanasov , M. Amrani , and S. Afqir , “The Arrival of Predictive Biomarkers for Monitoring Therapy Response to Natural Compounds in Cancer Drug Discovery,” Biomedicine & Pharmacotherapy 109 (2019): 2492–2498. 30551510 10.1016/j.biopha.2018.11.097

[290] C. Liu , Y. Yu , G. Wang , et al., “From Tumor Mutational Burden to Characteristic Targets Analysis: Identifying the Predictive Biomarkers and Natural Product Interventions in Cancer Management,” Frontiers In Nutrition 9 (2022): 14. 10.3389/fnut.2022.989989 PMC9530334 36204371

[291] M. Sharma and T. O. Tollefsbol , “Combinatorial Epigenetic Mechanisms of Sulforaphane, Genistein and Sodium Butyrate in Breast Cancer Inhibition,” Experimental Cell Research 416, no. 1 (2022): 113160. 35447103 10.1016/j.yexcr.2022.113160 PMC12302910

[292] Z. Pu , F. Ge , Y. Wang , et al., “Ginsenoside‐Rg3 Inhibits the Proliferation and Invasion of Hepatoma Carcinoma Cells via Regulating Long Non‐coding RNA HOX Antisense Intergenic,” Bioengineered 12, no. 1 (2021): 2398–2409. 34130594 10.1080/21655979.2021.1932211 PMC8806740

[293] Y. Zhao , L. Jing , L. Han , C. Guo , and Q. Yang , “Coumarins in the Tumor‐immune Application: From Molecular Mechanisms to Therapeutic Innovations,” Frontiers In mmunology 16 (2025): 1681892. 10.3389/fimmu.2025.1681892 PMC12546247 41142809

[294] S. Y. Shen and H. F. Zhuang , “Homoharringtonine in the Treatment of Acute Myeloid Leukemia: A Review,” Medicine 103, no. 44 (2024): 9. 10.1097/MD.0000000000040380 PMC11537654 39496012

[295] S. You , M. J. Wang , Z. Y. Hou , et al., “ACAT1 Induces the Differentiation of Glioblastoma Cells by Rewiring Choline Metabolism,” Int J Biol Sci 20, no. 14 (2024): 5576–5593. 39494339 10.7150/ijbs.96651 PMC11528465

[296] J. L. Huang , L. M. Wu , S. Q. Wu , et al., “A Small Molecule Targets LIC1 to Suppress Lung Tumor Growth by Inducing Autophagy,” Nature Chemical Biology (2025). 10.1038/s41589-025-02040-w 41102409

[297] J. Peterson , P. Lagiou , E. Samoli , et al., “Flavonoid Intake and Breast Cancer Risk: A Case‐control Study in Greece,” British Journal Of Cancer 89, no. 7 (2003): 1255–1259. 14520456 10.1038/sj.bjc.6601271 PMC2394299

[298] J. Wu , C. Shao , Y. Dong , et al., “Qu‐yu‐jie‐du Decoction Maintenance Therapy Improves Postoperative Survival in Metastatic Colorectal Cancer: A Single‐center Randomized Trial With 36‐month Follow‐up,” Phytomedicine 146 (2025): 157143. 40815944 10.1016/j.phymed.2025.157143

[299] Y. Yang , W. Ge , W. Luo , et al., “Effects of Chinese Herbal Medicine on the Secondary Prevention of Chemotherapy‐induced Thrombocytopenia in Malignant Solid Tumors, a Randomized Clinical Trial,” Phytomedicine 144 (2025): 156871. 40505481 10.1016/j.phymed.2025.156871

[300] R. Sadahiro , K. Ohbuchi , T. Nakaya , et al., “Metabolome Profiling of Yokukansan in Preventing Postoperative Delirium in Elderly Cancer Patients: A Reverse Translational Study,” Psychiatry And Clinical Neurosciences 79, no. 10 (2025): 685–696. 40765131 10.1111/pcn.13875 PMC12498125

[301] C. Y. Wang , X. Y. Lan , J. X. Gu , et al., “Application of Functionalized Liposomes in the Delivery of Natural Products,” Progress In Biochemistry And Biophysics 51, no. 11 (2024): 2947–2959.

[302] J. J. Peng , L. Wang , M. G. Wang , et al., “Yeast Synthetic Biology for the Production of Lycium Barbarum Polysaccharides,” Molecules (Basel, Switzerland) 26, no. 6 (2021): 9. 10.3390/molecules26061641 PMC8000229 33804230

